# Detection of Regulatory SNPs in Human Genome Using ChIP-seq ENCODE Data

**DOI:** 10.1371/journal.pone.0078833

**Published:** 2013-10-29

**Authors:** Leonid O. Bryzgalov, Elena V. Antontseva, Marina Yu. Matveeva, Alexander G. Shilov, Elena V. Kashina, Viatcheslav A. Mordvinov, Tatyana I. Merkulova

**Affiliations:** 1 Institute of Cytology and Genetics SD RAS, Novosibirsk, Russian Federation; 2 Novosibirsk State University, Novosibirsk, Russian Federation; LuonanChinese Academy of Sciences, China

## Abstract

A vast amount of SNPs derived from genome-wide association studies are represented by non-coding ones, therefore exacerbating the need for effective identification of regulatory SNPs (rSNPs) among them. However, this task remains challenging since the regulatory part of the human genome is annotated much poorly as opposed to coding regions. Here we describe an approach aggregating the whole set of ENCODE ChIP-seq data in order to search for rSNPs, and provide the experimental evidence of its efficiency. Its algorithm is based on the assumption that the enrichment of a genomic region with transcription factor binding loci (ChIP-seq peaks) indicates its regulatory function, and thereby SNPs located in this region are more likely to influence transcription regulation. To ensure that the approach preferably selects functionally meaningful SNPs, we performed enrichment analysis of several human SNP datasets associated with phenotypic manifestations. It was shown that all samples are significantly enriched with SNPs falling into the regions of multiple ChIP-seq peaks as compared with the randomly selected SNPs. For experimental verification, 40 SNPs falling into overlapping regions of at least 7 TF binding loci were selected from OMIM. The effect of SNPs on the binding of the DNA fragments containing them to the nuclear proteins from four human cell lines (HepG2, HeLaS3, HCT-116, and K562) has been tested by EMSA. A radical change in the binding pattern has been observed for 29 SNPs, besides, 6 more SNPs also demonstrated less pronounced changes. Taken together, the results demonstrate the effective way to search for potential rSNPs with the aid of ChIP-seq data provided by ENCODE project.

## Introduction

Single nucleotide polymorphisms (SNPs) represent the most common type of sequence variation. Recently, the advance in high-throughput DNA sequencing methods has provided a rapid growth in the volume of information about the saturation of genomes with SNPs. For example, the NCBI dbSNP in 2005 contained slightly over ten million SNPs in the human genome [1], while at the moment when this study was commenced, their number exceeded 45 million. It is likely that most SNPs lack any functional significance. However, a small part of these substitutions can have certain phenotypic manifestations appearing as changes in the structure of the protein product of a gene or the level of its expression and in turn some of these may be associated with various diseases [2]; [3]. Currently, three groups of functionally significant SNPs are distinguished, namely, cSNPs, rSNPs and sSNPs, which are localized to the coding, regulatory, and splicing-relevant regions of human genes, respectively [4]; [5]; [6]; [7]. The cSNPs are most intensively studied, since they are easily detectable in well-annotated protein-coding sequences of human genome and relatively easy interpretable from the functional standpoint. State-of-the-art bioinformatics methods make it possible to identify not only the SNPs that alter a protein amino acid sequence, but also those located in the known functional protein domains and altering protein functions [8]; [9]; [10], which enhances selection of the candidates for further functional validation. Thus, cSNPs represent the main content of the databases on human gene mutations of pathological significance. In particular, cSNPs [7] account for 86% of the total number of the mutations (∼90,000) compiled in HGMD—the central disease-associated human gene mutation database [6]. The sSNPs are the second with respect to the degree of our knowledge. The mutations located within exon–intron splice junction sites represent ∼10% of all the reported SNPs logged in HGMD [7]. Despite an evident functional significance, the group of rSNPs, which unites the mutations able to influence transcription initiation, elongation, and translational characteristics of mRNA, is least represented in databases. In particular, this joint group constitutes only 3% of the HGMD dataset [7].

Of special interest among the rSNPs are the polymorphisms localized to the binding sites of various transcription factors (TFs; TFBSs). Such rSNPs can exert a functional effect by altering the regulation of gene transcription. This is explainable with a corresponding increase or decrease in the binding of a given TF, leading to allele-specific gene expression. In some cases, rSNPs may eliminate an existing binding site and/or generate a binding site for another TF, which can have a dramatic effect on the gene expression pattern. There are numerous examples of such rSNPs associated with various diseases. In particular, the substitution of −30 T>A in the TATA box of human beta-globin gene (HBB) promoter leads to a fourfold decrease in the TBP/TATA affinity [11] and a decrease in the beta-globin mRNA content to 8–13% of the norm in β-thalassemia patients [12]. On the contrary, the AFP gene promoter in the case of hereditary persistence of α-fetoprotein carries two substitutions (−119 G>A and −55 C>A) in its HNF1 binding sites, which increase both the affinity towards HNF1 and the level of gene transcription [13]. Similarly, polymorphism at position −2578 A>G of CCL2 distal promoter creates additional binding site for PREP1/PBX2 transcription factors causing by stimulation of the promoter activity and inflammation [14]. In contrast, the reported GWAS SNP rs6801957:G>A in the SCN10A enhancer disrupts TBX3/TBX5 binding and reduces the tissue-specific activity of the enhancer in the heart [15]. Each of the two substitutions 663 G>A and 666 G>T in intron 2 of the human TDO2 gene, which are associated with a number of psychiatric disorders [16], leads to destruction of YY-1 binding site with creation of the binding sites for unknown transcription factors [17]. In turn, −1514 C>T SNP in the TBX21 promoter associated with systemic lupus erythematosus in statistic studies reduces the USF-1 affinity and enhances the transcription activity [18]. The minor allele (T) of a common noncoding polymorphism at the 1p13 cholesterol locus, rs12740374, creates a C/EBP binding site, that results in 12-fold increase of liver SORT1 expression leading to decreased LDL-C and very small LDL particles and lower risk of myocardial infarction [19].

Among rSNPs, currently registered in the different databases, the most are polymorphisms localized to promoter regions of genes [6]; [7]; [20]; [21]. A large-scale search for potential rSNPs using computer methods based on identification of TFBSs has also involved only promoter regions [22]; [23]; [24]. This is the result of relatively good promoter mapping in genome scale owing to exact positions of transcription start sites (TSS) determined by experimental approaches [25]. The search of remote gene regulatory regions for rSNPs is very difficult due to the absence of regular patterns in localizations and sizes of distal regulatory units (enhancers, silencers, and LCRs). Since at the DNA level gene regulatory regions represent clusters of TFBSs [26]; [27] computation approaches based on the search of TFBSs clusters were elaborated [28]; [29]; [30]. Despite of these approaches led to discovery of some functional enhances [29]; [31], their genome wide application is problematic because of too many false-positives [30]; [32]. Therefore, the problem of annotating the regulatory part of the genome is yet at the very beginning of its solution.

Chromatin immunoprecipitation (ChIP) technique with subsequent microarray (ChIP-chip) or massively parallel sequencing (ChIP-seq) is a powerful approach, which enables a genome-scale mapping of transcription factor occupancy pattern in a given cell type and state [33]. So far, this approach has allowed for detection of thousands of binding loci for a number of TFs in the chromatin of various cells, the majority of which are localized to the extragenic regions distant from the transcription start site and intragenic regions [34]; [35]; [36]. In principle, comparative analysis of genome-wide distribution of such binding loci for different TFs is able to identify clusters of them and consequently cis-regulatory elements, without any genome structure limitations [27]. However, the data obtained in every ChIP-seq experiment demonstrate TF binding specific to cell line or tissue used and strongly dependant on environmental conditions [37]; [38]; [39]. Moreover, the loss of some tissue-specific features (for example as a result of cell immortalization) or different environmental changes may alter the genome wide pattern of TF binding inherent to differentiated cells of living organism [33]; [40]. Since it is known that specific spatial-temporal patterns of gene expression is controlled by combinatorial binding of different TF sets to regulatory units [27]; [41]; [42] it seems promising to search for these regions by integrative analysis of as many as possible of different ChIP-seq data. Under the ENCODE project, a huge amount of data on transcription factor binding in diverse human cell types under different states has been accumulated to date [43]. So the goal of this work was to elaborate a novel approach to the search for rSNPs in human genome using available ChIP-seq ENCODE data. We proposed that since the enrichment of a particular genomic region by ChIP-seq peaks of various TFs is indicative of its regulatory function and thus, SNPs localized within this region are likely to affect TF binding sites and thereafter possess a regulatory potential. The efficiency of such approach for potential rSNP identification was then demonstrated *in silico* by comparison of samples of clinically associated and randomly selected human SNPs and experimentally confirmed on a sample of SNPs from OMIM catalog.

## Materials and Methods

### Genomic Features

Two major sources of data were used in this study:

1. Genomic data on the locations of SNPs in the human genome were downloaded from database dbSNP NCBI http://www.ncbi.nlm.nih.gov/snp/(Human Genome Build 37, dbSNP build 137). Sample S_clinic_ consisting of 34,373 clinically associated SNPs was selected from dbSNP NCBI (http://www.ncbi.nlm.nih.gov/snp/limits) by limits criteria “organism: Homo sapiens” and “annotation: Clinical/LSDB Submissions”. Sample S_omim_ composed of 18,291 SNPs was also selected from database dbSNP NCBI in criteria “organism: Homo sapiens” and “annotation: OMIM”. Sample S_R_ contains 1,000,000 randomly selected SNPs from 30,249,489 SNPs founded in the 1000-Genomes Project (dbSNP NCBI; limits criteria “Validation Status: by-1000 Genomes”). Sample S_gwas_ comprising 10,345 SNPs which were phenotypically associated with the *p*-values below 1 e^−5^ was selected from the NHGRI GWAS catalog (http://www.genome.gov/gwastudies/).
2. Chromosome coordinates of loci where TFs bind to DNA as assayed by ChIP-seq were downloaded from the UCSC website (http://genome.ucsc.edu/) page “ENCODE Txn Factor ChIP track” (ENCODE project; Date submitted 2012-05-25 http://hgdownload.cse.ucsc.edu/goldenPath/hg19/encodeDCC/wgEncodeRegTfbsClustered/). The ENCODE TF binding data were utilized to extract genomic regions containing 2 or more (*i*) overlapping TF binding loci, referred to as OTFR(*i*) (Document S1). Gaussian approximation of the ChIP-seq peak density was carried out and 2σ area (threshold of 95%) was taken to construct the OTFR. For each of SNP samples mentioned above, SNPs falling into OFTR were determined as putative rSNPs, and the enrichment *E*(*i*) of sample by such SNPs was calculated for each value of *i*>2.

### Statistical analysis

Estimates of standard deviations and confidence intervals for enrichment analysis were derived by bootstrapping procedure (Figure S1). Briefly, for each of the analyzed samples of both OTFR(i) and SNPs (S_omim_, S_clinic_, S_gwas_, S_r_, etc.) 500 random samples were generated by resampling (In house Perl script is available as Document S2). That means the resulting samples were the same size as the initial sample, but some elements were selected repeatedly while some were excluded by chance. Then *E*(*i*) was calculated for each pair of resulting SNP and OTFR(i) samples. To test the hypothesis of normal distribution of the data (random error) Pearson's chi-squared test (χ2) was used. It was found that for all samples the distribution was close to normal (p-Value >0.999).

### Cell Lines

Human hepatoma cell line HepG2, human cervical adenocarcinoma cell line HelaS3, human colorectal cancer cells HCT-116, human erythromyeloblastoid leukemia cell line K562 were grown in DMEM/F12 (GIBCO) containing 10% fetal calf serum (Thermo Scientific HYCLONE), 100 units/ml penicillin, and 100 mg/ml streptomycin (Sigma) under 5% CO_2_ at 37°C.

### Preparation of Nuclear Extracts

Cells (∼10^7^) were washed with ice-cold PBS and then pelleted by centrifugation at 300 g for 2 min. The cell pellet was resuspended in 1 ml of Buffer 1 [10 mM HEPES, pH 7.9, 10 mM KCl, 1 mM dithiothreitol, 0.5 mM spermidin, 0.15 mM spermin, 0.1 mM EDTA, 0.1 mM EGTA, 0.5 mM PMSF, 1× Halt protease inhibitor cocktail (Thermo Scientific)] and placed on ice to swell for 15 min. After addition of 62 µl of 10% (w/v) Nonidet P-40, the sample was gently vortexed for 10 s and then centrifuged at 400 g for 5 min at 4°C. The nuclear pellet was resuspended in 100 µl of Buffer 2 [20 mM HEPES, pH 7.9, 25% (w/v) glycerol, 420 mM NaCl, 1.5 mM MgCl_2_, 0.2 mM EDTA, 1 mM dithiothreitol, 1× Halt protease inhibitor cocktail (Thermo Scientific)] and incubated for 20 min on ice followed by a centrifugation at 10,000 g for 10 min at 4°C. The supernatant containing the nuclear proteins was stored at - 70 C.

### Electrophoretic Mobility Shift Assays

Table S1 lists the sequences of oligonucleotides used for EMSA tests. For each allelic version double stranded oligonucleotides V1 and V2 were synthesized with 5′-CAGT tetranucleotide overhangs and SNP position in the center. Introduction of labels in the DNA probe was conducted using fill in of shortened 3′ ends of Klenov fragments of DNA polymerase I. The reaction was conducted for 5 min at room temperature in 10 µl of reaction mixture which contains 0.01 mM oligonucleotides, 1 µl 10× buffer for labeling (500 mM Tris-HCl, pH 8.0, 100 mM NaCl, 100 mM MgCl_2_, 1 mM DTT, 2 mM dGTP, 2 mM dTTP, 2 mM dCTP), 2 active units of Klenov fragment, and 10 µCi (α-32P)dATP.

The protein nuclear extract was incubated with sheared salmon sperm DNA (1 µg of DNA per 7 µg of total protein) for 10 min at 0°C. After that 4 µg of extract was added to the probes which contain 50 pM of radioactive labeled oligonucleotide. After incubation at room temperature for 15 min the mixture was subjected to electrophoresis in 4.5% PAAG in 0.5×TBE (89 mM Tris-borate, 89 mM H_3_BO_3_, 2 mM EDTA at 4°C). The gel was exposed with X-ray film. Two to three independent replicates were performed for each EMSA experiment.

## Results

### Description of Approach

**Figure 1:**
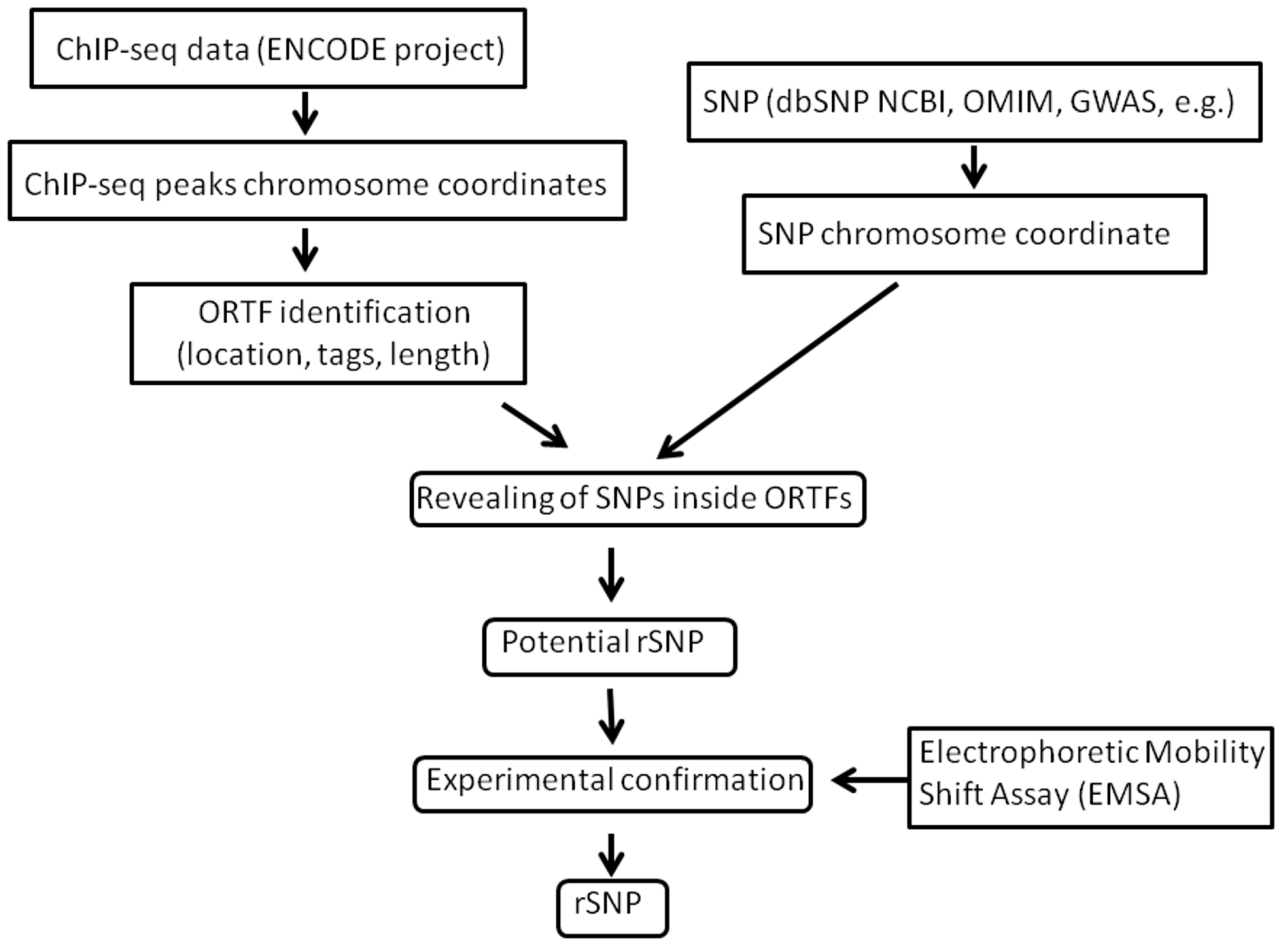
The used approach to genome-wide selection of rSNPs. Computational analysis was applied to identify the SNPs in the most likely regulatory regions of the human genome and predict rSNPs for experimental verification.

The scheme of our approach is shown in Figure 1. The ChIP-seq database of the international ENCODE project contains the information about distribution of 2.75 million of ChIP-seq peaks (human genome build 37) for 134 TFs in the genomes of one or several of 70 human cell lines. The presence of a peak means that the corresponding DNA region houses a TFBS, and the enrichment of a genomic region for ChIP-seq peaks for various TFs indicate that this is a putative regulatory region. Therefore, this suggests that the SNP localized to this region is able to be associated with a certain TFBS contained in it or creates a new site and thus is able to influence transcription regulation.

Based on this reasoning, dataset about regions where TF bind to DNA as assayed by ChIP-seq were downloaded from “ENCODE Txn Factor ChIP track” and utilized to extract genomic regions containing 2 or more overlapping TF binding loci, referred to as OTFR. OTFRs were further considered as regulatory regions, and SNPs falling into OTFR were defined as putative regulatory polymorphisms (rSNPs) requiring experimental verification. Electrophoretic mobility shift assay (EMSA) was used for this purpose as an inexpensive and appropriate method, which allows for estimation of the SNP effect on both the efficiency of TF binding and the range of the bound proteins.

### *In silico* Testing of the Approach in Samples of Clinically and Phenotype Associated and Randomly Selected Human SNPs

To test the operation efficiency of the proposed approach we analyzed the enrichment of different phenotype-associated SNP samples with putative rSNPs. For this purpose, three samples were constructed: 1) sample S_clinic_ comprised 34,373 clinically associated SNPs, 2) sample S_omim_ included 18,291 SNPs cataloged in OMIM, and 3) sample S_gwas_ consisting of 10,345 SNPs from the NHGRI GWAS catalog containing SNP with trait association p-values below 1 e–5 [44]. As an independent control, we used sample S_r_ consisting of 1,000,000 SNPs randomly selected from over thirty millions SNPs founded in the 1000-Genomes Project.

The enrichment of samples with putative rSNPs was calculated as follows: 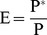
where P* is the observed probability of finding a putative rSNP in the analyzed sample; *P*, probability of finding an rSNP in the case of uniform genomic distribution.

The probability P* was calculated as ratio of the number of SNPs falling into OTFRs to the total number of SNPs in the analyzed sample: 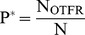



The probability P was determined as ratio of total length of OTFRs to the genome length (3*10^9^ bp): 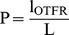



The enrichment was calculated for a series of OTFR(*i*) samples, where *i*>2 is the minimum allowed number of overlapping TF binding loci for OTFR. Table S2 and Figure S2 demonstrate total length of OTFR(*i*) and proportion of the genome falling into OTFRs, depending on the *i* values.

Thus the final equation was as follows: 
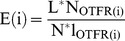



500 iterations of bootstrap resampling were conducted for each pair of OTFR(*i*) and SNP samples (Figure S1) in order to obtain the estimates of standard deviations and confidence intervals for the enrichment analysis (Table S3).

**Figure 2:**
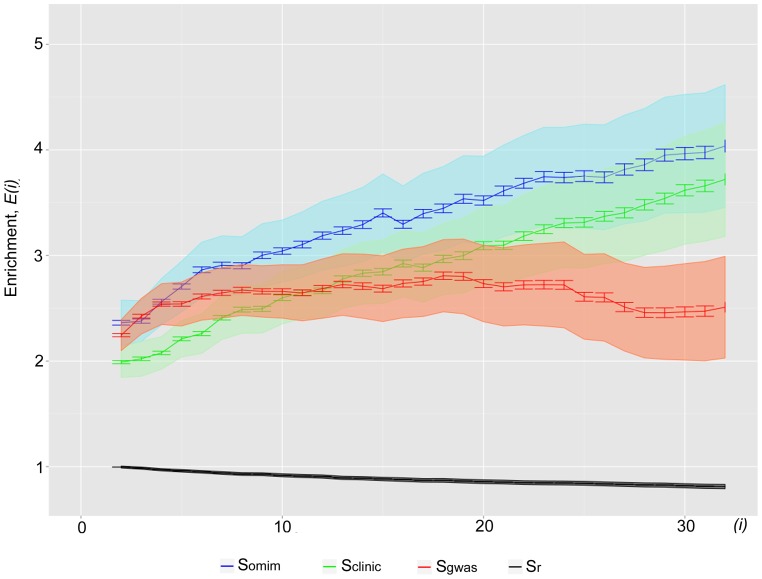
The enrichment of functionally-associated S_omim_, S_clinic_, S_gwas_ samples, and S_r_ sample with putative rSNPs as a function of cut-off number of overlapping TF binding loci (*i*) for defining OTFRs. 500 bootstrap iterations were performed for each point. The resulting standard deviations and confidence intervals are shown by error bars and colour-filled areas,respectively. S_clinic_, S_omim_, and S_gwas_ consist of SNPs associated with phenotypic manifestations and extracted from dbSNP NCBI Clinical/LSDB Submissions Resources, OMIM catalog, and NHGRI GWAS catalog, respectively. Sample S_r_ of random SNPs was created without applying any phenotypic preferences and used as control. Genomic region chr6:29,909,708-31,325,212 was excluded from the analysis of S_clinic_ sample, since it caused enrichment overestimation at lower *i* (a large number of SNPs concentrate in this region due to its extensive use in genotyping assays).

It is evident from the plot in Figure 2 that the higher *i* threshold, the larger is the enrichment of S_clinic_ and S_omim_ samples with putative rSNPs. In particular, *E*(30) was 4±0.07 (here and further are 99% confidence intervals for the *E*(*i*) means) for S_omim_ and 3.6±0.07 for S_clinic_, corresponding to 4.9- and 4.4-fold enrichment relative to control sample S_r_ at *i* = 30. In contrast, there is no enrichment of random sample S_r_ with putative rSNPs, rather 1.2-fold depletion is seen at *i* = 30 (*E*(30) = 0.84±0.003). Thus, with increasing allowed number of overlapping TF binding loci in OTFRs, the fractions of putative rSNPs shows increasing difference between phenotype-associated and control samples.

Intriguing results have been also obtained when applying our approach to the samples of SNPs from the NHGRI GWAS catalog [44]. Initially, we performed the enrichment analysis of full NHGRI sample S_gwas_ consisting of 10,345 SNPs. It is evident from Figure 2 that the *E*(*i*) value for S_gwas_ is almost independent of the *i* value in contrast to S_clinic_ and S_omim_, displaying 2.6–3.3-fold enrichment for rSNPs relative to S_r_.

**Figure 3.The:**
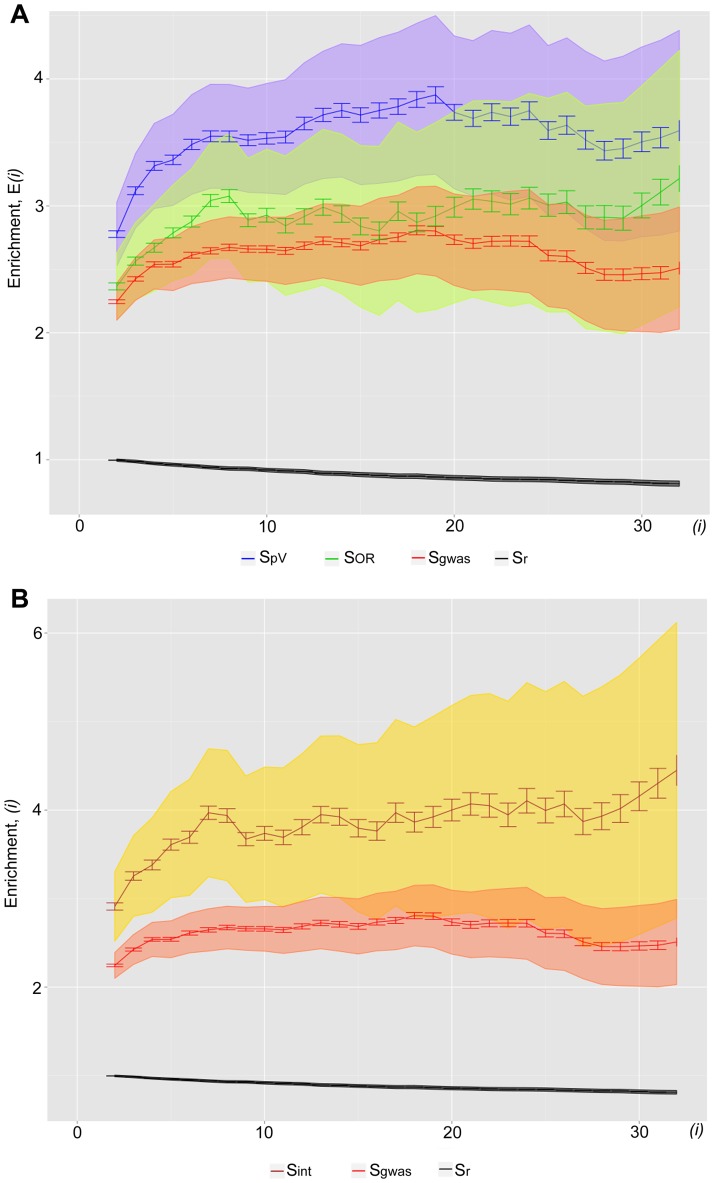
enrichment of S_gwas_ sample and its high-confidence derivatives, S_pV_, S_OR_ and S_int_, as well as S_r_ sample with putative rSNPs as a function of cut-off number of overlapping TF binding loci (*i*) for defining OTFRs. 500 bootstrap iterations were performed for each point. The resulting standard deviations and confidence intervals are shown by error bars and colour-filled areas,respectively. The subsamples of S_gwas_ were generated with filtering of SNPs by P-value <1 e–7 (S_pV_), OR>3 and <0.3 (S_OR_), and by both criteria (S_int_). S_gwas_ sample was extracted from NHGRI GWAS catalog.

We further tested whether additional filtering of S_gwas_ sample by *p*-value and OR (odds ratio) parameters reflecting the significance of SNP-trait association could improve the observed enrichment. Since under the terms of NHGRI GWAS catalog all SNPs from S_gwas_ already have trait association p-values below 1 e–5, we constructed sample S_pV_ consisting of 5,115 SNPs with the *p*-values below 1 e^−7^, and sample S_OR_, which comprises 3,084 SNPs associated with a certain disease with OR>3 and OR<0.33. As could be seen from the Figure 3A, noticeably higher enrichment of newly generated samples with putative rSNPs is observed compared to the original S_gwas_ sample. The highest enrichment was obtained for S_int_ sample (Fig. 3B) that contains only 1,850 SNPs with both OR and *p*-value filtering, e.g. at *i* = 32 it reached 4.9-fold relative to control S_r_ sample. Hence the use of stricter filtering of S_gwas_ sample by significance values produces functionally more relevant SNP sample which is comparable to S_clinic_ and S_omim_ datasets. These results allow us to assume that not all SNPs from NHGRI GWAS directory cause phenotypic manifestation and most likely some of them are identified due to linkage with causal SNPs.

The computed enrichment of the SNP samples associated with phenotypic manifestations for the rSNPs predicted by our method suggests that many of such rSNPs indeed have a certain functional significance. Since these SNPs are located in predicted regulatory regions of genes, it is likely that they are associated with the binding sites of certain TFs and thus influence the regulation of gene expression, which is likely to enhance the development of various pathologies.

### Experimental Verification of Putative rSNPs by EMSA

**Figure 4:**
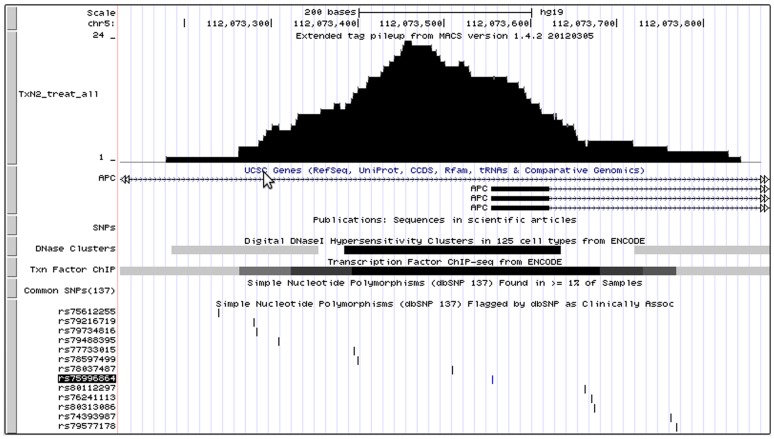
Localization of putative rSNPs within OTFR belonging to APC gene. SNPs from the different parts of OTFR were taken in EMSA to study the effect of SNP location within OTFR on the protein binding.

For experimental verification, 30 non-coding SNPs falling in OTFRs with *i*≥7 (Document S3) were randomly selected from the S_omim_ sample. Additionally, to study the effect of SNP location within OTFR 12 SNPs from the same OTFR were selected (Fig. 4). A total of 40 SNPs were analyzed by EMSA. It has been shown that 27 of these SNPs are located in gene introns (e.g., one intronic SNP also falls into promoter of another gene); 12 SNPs are in 5′-UTRs (one of them also falls into the promoter of reversely oriented gene); and one, in 3′-UTR. For each of these SNPs, double-stranded oligonucleotides corresponding to different alleles, V1 and V2, were synthesized (Table S1). These oligonucleotides have been used as DNA probes in the EMSA experiments with proteins of different nuclear extracts obtained from four human cell lines (HepG2, HeLaS3, HCT-116, and K562) (http://lungry1.bionet.nsc.ru/cgi-bin/SNPProject/SNPChIPTools.cgi). These lines are most frequently used in the ChIP-seq experiments of the ENCODE project and have different tissue origins, which expanded the range of nuclear proteins able to bind to specific DNA sites. Two to three independent replicates were performed for each EMSA experiment.

**Figure 5:**
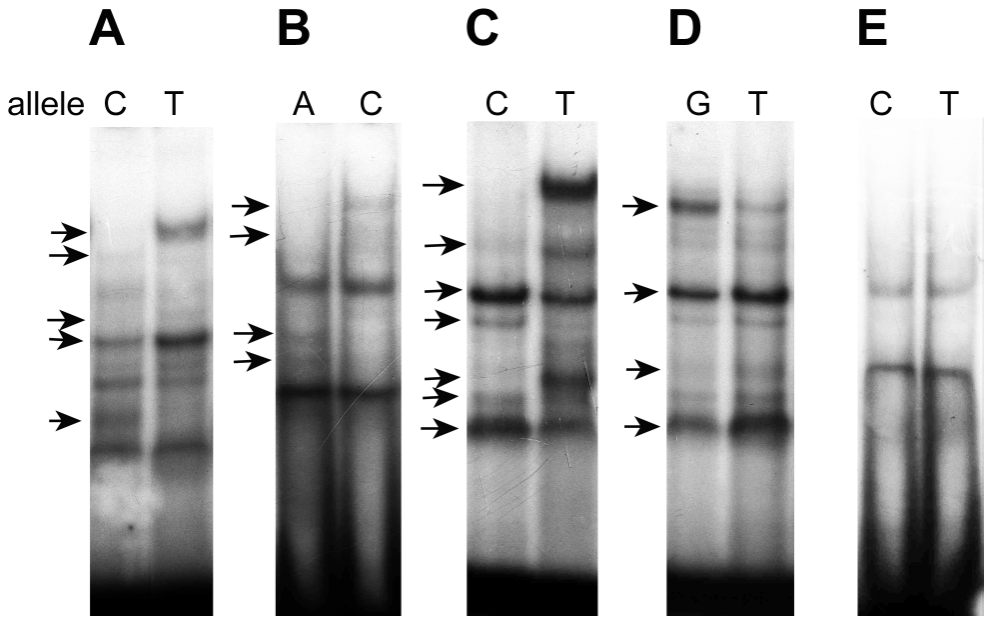
A variety of SNP effects on binding of the corresponding oligonucleotides to nuclear proteins from K562 cells. rs79734816:C>T (*A*), rs2071002:A>C (*B*), and rs74393987:C>T (*C*) change the number and intensity of bands, while rs75996864:G>T (*D*) affect only band intensity, and 7961894:C>T (*E*) do not have any. Changes in the binding of allelic variants with the nuclear proteins are indicated by arrows.

**Figure 6:**
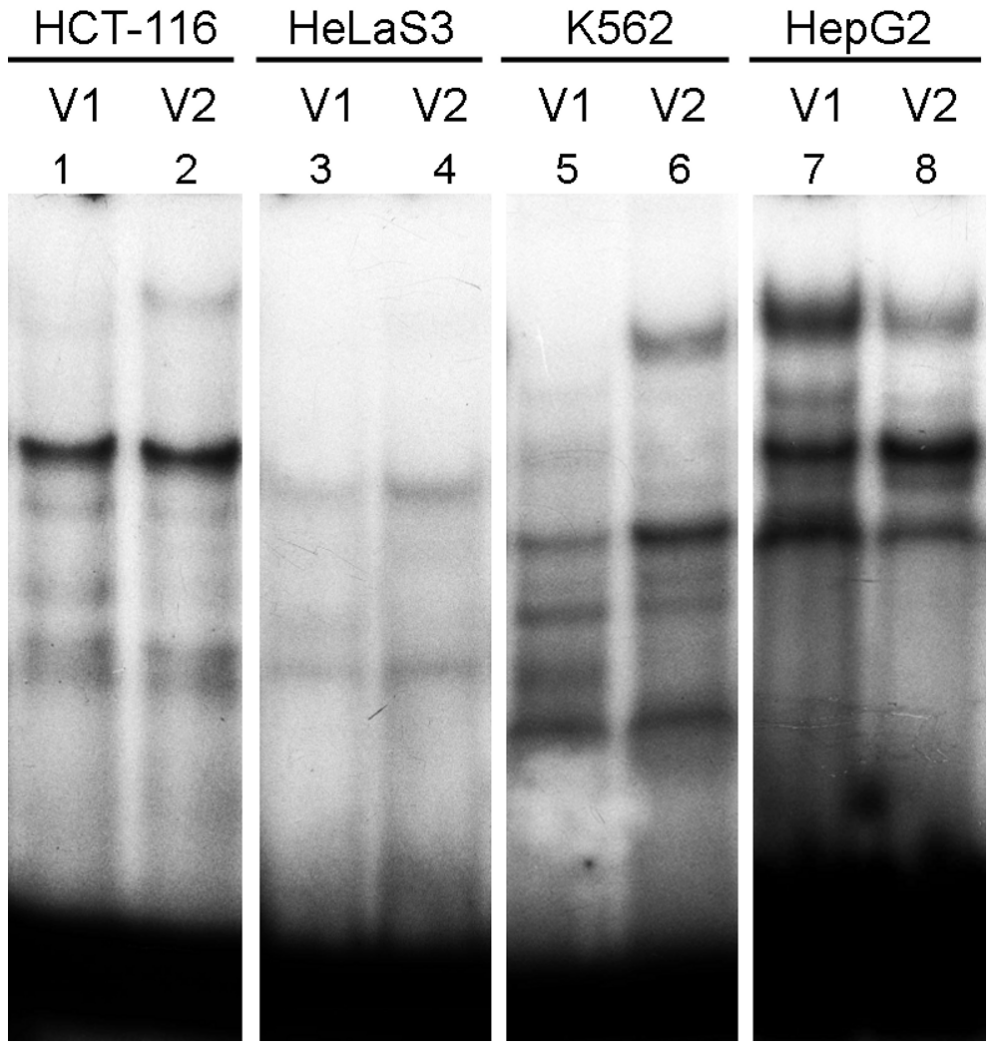
Cell line-dependent binding of nuclear proteins to oligonucleotide probes. Binding of nuclear proteins isolated from HCT-116 (*1*, *2*), HeLaS3 (*3*, *4*), K562 (*5*, *6*) and HepG2 (*7*, *8*) human cell lines to the V1 (C- allele) and V2 (T- allele) oligonucleotides corresponding to allelic versions of rs79734816:C>T.

Tables 1 and 2 consolidates the EMSA data for the nuclear extracts of different cell lines. It has been shown that the substitution of only one nucleotide distinguishing V1 from V2 oligonucleotides for 35 SNPs leads to changes in binding of the DNA probe to nuclear extract proteins of at least one cell line. In particular, 25 of these SNPs lead to a complete disappearance of certain bands in autoradiogram and/or emergence of other bands with different mobility. This suggests that the SNPs in question are able to destroy the binding sites for some TFs and/or create the binding sites for other TFs and, an appropriate situation provided, influence the regulation of gene expression at a transcriptional level. Examples of several such SNPs with different variants of changes in binding patterns are shown in Figure 5 (*A*, *B* and *C*). For other 10 SNPs of the found 35, the corresponding allelic variants V1 and V2 form the retardation bands with equal mobility (example in Figure 5, *D*) but different intensities, which may suggest impairment/weakening of the binding sites for certain TFs. The remaining five SNPs of the 40 tested in the experiment either had no effect on binding or the corresponding oligonucleotides did not bind to any proteins from the nuclear extracts of the selected cell lines (example in Figure 5, *E*). However, it cannot be excluded that these SNPs will behave as rSNPs when using the nuclear extract of another human cell line or, even better, a biopsy specimen. The fact that the dependence of binding pattern not only on the SNP allelic variant, but also on the used cell line has been demonstrated for several of the tested SNPs also confirms this assumption. Figure 6 shows an example of such SNP (rs79734816:C>T): the corresponding V1 DNA probe C, as well as V2 T, gives different binding patterns with the protein extracts of all the used cell lines; in addition the effect of SNP on the binding is unique for each extract.

**Table 1:** Summary of the experimental testing of putative rSNPs selected from S_omim_.

SNP identifier in dbSNP NCBI	Qty. of ChIP-seq peaks	HCT-116	HelaS3	HepG2	K562
rs2010963	11/111	2	2	2	1
rs2279744	34/110	3	3	3	3
rs11466315	12/103	2	2	1	1
rs1800734	41/94	2	1	1	1
rs17039192	7/76	1	2	1	1
rs12885713	71/59	3	3	2	3
rs2071002	19/57	3	1	2	3
rs2297339	40/54	2	2	2	2
rs3807306	27/46	2	3	1	1
rs1048990	28/43	3	1	3	3
rs737865	13/36	3	1	3	3
rs80313086	20/33	2	2	3	1
rs79734816	4/33	3	3	3	3
rs74393987	4/33	3	3	3	3
rs12740374	23/32	3	2	1	1
rs55853698	19/27	3	2	1	1
rs4821544	23/26	1	1	1	1
rs11178998	28/25	1	1	1	1
rs2282978	21/24	3	3	3	3
rs3766379	1/23	1	1	1	3
rs2038137	2/21	1	2	2	1
rs6958571	14/21	3	3	2	3
rs12044852	4/20	1	1	1	1
rs10411210	18/20	1	1	2	1
rs4809324	8/16	3	1	2	3
rs3057	1/15	1	1	1	0
rs1532624	5/13	2	1	3	1
rs9465871	7/9	1	2	1	2
rs113994210	7/7	3	3	1	1
rs7961894	7/7	ND	1	1	1

1- no influence, 2- influence of SNP on the band intensity, 3- influence of SNP on the band mobility.

**Table 2:** Summary of the experimental testing of putative rSNPs from different parts of OTFR belonging to APC gene.

SNP identifier in dbSNP NCBI	Qty. of ChIP-seq peaks	HCT-116	HelaS3	HepG2	K562
rs75996864	26/33	2	2	3	2
rs78037487	26/33	1	3	1	ND
rs80313086	20/33	2	2	3	1
rs76241113	20/33	3	2	1	2
rs80112297	19/33	3	3	3	3
rs78597499	16/33	1	2	1	2
rs77733015	15/33	1	1	2	1
rs79488395	8/33	2	3	2	3
rs79734816	4/33	3	3	3	3
rs79216719	4/33	3	2	2	3
rs79577178	3/33	3	3	3	3
rs75612255	2/33	3	1	3	3

1- no influence, 2- influence of SNP on the band intensity, 3- influence of SNP on the band mobility.

Thus, the EMSA tests has allowed us to validate a regulatory potential for 29 of the predicted rSNPs and to demonstrate that additional 6 polymorphisms with less pronounced changes are also likely to be regulatory. Additionally some position effect of SNP within OTFR was shown. Although all 12 SNPs from different parts of OTFR belonging to APC gene demonstrated profound changes in protein binding pattern, strong tissue-specific effect was observed (Table 2).

All sequences containing experimentally verified SNPs were subjected to the search for putative TF binding sites, which appeared and/or were disrupted due to single nucleotide substitution, using transcription factor binding site profiles from open-access JASPAR database. For this purpose TFBSs were predicted for both sequence variants with the relative profile score threshold of 80% and then motifs represented only in one of them as well as motifs displaying score difference above 5% between variants were extracted. As could be seen from the Table S4, most of SNPs (even not confirmed to be regulatory in EMSA) were predicted to affect several JASPAR motifs simultaneously although some experimentally confirmed rSNPs didn't influence on the presence and score of predicted TFBSs. This could be due to both incomplete representation of TFBSs in JASPAR and high under- or over-prediction rates for the annotated profiles, the main problem in TFBS recognition [40]; [45].

## Discussion

It is known that the changes in TF binding to the cognate sites can lead to dramatic consequences down to cell death or malignant transformation due to impairments in cell differentiation and proliferation [46]; [47]; [48]. Appearance of a new TFBS or, on the contrary, its destruction resulting from a single nucleotide substitution can change the “regulatory code” of a gene or local chromatin architecture in the corresponding region and in certain cases lead to development of pathologies. Thus, the rSNPs may be a cause of diseases, and their detection is in demand, especially for personalized medicine. However, a genome-wide search for rSNPs is still an unsolved problem, although there are several pioneer works devoted to identification of rSNPs affecting particular TFBSs, namely p53 and USF1 binding sites [49]; [50]. For a long period of time, the only tool for a large-scale search of putative rSNPs were computational methods for TFBSs recognition (mainly, with the help of weight matrices). In most cases, their application was limited only to promoter regions due to relatively good mapping of the latter in genome scale [22]; [23]; [24]. There are also resources, predicting GWAS SNPs effects on affinity of putative TFBS motifs found in known regulatory regions [51]; [52]. However, the genome-scale application of computational methods is usually complicated by the absence of direct experimental verification, making it difficult to set a threshold to avoid recognition of too many false-positive TFBSs as well as to considerably reduce the level of under-predictions [53].

Recently, high-throughput functional assays such as ChIP-seq and DNaseI-hypersensitivite sites identification by sequencing (DNase-seq) have emerged as promising approaches for genome-wide determination of regulatory units, promoting extensive data accumulation and appearance of such large-scale projects as the ENCODE. Several studies showed that in certain cases SNPs falling into ChIP-seq/DNase-seq regions directly affected binding of various TFs [54]; [55]; [56]. In a few pioneer works the genome wide map of regulatory units was produced by a combination of data from histone modification profiling, DNase-seq assay, and ChIP-seq experiments for individual TFs. Thus, a study of nine chromatin marks across nine cell types allowed to detect regulatory regions and found that disease-associated SNPs are significantly more likely to coincide with these regions (2-fold enrichment for cell type specific enhancers was shown) [57]. The 1.12 -fold enrichment for DNase I peaks and 1.25 enrichment for ChIP-seq peaks of certain TFs with such SNPs were demonstrated also [58]. Under identification of SNPs in ChIP-seq peaks for a multifunctional transcription and chromatin regulator CTCF the 2-fold enrichment for those associated with human diseases was observed [59].

Taking into account the fact that the gene regulatory regions in the majority of cases house multitude of sites for various TFs [60]; [61]; [62], we assumed that local clustering of ChIP-seq peaks for different TFs in a certain genomic region could suggest its regulatory significance. In this work, we have used the data obtained under the international ENCODE project on the distribution of binding sites for 134 TFs in the human genome (2.75 million ChIP-seq peaks). Our pipeline searches for the analyzed SNPs within genome regions where a number of TFs are bound. Using three samples of SNPs associated with phenotypic manifestations (S_clinic_ S_omim_ and S_gwas_) and the sample of random SNPs we have demonstrated that the higher the number of ChIP-seq peaks overlapping with SNPs, the larger is the enrichment of functionally significant samples for the putative rSNPs as compared with the random one (Fig. 2). Its maximal values reached 4.9- and 4.4-fold for S_omim_ and S_clinic_- correspondingly. We also found that filtering by OR and p-values is of importance when analysing S_gwas_ sample. Original S_gwas_ sample displayed only 2.6-3.3-fold enrichment with rSNPs relative to S_r_. But this value increased up to 4.9 when analyzing the subsample of S_gwas_, selected by more stringent statistical criteria on SNP-trait association (Fig. 3B). Thus, filtering by both OR and p-values produces a subset of high-confidence SNPs, comparable to S_omim_ and S_clinic_ samples in terms of functional relevance. These results suggest that a substantial portion of filtered out NHGRI GWAS SNPs do not cause any pronounced phenotypic manifestation, rather they are misidentified due to linkage with causal SNPs.

The sample of 40 clinically associated SNPs extracted from the OMIM catalog has been used to experimentally assess the efficiency of the proposed approach. As a result, 25 polymorphisms unambiguously behaved as regulatory when using the nuclear extract of at least one of the four cell lines, since they influenced the presence/absence of bands with different mobilitie in the binding pattern. In addition, 10 other SNPs led to changes only in the band intensities. Totally, 35 polymorphisms influence the binding of TFs with the genomic region where they are located, either destroying or creating binding sites; thus, 88% of the predicted rSNPs was experimentally confirmed to be regulatory polymorphisms.

Summing up, the current situation in the field of analysis of high-throughput sequencing data is such that the experimental technologies are considerably more advanced as compared with the bioinformatics tools for their support, analysis, and interpretation of experimental results. The proposed approach based on the experimental ChIP-seq data of the ENCODE project can be successfully used for a genome-wide identification of regulatory regions and rSNPs. Thus it will advantage for a considerable expansion of a number of SNPs able to influence gene expression as well as enhance detection of new markers for predisposition to various diseases, e.g. located within noncoding regions remote from the genes whose function they disrupt. This approach seems very promising to select potential rSNP sets from dbSNP, containing more than 45 billion entries, for further investigation of their clinical associations. In particular, it would be effective to reveal trait-associated SNPs from GWAS that are causal SNPs rather than tagging SNPs and guide new association studies.

## Supporting Information

Figure S1 **Bootstrapping procedure used in enrichment analysis.** In order to obtain the estimates of standard deviations and confidence intervals for the enrichment analysis, 500 random samples were generated by bootstrap resampling from each of the analyzed samples of both OTFR(*i*) and SNPs (e.g. Somim, Sclinic, Sgwas, Sr, etc.). The resulting samples were the same size as the initial sample, but some elements were selected repeatedly while some were excluded by chance. Pearson's chi-squared test (χ2) was used to test the hypothesis of normal distribution of the data. The enrichment E(*i*) was calculated for each pair of resulting SNP and OTFR(*i*) samples.
(TIF)

Figure S2 **Proportion of the genome falling into OTFRs, depending on the i values.** Total length of OTFRs consisting of at least *i* ChIP-seq peaks was calculated as percent of the genome length.
(TIF)

Table S1 **List of oligonucleotide probes tested in EMSA.**
(DOC)

Table S2 **Total length of OTFRs and proportion of the genome falling into OTFRs depending on the** ***i*** **value.**
(DOC)

Table S3 **The amount of SNPs falling into OTFRs with at least i TF binding loci, and significance of the corresponding enrichment (mean, SD - standard deviation, CI - confidence interval, and p-value of E(i)), for seven SNP samples analyzed.** Sample size is specified in brackets after sample name.
(DOC)

Table S4 **The SNPs and corresponding appearing or disrupted TF binding sites, revealed using TFBS profiles from JASPAR database.** Relative profile score threshold 0.8.
(DOC)

Document S1 **Data infile “TxN.txt” for creation MySQL table TxN (see Document S2).**
(GZ) Click here for additional data file.

Document S2 **The algorithm for data management and analysis used in the study.**
(DOC)

Document S3 **Putative rSNPs selected for EMSA and their position within OTFRs with** ***i*** **≥7.**
(DOC)
