# Supplementary figures and images for: Detection of Regulatory SNPs in Human Genome Using ChIP-seq ENCODE Data
Source: PLoS One. 2013 Oct 29;8(10):e78833. doi: 10.1371/journal.pone.0078833 (PMC3812152; doi:10.1371/journal.pone.0078833)

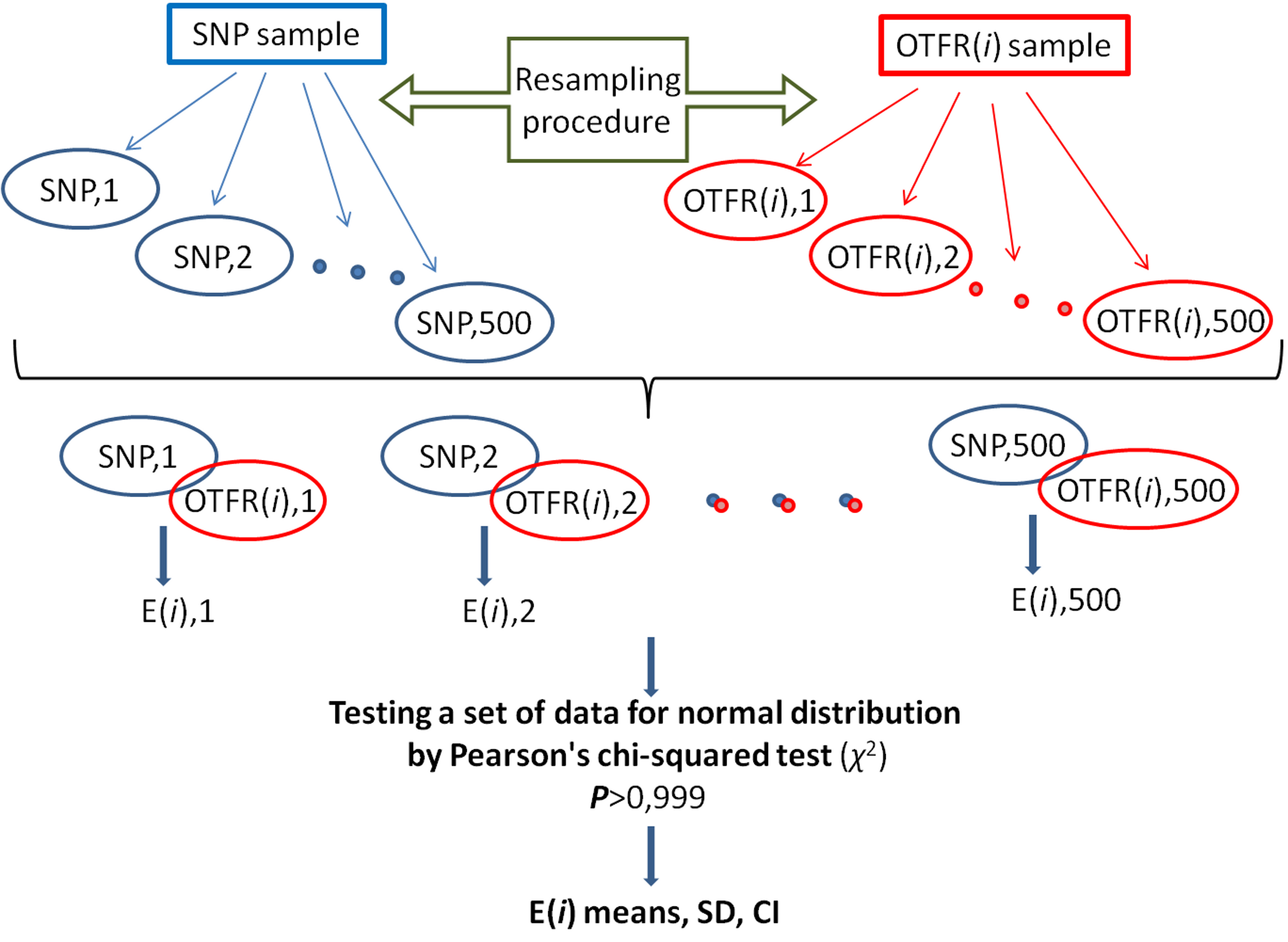

Supplement: Figure S1 — Bootstrapping procedure used in enrichment analysis. In order to obtain the estimates of standard deviations and confidence intervals for the enrichment analysis, 500 random samples were generated by bootstrap resampling from each of the analyzed samples of both OTFR(i) and SNPs (e.g. Somim, Sclinic, Sgwas, Sr, etc.). The resulting samples were the same size as the initial sample, but some elements were selected repeatedly while some were excluded by chance. Pearson's chi-squared test (χ2) was used to test the hypothesis of normal distribution of the data. The enrichment E(i) was calculated for each pair of resulting SNP and OTFR(i) samples. (TIF) [file pone.0078833.s001.tif]

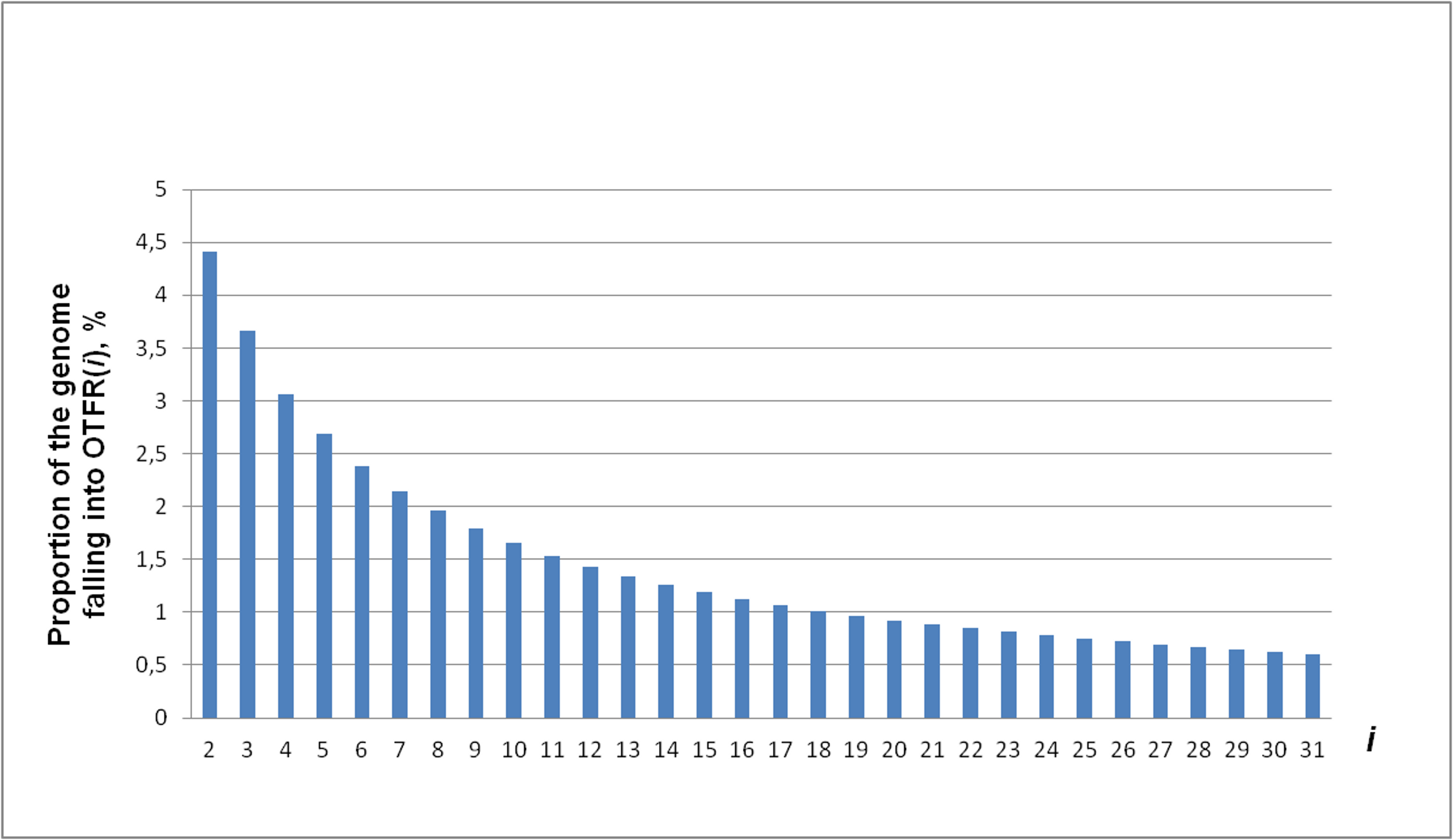

Supplement: Figure S2 — Proportion of the genome falling into OTFRs, depending on the i values. Total length of OTFRs consisting of at least i ChIP-seq peaks was calculated as percent of the genome length. (TIF) [file pone.0078833.s002.tif]
