# Supplementary material for: Detection of Regulatory SNPs in Human Genome Using ChIP-seq ENCODE Data
Source: PLoS One. 2013 Oct 29;8(10):e78833. doi: 10.1371/journal.pone.0078833 (PMC3812152; doi:10.1371/journal.pone.0078833)
Supplement: Table S1 — List of oligonucleotide probes tested in EMSA. (DOC) [file pone.0078833.s003.doc]

**Table S1.** List of oligonucleotide probes tested in EMSA.

| SNP identifier  in dbSNP NCBI | Gene name and SNP location in the gene | Sequences of oligonucleotides 5’-> 3’ |
| --- | --- | --- |
| rs10411210:C>T | RHPN2 (intron 2) | V1: cagtTGCCCTATTTGTTTT**C**GGGAAACCGTTGGTG |
|  |  | V2: cagtTGCCCTATTTGTTTT**T**GGGAAACCGTTGGTG |
| rs1048990:C>G | PSMA6 (5’-UTR) | V1: cagtTTTAAAGTAGTGCTT**C**TACCAACATGTCCCG |
|  |  | V2: cagtTTTAAAGTAGTGCTT**G**TACCAACATGTCCCG |
| rs11178998:A>G | TPH2 (5’-UTR) | V1: cagtTCCGCCAGCGCTGCT**A**CTGCCCCTCTAGTAC |
|  |  | V2: cagtTCCGCCAGCGCTGCT**G**CTGCCCCTCTAGTAC |
| rs113994210:C>G | CTNS (intron 10) | V1: cagtTCCGTCTGTCTGGCC**C**AGGCCTACATGAACT |
|  |  | V2: cagtTCCGTCTGTCTGGCC**G**AGGCCTACATGAACT |
| rs11466315:C>G | TGFB1 (5’-UTR) | V1: cagtCCCACCTCCCTCCGC**C**GAGCAGCCAGACAGC |
|  |  | V2: cagtCCCACCTCCCTCCGC**G**GAGCAGCCAGACAGC |
| rs12044852:C>A | CD58 (intron1) | V1: cagtCGTGATTCCTAACAG**C**TAGAATGAGGAGGGC |
|  |  | V2: cagtCGTGATTCCTAACAG**A**TAGAATGAGGAGGGC |
| rs12740374:G>T | CELSR2 (3’-UTR) | V1: cagtTCGGCTGCCCTGAGG**G**TGCTCAATCAAGCAC |
|  |  | V2: cagtTCGGCTGCCCTGAGG**T**TGCTCAATCAAGCAC |
| rs12885713:C>T | CALM1 (5’-UTR) | V1: cagtATATATATCGCGGGG**C**GCAGACTCGCGCTCC |
|  |  | V2: cagtATATATATCGCGGGG**T**GCAGACTCGCGCTCC |
| rs1532624:G>T | CETP (intron 7) | V1: cagtCCACACAGCTTGTGA**G**GCTGCAGCCCAAAGG |
|  |  | V2: cagtCCACACAGCTTGTGA**T**GCTGCAGCCCAAAGG |
| rs17039192:C>T | EPAS1 (5’-UTR) | V1: cagtCACACGGGTCCGGTG**C**CCGCTGCGCTTCCGC |
|  |  | V2: cagtCACACGGGTCCGGTG**T**CCGCTGCGCTTCCGC |
| rs1800734:A>G | EPM2AIP1 (promoter), MLH1 (5’-UTR) | V1: cagtGCGTAAGCTACAGCT**A**AAGGAAGAACGTGAG |
|  |  | V2: cagtGCGTAAGCTACAGCT**G**AAGGAAGAACGTGAG |
| rs2010963:C>G | VEGFA (5’-UTR) | V1: cagtTGCGAGCAGCGAAAG**C**GACAGGGGCAAAGTG |
|  |  | V2: cagtTGCGAGCAGCGAAAG**G**GACAGGGGCAAAGTG |
| rs2038137:A>C | KIAA0319 (5’-UTR) | V1: cagtGTATCTACTTCCCAG**A**GCGCCTGGCCGAGAA |
|  |  | V2: cagtGTATCTACTTCCCAG**C**GCGCCTGGCCGAGAA |
| rs2071002:A>C | NQO2  (5’-UTR) | V1: cagtCGGCTCCTACTGGGG**A**GTGCGCTGGTCGGAA |
|  |  | V2: cagtCGGCTCCTACTGGGG**C**GTGCGCTGGTCGGAA |
| rs2279744:G>T | MDM2 (intron 1) | V1: cagtGGCTGCGGGGCCGCT**G**CGGCGCGGGAGGTCC |
|  |  | V2: cagtGGCTGCGGGGCCGCT**T**CGGCGCGGGAGGTCC |
| rs2282978:C>T | CDK6 (intron 5) | V1: cagtTTGCATTGGATCATC**C**TTGGGAAACCACCTA |
|  |  | V2: cagtTTGCATTGGATCATC**T**TTGGGAAACCACCTA |
| rs2297339:A>G | HBS1L (5’-UTR) | V1: cagtCGCGACGTCTTAGCT**A**TGCACCGCGCGACGG |
|  |  | V2: cagtCGCGACGTCTTAGCT**G**TGCACCGCGCGACGG |
| rs3807306:A>C | IRF5 (intron 2) | V1: cagtCGAAAGTGGCTAGAC**A**GGGACAACTCAGAAA |
|  |  | V2: cagtCGAAAGTGGCTAGAC**C**GGGACAACTCAGAAA |
| rs4809324:C>T | RTEL1 (intron 16) | V1: cagtGGGCCTGGAATCTGT**C**TGTTCCATTGACCTC |
|  |  | V2: cagtGGGCCTGGAATCTGT**T**TGTTCCATTGACCTC |
| rs4821544:T>C | NCF4 (intron 1) | V1: cagtGCACGCAAACTCGAA**T**CTTCCGGAAGCAGCA |
|  |  | V2: cagtGCACGCAAACTCGAA**C**CTTCCGGAAGCAGCA |
| rs55853698:T>G | CHRNA5 (5’-UTR) | V1: cagtGCGCGGAGCGGCCCC**T**CTGCTGCGTCTGCCC |
|  |  | V2: cagtGCGCGGAGCGGCCCC**G**CTGCTGCGTCTGCCC |
| rs6958571:A>C | NOD1 (intron 9) | V1: cagtCAAGGCCCGCCCCCC**A**CACACACAGCAGGTT |
|  |  | V2: cagtCAAGGCCCGCCCCCC**C**CACACACAGCAGGTT |
| rs737865:C>T | COMT (intron 1), TXNRD2 (promoter) | V1: cagtAACAGGACACAAAAA**C**CCCTGGCTGGAAAAA |
|  |  | V2: cagtAACAGGACACAAAAA**T**CCCTGGCTGGAAAAA |
| rs74393987:C>T | APC (intron 2) | V1: cagtGAGGCAGGGGCGTCG**C**CCCCCCGCCCCCCAC |
|  |  | V2: cagtGAGGCAGGGGCGTCG**T**CCCCCCGCCCCCCAC |
| rs75612255:C>T | APC (intron 1) | V1: cagtATTTATTACTCTCCC**C**CCCACCTCCGGCATC |
|  |  | V2: cagtATTTATTACTCTCCC**T**CCCACCTCCGGCATC |
| rs75996864:G>T | APC (5’-UTR) | V1: cagtACCGACATGTGGCTG**G**ATTGGTGCAGCCCGC |
|  |  | V2: cagtACCGACATGTGGCTG**T**ATTGGTGCAGCCCGC |
| rs76241113:A>G | APC (intron 2) | V1: cagtGCGGGGGGAGGGGGG**A**AGGTGGTTTTCCCTC |
|  |  | V2: cagtGCGGGGGGAGGGGGG**G**AGGTGGTTTTCCCTC |
| rs77733015:G>T | APC (intron 1) | V1: cagtGGGCTAGGCAGGCTG**G**GCGGTTGGGCGGGGC |
|  |  | V2: cagtGGGCTAGGCAGGCTG**T**GCGGTTGGGCGGGGC |
| rs78037487:G>C | APC (intron 1) | V1: cagtCCATTCCCGTCGGGA**G**CCCGCCGATTGGCTG |
|  |  | V2: cagtCCATTCCCGTCGGGA**C**CCCGCCGATTGGCTG |
| rs78597499:G>T | APC (intron 1) | V1: cagtAGGCAGGCTGTGCGG**G**TGGGCGGGGCCCTGT |
|  |  | V2: cagtAGGCAGGCTGTGCGG**T**TGGGCGGGGCCCTGT |
| rs79216719:A>C | APC (intron 1) | V1: cagtCCTTCTGCCCTGCGG**A**CCTCCCCCGACTCTT |
|  |  | V2: cagtCCTTCTGCCCTGCGG**C**CCTCCCCCGACTCTT |
| rs79488395:A>C | APC (intron 1) | V1: cagtCTTTACTATGCGTGT**A**AACTGCCATCAACTT |
|  |  | V2: cagtCTTTACTATGCGTGT**C**AACTGCCATCAACTT |
| rs79577178:C>G | APC (intron 2) | V1: cagtGGGCGTCGTCCCCCC**C**CCCCCCACTGCAGCA |
|  |  | V2: cagtGGGCGTCGTCCCCCC**G**CCCCCCACTGCAGCA |
| rs7961894:C>T | WDR66 (intron 3) | V1: cagtGCTGTCACACAAGGT**C**CTACAGTGAACGTGC |
|  |  | V2: cagtGCTGTCACACAAGGT**T**CTACAGTGAACGTGC |
| rs79734816:C>T | APC (intron 1) | V1: cagtTCTGCCCTGCGGACC**C**CCCCCGACTCTTTAC |
|  |  | V2: cagtTCTGCCCTGCGGACC**T**CCCCCGACTCTTTAC |
| rs80112297:A>G | APC (intron 2) | V1: cagtGCTTGCTGCGGGGGG**A**GGGGGGAAGGTGGTT |
|  |  | V2: cagtGCTTGCTGCGGGGGG**G**GGGGGGAAGGTGGTT |
| rs80313086:G>T | APC (intron 2) | V1: cagtGGGGAGGGGGGAAGG**G**GGTTTTCCCTCGCAC |
|  |  | V2: cagtGGGGAGGGGGGAAGG**T**GGTTTTCCCTCGCAC |
| rs9465871:T>C | CDKAL1 (intron 5) | V1: cagtAGTGTTGCTGAGAAA**T**TGAGTTAGATGAAGA |
|  |  | V2: cagtAGTGTTGCTGAGAAA**C**TGAGTTAGATGAAGA |
| rs3057:A>G | ASAP1  (intron 4) | V1: cagtCTGATGGAAGCATCA**A**TGATGGATTTGGCTT |
|  |  | V2: cagtCTGATGGAAGCATCA**G**TGATGGATTTGGCTT |
| rs3766379:C>T | [CD244](http://www.ncbi.nlm.nih.gov/entrez/query.fcgi?db=gene&cmd=Retrieve&dopt=Graphics&list_uids=51744)  (intron 5) | V1: cagtGCTCCTGGAGGAGCC**C**ACTCAGGCTGGTGGG |
|  |  | V2: cagtGCTCCTGGAGGAGCC**T**ACTCAGGCTGGTGGG |

Two allelic versions of oligonucleotides correspond to each SNP. SNP position marked in bold style. Tetranucleotide overhangs are indicated in lowercase letters.
