# Supplementary material for: Detection of Regulatory SNPs in Human Genome Using ChIP-seq ENCODE Data
Source: PLoS One. 2013 Oct 29;8(10):e78833. doi: 10.1371/journal.pone.0078833 (PMC3812152; doi:10.1371/journal.pone.0078833)
Supplement: Table S2 — Total length of OTFRs and proportion of the genome falling into OTFRs depending on the i value. (DOC) [file pone.0078833.s004.doc]

| ***i*** | Total length of OTFR(*i*), nt | Proportion of the genome falling into OTFR(*i*), % | ***i*** | Total length of OTFR(*i*), nt | Proportion of the genome falling into OTFR(*i*), % |
| --- | --- | --- | --- | --- | --- |
| 2 | 132280147 | 4,4 | 17 | 32042136 | 1,1 |
| 3 | 110039199 | 3,7 | 18 | 30461980 | 1,0 |
| 4 | 91816088 | 3,1 | 19 | 29014370 | 1,0 |
| 5 | 80518050 | 2,7 | 20 | 27707254 | 0,9 |
| 6 | 71530999 | 2,4 | 21 | 26586895 | 0,9 |
| 7 | 64451609 | 2,1 | 22 | 25493517 | 0,8 |
| 8 | 58717381 | 2,0 | 23 | 24421307 | 0,8 |
| 9 | 53821973 | 1,8 | 24 | 23473109 | 0,8 |
| 10 | 49678660 | 1,7 | 25 | 22583044 | 0,8 |
| 11 | 46082184 | 1,5 | 26 | 21704011 | 0,7 |
| 12 | 42969174 | 1,4 | 27 | 20922602 | 0,7 |
| 13 | 40247184 | 1,3 | 28 | 20161127 | 0,7 |
| 14 | 37875356 | 1,3 | 29 | 19445560 | 0,6 |
| 15 | 35705945 | 1,2 | 30 | 18801110 | 0,6 |
| 16 | 33799977 | 1,1 | 31 | 18164705 | 0,6 |

**Table S2.** Total length of OTFRs and proportion of the genome falling into OTFRs depending on the *i* value.
