# Supplementary material for: Detection of Regulatory SNPs in Human Genome Using ChIP-seq ENCODE Data
Source: PLoS One. 2013 Oct 29;8(10):e78833. doi: 10.1371/journal.pone.0078833 (PMC3812152; doi:10.1371/journal.pone.0078833)
Supplement: Table S3 — The amount of SNPs falling into OTFRs with at least i TF binding loci, and significance of the corresponding enrichment (mean, SD - standard deviation, CI - confidence interval, and p-value of E(i)), for seven SNP samples analyzed. Sample size is specified in brackets after sample name. (DOC) [file pone.0078833.s005.doc]

**Table S3.** The amount of SNPs falling into OTFRs with at least *i* TF binding loci, and significance of the corresponding enrichment (mean, SD - standard deviation, CI - confidence interval, and p-value of E(*i*)), for seven SNP samples analyzed. Sample size is specified in brackets after sample name.

| **S_r_ (1 000 000)** | | | | | | |
| --- | --- | --- | --- | --- | --- | --- |
| ***i*** | SNPs | SNPs,% | Mean | SD | CI | *p*-value |
| 2 | 44887 | 4.5 | 0.996 | 0.009 | 0.001 | 9,05X10^-8^ |
| 3 | 36789 | 3.7 | 0.986 | 0.010 | 0.001 | 0,419755 |
| 4 | 30345 | 3.0 | 0.971 | 0.011 | 0.001 | 0,035315 |
| 5 | 26175 | 2.6 | 0.961 | 0.012 | 0.001 | 9,76X10^-9^ |
| 6 | 23014 | 2.3 | 0.951 | 0.012 | 0.001 | 2,99X10^-14^ |
| 7 | 20553 | 2.1 | 0.940 | 0.013 | 0.001 | 2,67X10^-19^ |
| 8 | 18515 | 1.9 | 0.930 | 0.013 | 0.002 | 1,14X10^-26^ |
| 9 | 16787 | 1.7 | 0.928 | 0.014 | 0.002 | 3,94X10^-34^ |
| 10 | 15442 | 1.5 | 0.918 | 0.014 | 0.002 | 1,14X10^-34^ |
| 11 | 14164 | 1.4 | 0.912 | 0.015 | 0.002 | 1,87X10^-42^ |
| 12 | 13119 | 1.3 | 0.907 | 0.015 | 0.002 | 6,14X10^-46^ |
| 13 | 12214 | 1.2 | 0.894 | 0.015 | 0.002 | 9,62X10^-49^ |
| 14 | 11332 | 1.1 | 0.890 | 0.016 | 0.002 | 1,47X10^-59^ |
| 15 | 10641 | 1.1 | 0.883 | 0.016 | 0.002 | 4,64X10^-60^ |
| 16 | 9994 | 1.0 | 0.877 | 0.017 | 0.002 | 1,73X10^-64^ |
| 17 | 9405 | 0.9 | 0.870 | 0.017 | 0.002 | 3,05X10^-68^ |
| 18 | 8878 | 0.9 | 0.870 | 0.018 | 0.002 | 1,02X10^-71^ |
| 19 | 8442 | 0.8 | 0.863 | 0.018 | 0.002 | 5,98X10^-70^ |
| 20 | 8004 | 0.8 | 0.858 | 0.018 | 0.002 | 1,99X10^-73^ |
| 21 | 7627 | 0.8 | 0.854 | 0.018 | 0.002 | 7,13X10^-77^ |
| 22 | 7288 | 0.7 | 0.849 | 0.018 | 0.002 | 6,71X10^-77^ |
| 23 | 6942 | 0.7 | 0.846 | 0.019 | 0.002 | 1,01X10^-78^ |
| 24 | 6652 | 0.7 | 0.845 | 0.019 | 0.002 | 2,18X10^-78^ |
| 25 | 6396 | 0.6 | 0.843 | 0.019 | 0.002 | 5,59X10^-76^ |
| 26 | 6128 | 0.6 | 0.837 | 0.019 | 0.002 | 1,31X10^-75^ |
| 27 | 5869 | 0.6 | 0.832 | 0.019 | 0.002 | 3,68X10^-78^ |
| 28 | 5616 | 0.6 | 0.827 | 0.020 | 0.002 | 6,35X10^-81^ |
| 29 | 5389 | 0.5 | 0.825 | 0.020 | 0.002 | 3,94X10^-82^ |
| 30 | 5198 | 0.5 | 0.819 | 0.020 | 0.002 | 2,65X10^-81^ |
| 31 | 4988 | 0.5 | 0.814 | 0.020 | 0.002 | 9,31X10^-84^ |
| **S_clinic_ (34 373)** | | | | | | |
| ***i*** | SNPs | SNPs,% | Mean | SD | CI | *p*-value |
| 2 | 3007 | 8.7 | 1.990 | 0.144 | 0.017 | <10^-290^ |
| 3 | 2537 | 7.4 | 2.022 | 0.165 | 0.019 | <10^-290^ |
| 4 | 2190 | 6.4 | 2.077 | 0.154 | 0.018 | <10^-290^ |
| 5 | 2045 | 5.9 | 2.211 | 0.172 | 0.020 | <10^-290^ |
| 6 | 1855 | 5.4 | 2.260 | 0.187 | 0.022 | <10^-290^ |
| 7 | 1784 | 5.2 | 2.411 | 0.207 | 0.024 | <10^-290^ |
| 8 | 1672 | 4.9 | 2.488 | 0.224 | 0.026 | <10^-290^ |
| 9 | 1550 | 4.5 | 2.495 | 0.230 | 0.026 | <10^-290^ |
| 10 | 1493 | 4.3 | 2.599 | 0.247 | 0.028 | <10^-290^ |
| 11 | 1388 | 4.0 | 2.648 | 0.244 | 0.028 | <10^-290^ |
| 12 | 1304 | 3.8 | 2.664 | 0.263 | 0.030 | <10^-290^ |
| 13 | 1270 | 3.7 | 2.778 | 0.278 | 0.032 | <10^-290^ |
| 14 | 1219 | 3.5 | 2.832 | 0.292 | 0.034 | <10^-290^ |
| 15 | 1151 | 3.3 | 2.846 | 0.301 | 0.035 | <10^-290^ |
| 16 | 1118 | 3.3 | 2.924 | 0.314 | 0.036 | <10^-290^ |
| 17 | 1043 | 3.0 | 2.885 | 0.327 | 0.038 | <10^-290^ |
| 18 | 1019 | 3.0 | 2.967 | 0.338 | 0.039 | <10^-290^ |
| 19 | 979 | 2.8 | 2.998 | 0.354 | 0.041 | <10^-290^ |
| 20 | 965 | 2.8 | 3.093 | 0.370 | 0.043 | <10^-290^ |
| 21 | 926 | 2.7 | 3.097 | 0.375 | 0.043 | <10^-290^ |
| 22 | 914 | 2.7 | 3.183 | 0.390 | 0.045 | <10^-290^ |
| 23 | 893 | 2.6 | 3.249 | 0.408 | 0.047 | <10^-290^ |
| 24 | 873 | 2.5 | 3.307 | 0.420 | 0.048 | <10^-290^ |
| 25 | 841 | 2.4 | 3.313 | 0.433 | 0.050 | <10^-290^ |
| 26 | 822 | 2.4 | 3.371 | 0.448 | 0.052 | <10^-290^ |
| 27 | 797 | 2.3 | 3.404 | 0.461 | 0.053 | <10^-290^ |
| 28 | 786 | 2.3 | 3.480 | 0.477 | 0.055 | <10^-290^ |
| 29 | 771 | 2.2 | 3.540 | 0.491 | 0.057 | <10^-290^ |
| 30 | 761 | 2.2 | 3.618 | 0.510 | 0.059 | <10^-290^ |
| 31 | 744 | 2.2 | 3.659 | 0.524 | 0.060 | <10^-290^ |
| **S_omim_ (18 291)** | | | | | | |
| ***i*** | SNPs | SNPs,% | Mean | SD | CI | *p*-value |
| 2 | 1917 | 10.5 | 2.362 | 0.214 | 0.025 | <10^-290^ |
| 3 | 1619 | 8.9 | 2.379 | 0.193 | 0.022 | <10^-290^ |
| 4 | 1352 | 7.4 | 2.562 | 0.219 | 0.025 | <10^-290^ |
| 5 | 1276 | 7.0 | 2.706 | 0.239 | 0.028 | <10^-290^ |
| 6 | 1196 | 6.5 | 2.864 | 0.261 | 0.030 | <10^-290^ |
| 7 | 1140 | 6.2 | 2.907 | 0.279 | 0.032 | <10^-290^ |
| 8 | 1055 | 5.8 | 2.902 | 0.274 | 0.032 | <10^-290^ |
| 9 | 967 | 5.3 | 3.002 | 0.298 | 0.034 | <10^-290^ |
| 10 | 920 | 5.0 | 3.042 | 0.293 | 0.034 | <10^-290^ |
| 11 | 861 | 4.7 | 3.103 | 0.309 | 0.036 | <10^-290^ |
| 12 | 819 | 4.5 | 3.188 | 0.325 | 0.037 | <10^-290^ |
| 13 | 790 | 4.3 | 3.235 | 0.338 | 0.039 | <10^-290^ |
| 14 | 752 | 4.1 | 3.292 | 0.352 | 0.041 | <10^-290^ |
| 15 | 722 | 3.9 | 3.402 | 0.369 | 0.042 | <10^-290^ |
| 16 | 706 | 3.9 | 3.295 | 0.362 | 0.042 | <10^-290^ |
| 17 | 647 | 3.5 | 3.396 | 0.382 | 0.044 | <10^-290^ |
| 18 | 634 | 3.5 | 3.446 | 0.395 | 0.046 | <10^-290^ |
| 19 | 610 | 3.3 | 3.536 | 0.408 | 0.047 | <10^-290^ |
| 20 | 598 | 3.3 | 3.520 | 0.419 | 0.048 | <10^-290^ |
| 21 | 571 | 3.1 | 3.612 | 0.433 | 0.050 | <10^-290^ |
| 22 | 561 | 3.1 | 3.683 | 0.452 | 0.052 | <10^-290^ |
| 23 | 548 | 3.0 | 3.745 | 0.468 | 0.054 | <10^-290^ |
| 24 | 537 | 2.9 | 3.738 | 0.475 | 0.055 | <10^-290^ |
| 25 | 517 | 2.8 | 3.751 | 0.491 | 0.057 | <10^-290^ |
| 26 | 499 | 2.7 | 3.740 | 0.496 | 0.057 | <10^-290^ |
| 27 | 482 | 2.6 | 3.814 | 0.514 | 0.059 | <10^-290^ |
| 28 | 473 | 2.6 | 3.859 | 0.532 | 0.061 | <10^-290^ |
| 29 | 460 | 2.5 | 3.950 | 0.548 | 0.063 | <10^-290^ |
| 30 | 455 | 2.5 | 3.965 | 0.560 | 0.064 | <10^-290^ |
| 31 | 440 | 2.4 | 3.975 | 0.564 | 0.065 | <10^-290^ |
| **S_gwas_ (10 345)** | | | | | | |
| ***i*** | SNPs | SNPs,% | Mean | SD | CI | *p*-value |
| 2 | 735 | 7.1 | 2.003 | 0.131 | 0.015 | 3,98X10^-76^ |
| 3 | 652 | 6.3 | 2.137 | 0.150 | 0.017 | 3,85X10^-87^ |
| 4 | 565 | 5.5 | 2.222 | 0.173 | 0.020 | 9,49X10^-87^ |
| 5 | 495 | 4.8 | 2.220 | 0.183 | 0.021 | 5,55X10^-76^ |
| 6 | 445 | 4.3 | 2.245 | 0.192 | 0.022 | 2,44X10^-71^ |
| 7 | 408 | 3.9 | 2.288 | 0.203 | 0.023 | 1,71X10^-69^ |
| 8 | 380 | 3.7 | 2.342 | 0.214 | 0.025 | 1,14X10^-69^ |
| 9 | 353 | 3.4 | 2.378 | 0.229 | 0.026 | 1,21X10^-67^ |
| 10 | 326 | 3.2 | 2.379 | 0.236 | 0.027 | 1,03X10^-62^ |
| 11 | 299 | 2.9 | 2.358 | 0.241 | 0.028 | 1,16X10^-55^ |
| 12 | 279 | 2.7 | 2.370 | 0.250 | 0.029 | 3,56X10^-52^ |
| 13 | 267 | 2.6 | 2.424 | 0.263 | 0.030 | 1,87X10^-53^ |
| 14 | 248 | 2.4 | 2.399 | 0.271 | 0.031 | 8,18X10^-48^ |
| 15 | 233 | 2.3 | 2.392 | 0.278 | 0.032 | 1,48X10^-44^ |
| 16 | 221 | 2.1 | 2.391 | 0.286 | 0.033 | 1,3X10^-42^ |
| 17 | 209 | 2.0 | 2.387 | 0.290 | 0.033 | 4,32X10^-40^ |
| 18 | 201 | 1.9 | 2.408 | 0.295 | 0.034 | 5,11X10^-40^ |
| 19 | 193 | 1.9 | 2.432 | 0.305 | 0.035 | 1,9X10^-39^ |
| 20 | 183 | 1.8 | 2.417 | 0.310 | 0.036 | 1,07X10^-36^ |
| 21 | 172 | 1.7 | 2.377 | 0.316 | 0.036 | 1,84X10^-32^ |
| 22 | 166 | 1.6 | 2.389 | 0.322 | 0.037 | 5,04X10^-32^ |
| 23 | 158 | 1.5 | 2.379 | 0.328 | 0.038 | 5,82X10^-30^ |
| 24 | 149 | 1.4 | 2.352 | 0.334 | 0.038 | 1,04X10^-26^ |
| 25 | 139 | 1.3 | 2.288 | 0.338 | 0.039 | 1,17X10^-22^ |
| 26 | 131 | 1.3 | 2.241 | 0.352 | 0.041 | 4,31X10^-20^ |
| 27 | 124 | 1.2 | 2.197 | 0.352 | 0.041 | 5,97X10^-18^ |
| 28 | 118 | 1.1 | 2.175 | 0.355 | 0.041 | 2X10^-16^ |
| 29 | 114 | 1.1 | 2.177 | 0.358 | 0.041 | 5,16X10^-16^ |
| 30 | 109 | 1.1 | 2.149 | 0.359 | 0.041 | 8,66X10^-15^ |
| 31 | 104 | 1.0 | 2.116 | 0.364 | 0.042 | 1,46X10^-13^ |
| **S_OR_ (3 084)** | | | | | | |
| ***i*** | SNPs | SNPs,% | Mean | SD | CI | *p*-value |
| 2 | 329 | 10.7 | 2.366 | 0.265 | 0.031 | 3,5X10^-121^ |
| 3 | 297 | 9.6 | 2.565 | 0.303 | 0.035 | 5X10^-132^ |
| 4 | 259 | 8.4 | 2.670 | 0.341 | 0.039 | 6,9X10^-127^ |
| 5 | 237 | 7.7 | 2.787 | 0.377 | 0.043 | 5,2X10^-127^ |
| 6 | 217 | 7.0 | 2.876 | 0.417 | 0.048 | 9,2X10^-124^ |
| 7 | 207 | 6.7 | 3.041 | 0.456 | 0.053 | 4,7X10^-132^ |
| 8 | 189 | 6.1 | 3.076 | 0.489 | 0.056 | 3X10^-121^ |
| 9 | 162 | 5.3 | 2.887 | 0.487 | 0.056 | 1,9X10^-91^ |
| 10 | 152 | 4.9 | 2.926 | 0.520 | 0.060 | 9,36X10^-89^ |
| 11 | 137 | 4.4 | 2.843 | 0.548 | 0.063 | 9,79X10^-76^ |
| 12 | 131 | 4.2 | 2.915 | 0.582 | 0.067 | 3,25X10^-76^ |
| 13 | 126 | 4.1 | 2.988 | 0.614 | 0.071 | 2,87X10^-77^ |
| 14 | 117 | 3.8 | 2.934 | 0.629 | 0.072 | 4,78X10^-70^ |
| 15 | 107 | 3.5 | 2.837 | 0.637 | 0.073 | 1,66X10^-60^ |
| 16 | 100 | 3.2 | 2.802 | 0.666 | 0.077 | 3,05X10^-55^ |
| 17 | 100 | 3.2 | 2.956 | 0.703 | 0.081 | 2,45X10^-61^ |
| 18 | 93 | 3.0 | 2.868 | 0.710 | 0.082 | 8,64X10^-55^ |
| 19 | 90 | 2.9 | 2.918 | 0.737 | 0.085 | 9,69X10^-55^ |
| 20 | 88 | 2.9 | 2.987 | 0.755 | 0.087 | 4,92X10^-56^ |
| 21 | 86 | 2.8 | 3.052 | 0.772 | 0.089 | 1,01X10^-56^ |
| 22 | 82 | 2.7 | 3.034 | 0.794 | 0.091 | 1,34X10^-53^ |
| 23 | 78 | 2.5 | 3.012 | 0.805 | 0.093 | 2,12X10^-50^ |
| 24 | 76 | 2.5 | 3.059 | 0.825 | 0.095 | 2,01X10^-50^ |
| 25 | 72 | 2.3 | 3.004 | 0.842 | 0.097 | 1,67X10^-46^ |
| 26 | 70 | 2.3 | 3.028 | 0.865 | 0.100 | 3,01X10^-46^ |
| 27 | 64 | 2.1 | 2.908 | 0.876 | 0.101 | 2,14X10^-38^ |
| 28 | 62 | 2.0 | 2.908 | 0.894 | 0.103 | 1,24X10^-37^ |
| 29 | 60 | 1.9 | 2.901 | 0.911 | 0.105 | 1,04X10^-36^ |
| 30 | 60 | 1.9 | 3.001 | 0.942 | 0.109 | 4,09X10^-39^ |
| 31 | 60 | 1.9 | 3.107 | 0.975 | 0.112 | 1,11X10^-41^ |
| **S_pV_ (5 115)** | | | | | | |
| ***i*** | SNPs | SNPs,% | Mean | SD | CI | *p*-value |
| 2 | 630 | 12.3 | 2.776 | 0.251 | 0.029 | <10^-290^ |
| 3 | 587 | 11.5 | 3.118 | 0.294 | 0.034 | <10^-290^ |
| 4 | 523 | 10.2 | 3.314 | 0.335 | 0.039 | <10^-290^ |
| 5 | 465 | 9.1 | 3.362 | 0.359 | 0.041 | <10^-290^ |
| 6 | 429 | 8.4 | 3.483 | 0.393 | 0.045 | <10^-290^ |
| 7 | 395 | 7.7 | 3.548 | 0.410 | 0.047 | <10^-290^ |
| 8 | 358 | 7.0 | 3.546 | 0.410 | 0.047 | 6,9X10^-291^ |
| 9 | 324 | 6.3 | 3.516 | 0.411 | 0.047 | 1,3X10^-257^ |
| 10 | 300 | 5.9 | 3.531 | 0.432 | 0.050 | 5,2X10^-240^ |
| 11 | 279 | 5.5 | 3.541 | 0.452 | 0.052 | 2,2X10^-224^ |
| 12 | 268 | 5.2 | 3.648 | 0.479 | 0.055 | 3,9X10^-227^ |
| 13 | 255 | 5.0 | 3.715 | 0.502 | 0.058 | 3,6X10^-222^ |
| 14 | 242 | 4.7 | 3.750 | 0.526 | 0.061 | 5,1X10^-214^ |
| 15 | 226 | 4.4 | 3.714 | 0.550 | 0.063 | 8,4X10^-197^ |
| 16 | 216 | 4.2 | 3.750 | 0.575 | 0.066 | 2,6X10^-191^ |
| 17 | 206 | 4.0 | 3.780 | 0.588 | 0.068 | 2X10^-184^ |
| 18 | 199 | 3.9 | 3.836 | 0.602 | 0.069 | 4X10^-183^ |
| 19 | 191 | 3.7 | 3.872 | 0.627 | 0.072 | 3,9X10^-178^ |
| 20 | 178 | 3.5 | 3.734 | 0.603 | 0.069 | 1,9X10^-159^ |
| 21 | 169 | 3.3 | 3.687 | 0.614 | 0.071 | 9,1X10^-149^ |
| 22 | 164 | 3.2 | 3.735 | 0.646 | 0.074 | 2,1X10^-147^ |
| 23 | 156 | 3.0 | 3.701 | 0.659 | 0.076 | 1,2X10^-138^ |
| 24 | 152 | 3.0 | 3.748 | 0.677 | 0.078 | 2,7X10^-138^ |
| 25 | 140 | 2.7 | 3.592 | 0.670 | 0.077 | 2,2X10^-118^ |
| 26 | 136 | 2.7 | 3.633 | 0.690 | 0.079 | 3,4X10^-117^ |
| 27 | 127 | 2.5 | 3.517 | 0.700 | 0.081 | 1,1X10^-103^ |
| 28 | 120 | 2.3 | 3.432 | 0.709 | 0.082 | 9,05X10^-95^ |
| 29 | 116 | 2.3 | 3.449 | 0.729 | 0.084 | 4,89X10^-92^ |
| 30 | 114 | 2.2 | 3.502 | 0.749 | 0.086 | 4,14X10^-93^ |
| 31 | 111 | 2.2 | 3.535 | 0.769 | 0.089 | 6,05X10^-92^ |
| **S_int_ (1 850)** | | | | | | |
| ***i*** | SNPs | SNPs,% | Mean | SD | CI | *p*-value |
| 2 | 235 | 12.7 | 2.366 | 0.265 | 0.031 | 1,6X10^-127^ |
| 3 | 219 | 11.8 | 2.565 | 0.303 | 0.035 | 1,9X10^-148^ |
| 4 | 190 | 10.3 | 2.670 | 0.341 | 0.039 | 1,1X10^-138^ |
| 5 | 179 | 9.7 | 2.787 | 0.377 | 0.043 | 1,4X10^-148^ |
| 6 | 163 | 8.8 | 2.876 | 0.417 | 0.048 | 2,2X10^-141^ |
| 7 | 158 | 8.5 | 3.041 | 0.456 | 0.053 | 4,7X10^-155^ |
| 8 | 140 | 7.6 | 3.076 | 0.489 | 0.056 | 2X10^-131^ |
| 9 | 119 | 6.4 | 2.887 | 0.487 | 0.056 | 1,69X10^-98^ |
| 10 | 112 | 6.1 | 2.926 | 0.520 | 0.060 | 5,4X10^-96^ |
| 11 | 103 | 5.6 | 2.843 | 0.548 | 0.063 | 3,92X10^-87^ |
| 12 | 99 | 5.4 | 2.915 | 0.582 | 0.067 | 2,8X10^-88^ |
| 13 | 96 | 5.2 | 2.988 | 0.614 | 0.071 | 8,63X10^-91^ |
| 14 | 90 | 4.9 | 2.934 | 0.629 | 0.072 | 1,06X10^-84^ |
| 15 | 82 | 4.4 | 2.837 | 0.637 | 0.073 | 4,8X10^-73^ |
| 16 | 77 | 4.2 | 2.802 | 0.666 | 0.077 | 8,95X10^-68^ |
| 17 | 77 | 4.2 | 2.956 | 0.703 | 0.081 | 4,22X10^-74^ |
| 18 | 71 | 3.8 | 2.868 | 0.710 | 0.082 | 4,33X10^-65^ |
| 19 | 69 | 3.7 | 2.918 | 0.737 | 0.085 | 1,85X10^-65^ |
| 20 | 67 | 3.6 | 2.987 | 0.755 | 0.087 | 2,2X10^-65^ |
| 21 | 65 | 3.5 | 3.052 | 0.772 | 0.089 | 1,24X10^-64^ |
| 22 | 62 | 3.4 | 3.034 | 0.794 | 0.091 | 3,29X10^-61^ |
| 23 | 58 | 3.1 | 3.012 | 0.805 | 0.093 | 3,41X10^-55^ |
| 24 | 58 | 3.1 | 3.059 | 0.825 | 0.095 | 7,13X10^-59^ |
| 25 | 54 | 2.9 | 3.004 | 0.842 | 0.097 | 4,34X10^-52^ |
| 26 | 53 | 2.9 | 3.028 | 0.865 | 0.100 | 6,16X10^-53^ |
| 27 | 48 | 2.6 | 2.908 | 0.876 | 0.101 | 1,97X10^-43^ |
| 28 | 47 | 2.5 | 2.908 | 0.894 | 0.103 | 1,04X10^-43^ |
| 29 | 46 | 2.5 | 2.901 | 0.911 | 0.105 | 7,4X10^-44^ |
| 30 | 46 | 2.5 | 3.001 | 0.942 | 0.109 | 2,53X10^-46^ |
| 31 | 46 | 2.5 | 3.107 | 0.975 | 0.112 | 6,08X10^-49^ |

**Supp. Table S3.** The E(*i*) means, standard deviation, confidence intervals as well as number and percent of SNPs falling into OTFR with at least *i* TF binding loci for samples analyzed.
