# Supplementary material for: Detection of Regulatory SNPs in Human Genome Using ChIP-seq ENCODE Data
Source: PLoS One. 2013 Oct 29;8(10):e78833. doi: 10.1371/journal.pone.0078833 (PMC3812152; doi:10.1371/journal.pone.0078833)
Supplement: Table S4 — The SNPs and corresponding appearing or disrupted TF binding sites, revealed using TFBS profiles from JASPAR database. Relative profile score threshold 0.8. (DOC) [file pone.0078833.s006.doc]

**Table S4.** The SNPs and corresponding appearing or disrupted TF binding sites, revealed using TFBS profiles from JASPAR database (Relative profile score threshold 0.8).

| SNP identifier in dbSNP NCBI | Appearing TFBS (relative score) | Disrupted TFBS (relative score) | Affected TFBS (initial and changed relative scores with difference >0.05) |
| --- | --- | --- | --- |
| rs10411210 |  | MA0027.1 En1 (0.82)  MA0031.1 FOXD1 (0.85)  MA0157.1 FOXO3 (0.84)  MA0024.1 E2F1 (0.80) | MA0144.1  Stat3 (0.87→ 0.81)  MA0081.1  SPIB (0.88→ 0.81) |
| rs1048990 | MA0102.2 CEBPA (0.81)  MA0145.1 Tcfcp2l1 (0.80)  MA0145.1 Tcfcp2l1 (0.80) |  | MA0158.1  HOXA5 (0.86→ 0.80)  MA0109.1  Hltf (0.82→ 0.90) |
| rs11178998 | MA0048.1 NHLH1 (0.86)  MA0048.1 NHLH1 (0.85) | MA0100.1 Myb (0.84) | MA0055.1  Myf (0.82→ 0.91) |
| rs11466315 |  |  |  |
| rs12044852 | MA0145.1 Tcfcp2l1 (0.80) | MA0035.2 Gata1 (0.89) | MA0259.1  HIF1A::ARNT (0.81→ 0.92) |
| rs12740374 | MA0100.1 Myb (0.80)  MA0043.1 HLF (0.84)  MA0102.2 CEBPA (0.90)  MA0145.1 Tcfcp2l1 (0.80)  MA0102.2 CEBPA (0.92)  MA0081.1 SPIB (0.81) | MA0027.1 En1 (0.82) |  |
| rs12885713 |  |  | MA0033.1  FOXL1 (0.92→ 0.82) |
| rs1532624 | MA0067.1 Pax2 (0.83)  MA0089.1  NFE2L1::MafG (0.80)  MA0150.1 NFE2L2 (0.80) | MA0146.1 Zfx (0.84) |  |
| rs17039192 |  | MA0161.1 NFIC (0.81) |  |
| rs1800734 | MA0055.1 Myf (0.88)  MA0055.1 Myf (0.80) | MA0035.2 Gata1 (0.81) | MA0117.1  Mafb (0.83→ 0.94)  MA0004.1  Arnt (0.93→ 0.83)  MA0003.1  TFAP2A (0.80→ 0.88) |
| rs2010963 | MA0079.2 SP1 (0.82)  MA0079.2 SP1 (0.83) | MA0062.2 GABPA (0.81)  MA0051.1 IRF2 (0.81)  MA0050.1 IRF1 (0.84) |  |
| rs2038137 |  |  |  |
| rs2071002 | MA0162.1 Egr1 (0.80)  MA0259.1 HIF1A::ARNT (0.95 | MA0139.1 CTCF (0.80)  MA0027.1 En1 (0.80)  MA0130.1  ZNF354C (0.81) | MA0056.1  MZF1_1-4 (0.99→ 0.81) |
| rs2279744 | MA0062.2 GABPA (0.82)  MA0024.1 E2F1 (0.87) | MA0048.1 NHLH1 (0.80)  MA0100.1 Myb (0.87) | MA0117.1  Mafb (0.81→ 0.88 |
| rs2282978 | MA0037.1 GATA3 (0.83) | MA0136.1 ELF5 (0.87)  MA0154.1 EBF1 (0.85)  MA0109.1 Hltf (0.92) | MA0099.2  AP1 (0.90→ 1.00)  MA0102.2  CEBPA (0.86→ 0.81) |
| rs2297339 | MA0103.1 ZEB1 (0.81) | MA0036.1 GATA2 (0.83)  MA0130.1  ZNF354C (0.82) |  |
| rs3807306 |  | MA0442.1 SOX10 (0.81) |  |
| rs4809324 | MA0042.1 FOXI1 (0.84)  MA0041.1 Foxd3 (0.86)  MA0148.1 FOXA1 (0.80)  MA0084.1 SRY (0.83) | MA0038.1 Gfi (0.81)  MA0113.1 NR3C1 (0.81) |  |
| rs4821544 | MA0037.1 GATA3 (0.83)  MA0036.1 GATA2 (0.87) | MA0164.1 Nr2e3 (0.80) | MA0055.1  Myf (0.81→ 0.98)  MA0055.1  Myf (0.97→ 0.82) |
| rs55853698 |  | MA0055.1 Myf (0.82) | MA0048.1  NHLH1 (0.88→ 0.82) |
| rs6958571 | MA0057.1 MZF1_5-13 (0.81)  MA0155.1 INSM1 (0.82) | MA0073.1 RREB1 (0.81)  MA0042.1 FOXI1 (0.80) | MA0079.2  SP1 (0.82→ 0.92) |
| rs737865 | MA0087.1 Sox5 (0.81) | MA0108.2 TBP (0.80)  MA0155.1 INSM1 (0.82)  MA0103.1 ZEB1 (0.81) | MA0003.1  TFAP2A (0.88→ 0.80) |
| rs74393987 |  | MA0163.1 PLAG1 (0.81 | MA0056.1  MZF1_1-4 (0.84→ 0.96)  MA0079.2  SP1 (0.90→ 0.82) |
| rs75612255 |  | MA0130.1  ZNF354C (0.81)  MA0130.1  ZNF354C (0.90) | MA0158.1  HOXA5 (0.87→ 0.81)  MA0079.2  SP1 (0.86→ 0.80) |
| rs75996864 | MA0032.1 FOXC1 (0.86) | MA0092.1  Hand1::Tcfe2a (0.85  MA0145.1 Tcfcp2l1 (0.82)  MA0145.1 Tcfcp2l1 (0.81)  MA0098.1 ETS1 (0.83)  MA0036.1 GATA2 (0.89)  MA0037.1 GATA3 (0.86) | MA0104.2  Mycn (0.80→ 0.92)  MA0259.1  HIF1A::ARNT (0.86→ 0.95) |
| rs76241113 | MA0259.1 HIF1A::ARNT (0.80) | MA0065.2  PPARG::RXRA (0.84)  MA0080.2 SPI1 (0.82) | MA0079.2 SP1 (0.84→ 0.93  MA0056.1  MZF1_1-4 (0.96→ 0.84)  MA0103.1  ZEB1 (0.99→ 0.89) |
| rs77733015 | MA0002.2 RUNX1 (0.83) | MA0088.1 znf143 (0.83) | MA0100.1  Myb (0.99→ 0.92) |
| rs78037487 |  |  |  |
| rs78597499 | MA0002.2 RUNX1 (0.83)  MA0100.1 Myb (0.92)  MA0133.1 BRCA1 (0.93) | MA0073.1 RREB1 (0.80)  MA0162.1 Egr1 (0.81)  MA0006.1  Arnt::Ahr (0.81)  MA0006.1  Arnt::Ahr (0.80)  MA0162.1 Egr1 (0.88)  MA0103.1 ZEB1 (0.84) | MA0079.2  SP1 (0.95→ 0.88) |
| rs79216719 | MA0039.2 Klf4 (0.85)  MA0079.2 SP1 (0.90)  MA0079.2 SP1 (0.84) | MA0131.1 MIZF (0.80)  MA0105.1 NFKB1 (0.80)  MA0061.1  NF-kappaB (0.83)  MA0101.1 REL (0.80) |  |
| rs79488395 | MA0067.1 Pax2 (0.89)  MA0099.2 AP1 (0.81)  MA0089.1  NFE2L1::MafG (0.80)  MA0133.1 BRCA1 (0.82)  MA0063.1 Nkx2-5 (0.80)  MA0092.1  Hand1::Tcfe2a (0.81) | MA0157.1 FOXO3 (0.90) | MA0100.1  Myb (0.87→ 0.99) |
| rs79577178 |  |  | MA0079.2  SP1 (0.88→ 0.82)  MA0079.2  SP1 (0.96→ 0.85)  MA0039.2  Klf4 (0.82→ 0.92)  MA0079.2  SP1 (0.96→ 0.87) |
| rs7961894 | MA0116.1 Zfp423 (0.81)  MA0164.1 Nr2e3 (0.84)  MA0164.1 Nr2e3 (0.80) | MA0141.1 Esrrb (0.81) | MA0259.1  HIF1A::ARNT (0.83→ 0.95)  MA0004.1  Arnt (0.83→ 0.93) |
| rs79734816 | MA0131.1 MIZF (0.80)  MA0061.1  NF-kappaB (0.83)  MA0101.1 REL (0.80)  MA0130.1  ZNF354C (0.81) | MA0039.2 Klf4 (0.81)  MA0079.2 SP1 (0.85) | MA0056.1  MZF1_1-4 (0.84→ 0.96) |
| rs80112297 | MA0162.1 Egr1 (0.86)  MA0073.1 RREB1 (0.80) | MA0065.2  PPARG::RXRA (0.84) | MA0079.2  SP1 (0.90→ 0.96)  MA0056.1  MZF1_1-4 (0.96→ 0.84)  MA0039.2  Klf4 (0.88→ 0.82)  MA0079.2  SP1 (0.89→ 0.96)  MA0057.1  MZF1_5-13 (0.96→ 0.83) |
| rs80313086 | MA0162.1 Egr1 (0.86)  MA0073.1 RREB1 (0.80) | MA0065.2  PPARG::RXRA (0.84) | MA0079.2  SP1 (0.90→ 0.96)  MA0056.1  MZF1_1-4 (0.96→ 0.84)  MA0039.2  Klf4 (0.88→ 0.82)  MA0079.2  SP1 (0.89→ 0.96)  MA0057.1  MZF1_5-13 (0.96→ 0.83) |
| rs9465871 | MA0137.2 STAT1 (0.81)  MA0158.1 HOXA5 (0.83)  MA0027.1 En1 (0.82)  MA0158.1 HOXA5 (0.82)  MA0075.1 Prrx2 (0.81)  MA0102.2 CEBPA (0.81)  MA0084.1 SRY (0.81) | MA0035.2 Gata1 (0.81)  MA0080.2 SPI1 (0.80) |  |
| rs113994210 | MA0039.2 Klf4 (0.80)  MA0117.1 Mafb (0.83) |  | MA0146.1 Zfx (0.82→ 0.95)  MA0146.1 Zfx (0.94→ 0.82)  MA0003.1  TFAP2A (0.88→ 0.80) |
| rs3057 |  | MA0070.1 PBX1(0.80)  MA0077.1 SOX9 (0.82)  MA0070.1 PBX1 (0.85) |  |
| rs3766379 | MA0099.2 AP1 (0.81)  MA0032.1 FOXC1 (0.96) | MA0161.1 NFIC (0.82)  MA0130.1 ZNF354C (0.87)  MA0037.1 GATA3 (0.82)  MA0003.1 TFAP2A (0.87) |  |
