# Supplementary material for: Detection of Regulatory SNPs in Human Genome Using ChIP-seq ENCODE Data
Source: PLoS One. 2013 Oct 29;8(10):e78833. doi: 10.1371/journal.pone.0078833 (PMC3812152; doi:10.1371/journal.pone.0078833)
Supplement: Document S2 — The algorithm for data management and analysis used in the study. (DOC) [file pone.0078833.s008.doc]

**Document S2.** The algorithm for data management and analysis used in the study.

1. MySQL commands used for creation and querying publically available data tables used in the analysis

#create MySQL table with coordinates and the number of overlapping TF binding loci for OTFRs.

create table TxN (chromosome varchar(10) , start int(20), end int(20), number_of_overlapping peaks int)

load data infile “TxN.txt” into table TxN

#create MySQL table (signed here as <analyzed table>) with SNP coordinates for each sample of SNPs. Input tables, containing only “chrom” and “chromStart” fields, can be generated with UCSC Table Browser website (genome.ucsc.edu/cgi-bin/hgTables) using “filter” and “output format” options.

create table <analyzed table> (chromosome varchar(10) , start int(20))

load data infile “<analyzed table>” into table <analyzed table>

# create MySQL table for the sample of random SNPs. The coordinates for the set of all SNPs can be similarly downloaded from UCSC Table Browser website using the filter “by-1000genomes”

create table <1000genomes_SNP_table> (chromosome varchar(10) , start int(20))

load data infile “<1000genomes_SNP_table>” into table <1000genomes_SNP_table>

create table RND_SNP select chromosome,position,name from <1000genomes_SNP_table> order by rand() limit 1000000

#numbering strings in tables

alter table <analyzed table> add id_SNP int not null auto_increment, add primary key(id_SNP)

alter table TxN add id int not null auto_increment, add primary key(id_SNP)

#select all SNPs from <analyzed table>, falling into OTFRs

create table <overlap SNP with OTFR> select id, id_SNP from <analyzed table> as A, TxN as B where A.chromosome =B. chromosome and B.start<A. position and B.end>A. position

2. Perl script used to analyze MySQL tables and generate bootstrap samples (requires DBI, and LWP libraries)

#!/usr/bin/perl

#use strict;

use DBI;

use LWP::UserAgent;

my $bd="SNP";

$dbh = DBI->connect("DBI:mysql:$bd","root","197714")||error(($dbh->state),"ddd");

my $sth = $dbh -> prepare(qq{SET NAMES cp1251});

my $rv = $sth -> execute or die " : $sth -> errstr";

$tab='<analyzed table>';

$tab1='<overlap SNP with OTFR> ';

$Otab="TxN";

$tag='tags';

open(FD,"><result file>");

$sd1="select count(1) from $tab ";

my $sth = $dbh -> prepare(qq{$sd1});

my $rv = $sth -> execute||error(($dbh->state),$sd1);

$Ntab=$sth->fetchrow_array();

$sd1="select count(1) from $Otab ";

my $sth = $dbh -> prepare(qq{$sd1});

my $rv = $sth -> execute||error(($dbh->state),$sd1);

$NTxN=$sth->fetchrow_array();

for($i=0;$i<500;$i++){

$sd1="drop table 2tmp";

my $sth = $dbh -> prepare(qq{$sd1});

my $rv = $sth -> execute||error(($dbh->state),$sd1);

$sd1="create table 2tmp select 1+round(rand()*$NTxN) as id from $Otab";

my $sth = $dbh -> prepare(qq{$sd1});

my $rv = $sth -> execute||error(($dbh->state),$sd1);

$sd1="alter table 2tmp add index id(id)";

my $sth = $dbh -> prepare(qq{$sd1});

my $rv = $sth -> execute||error(($dbh->state),$sd1);

$sd1="drop table 2tmp1";

my $sth = $dbh -> prepare(qq{$sd1});

my $rv = $sth -> execute||error(($dbh->state),$sd1);

$sd1="create table 2tmp1 select 1+round(rand()*$Ntab) as id_SNP from $tab ";

my $sth = $dbh -> prepare(qq{$sd1});

my $rv = $sth -> execute||error(($dbh->state),$sd1);

$sd1="drop table 2tmp2";

my $sth = $dbh -> prepare(qq{$sd1});

my $rv = $sth -> execute||error(($dbh->state),$sd1);

$sd1="create table 2tmp2 select id, start, end, $tag from 2tmp inner join $Otab using(id)";

my $sth = $dbh -> prepare(qq{$sd1});

my $rv = $sth -> execute||error(($dbh->state),$sd1);

$sd1="drop table 2tmp3";

my $sth = $dbh -> prepare(qq{$sd1});

my $rv = $sth -> execute||error(($dbh->state),$sd1);

$sd1="create table 2tmp3 select * from 2tmp1 inner join $tab1 using(id_SNP) inner join 2tmp using(id) ";

my $sth = $dbh -> prepare(qq{$sd1});

my $rv = $sth -> execute||error(($dbh->state),$sd1);

$RS='';

for($iii=1; $iii<50;$iii=$iii+1){

$sd1="select sum(end-start) from 2tmp2 where $tag>$iii";

my $sth = $dbh -> prepare(qq{$sd1});

my $rv = $sth -> execute||error(($dbh->state),$sd1);

$St=$sth->fetchrow_array();

$sd1="select count(1) from 2tmp3 where $tag>$iii";

my $sth = $dbh -> prepare(qq{$sd1});

my $rv = $sth -> execute||error(($dbh->state),$sd1);

$R=$sth->fetchrow_array();

#print "$St $R \n";

$R= $R/$St/$Ntab*3000000000;

$R=~/(\d+.\d\d)/;

$RS="$RS\x090$1";

}

print "$i $RS \n";

print FD "$RS\n";

}

#The resulting output table contains enrichment values in 50 columns, corresponding to different OTFR *i* values [2;50], and 500 rows, corresponding to each bootstrap iteration.
