# Supplementary material for: Detection of Regulatory SNPs in Human Genome Using ChIP-seq ENCODE Data
Source: PLoS One. 2013 Oct 29;8(10):e78833. doi: 10.1371/journal.pone.0078833 (PMC3812152; doi:10.1371/journal.pone.0078833)
Supplement: Document S3 — Putative rSNPs selected for EMSA and their position within OTFRs with i ≥7. (DOC) [file pone.0078833.s009.doc]

**Document S3.** Putative rSNPs selected for EMSA and their position within OTFRs with *i*≥7.

| SNP identifier in dbSNP NCBI | SNPs position wihtin ChIP-seq peaks overlaps |
| --- | --- |
| rs10411210 | 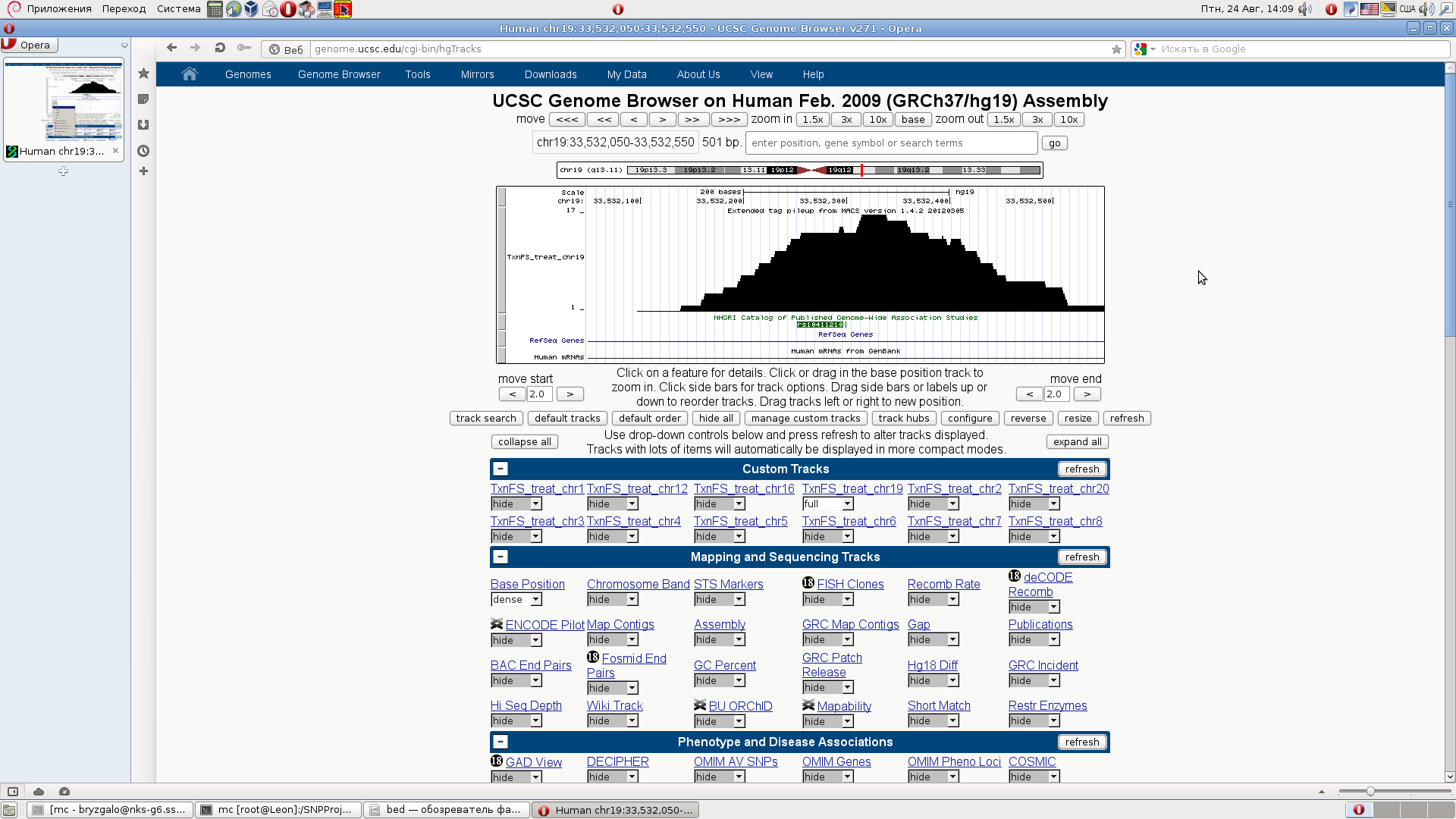 |
| rs1048990 | 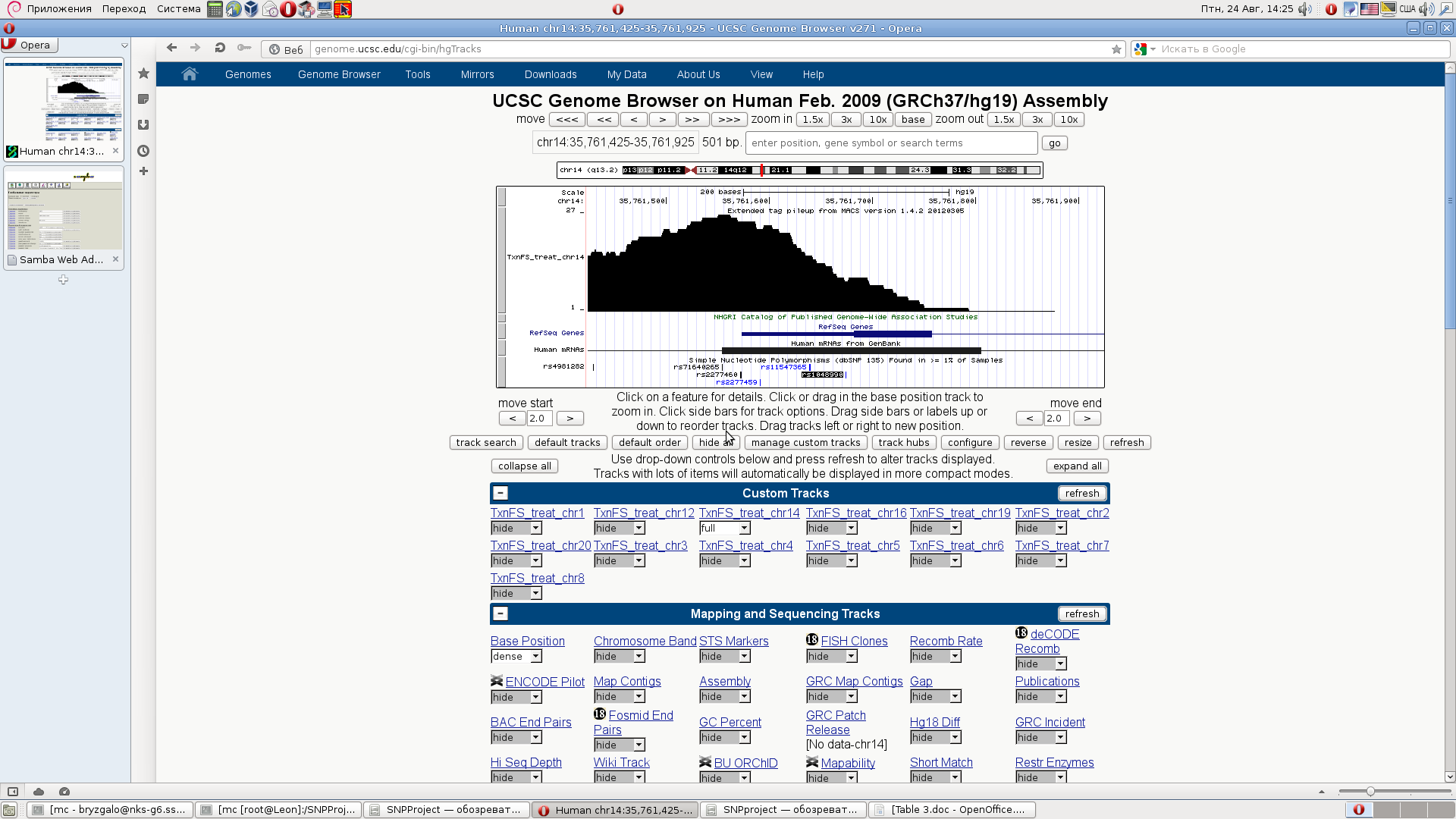 |
| rs11178998 | 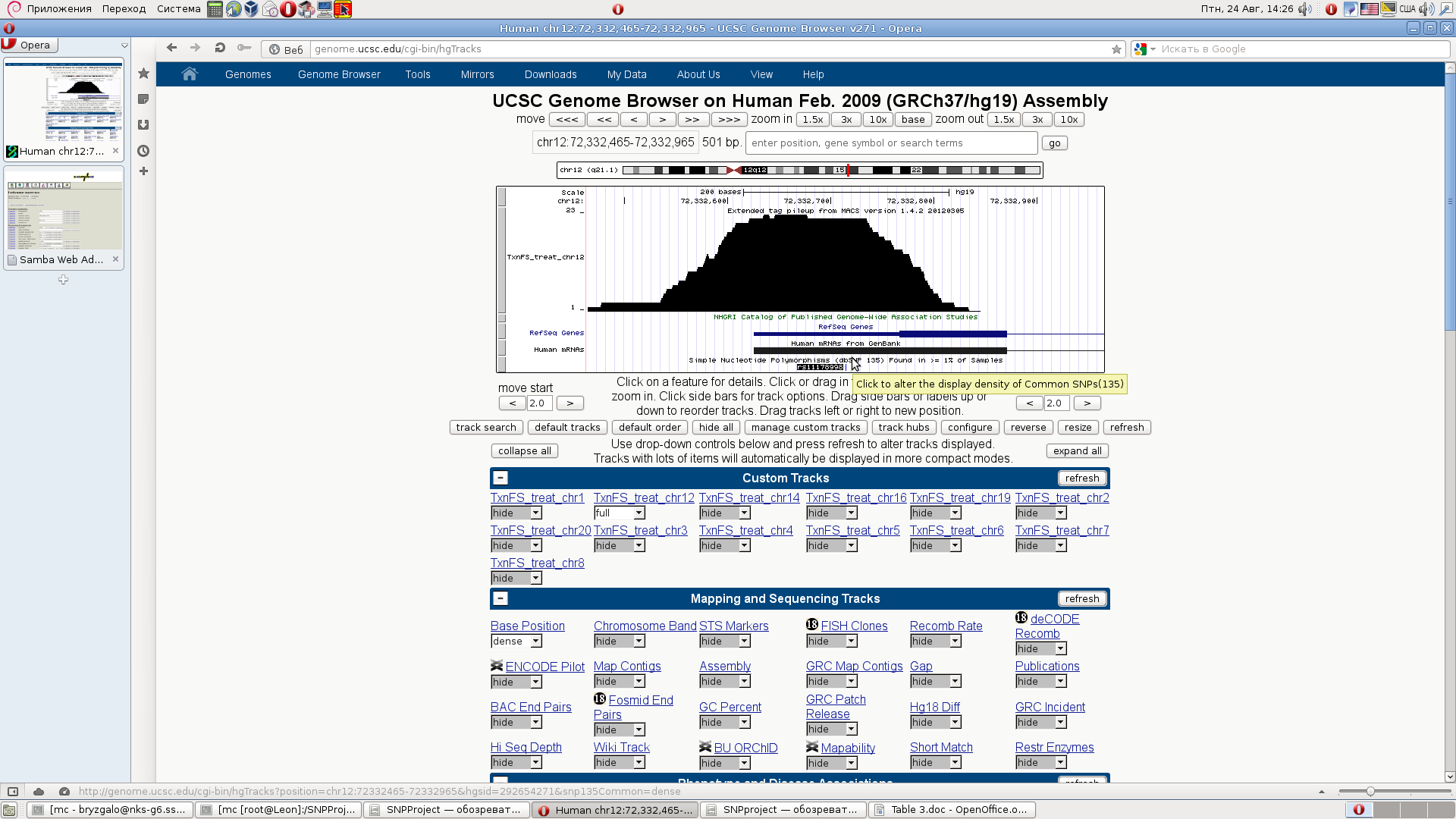 |
| rs113994210 | 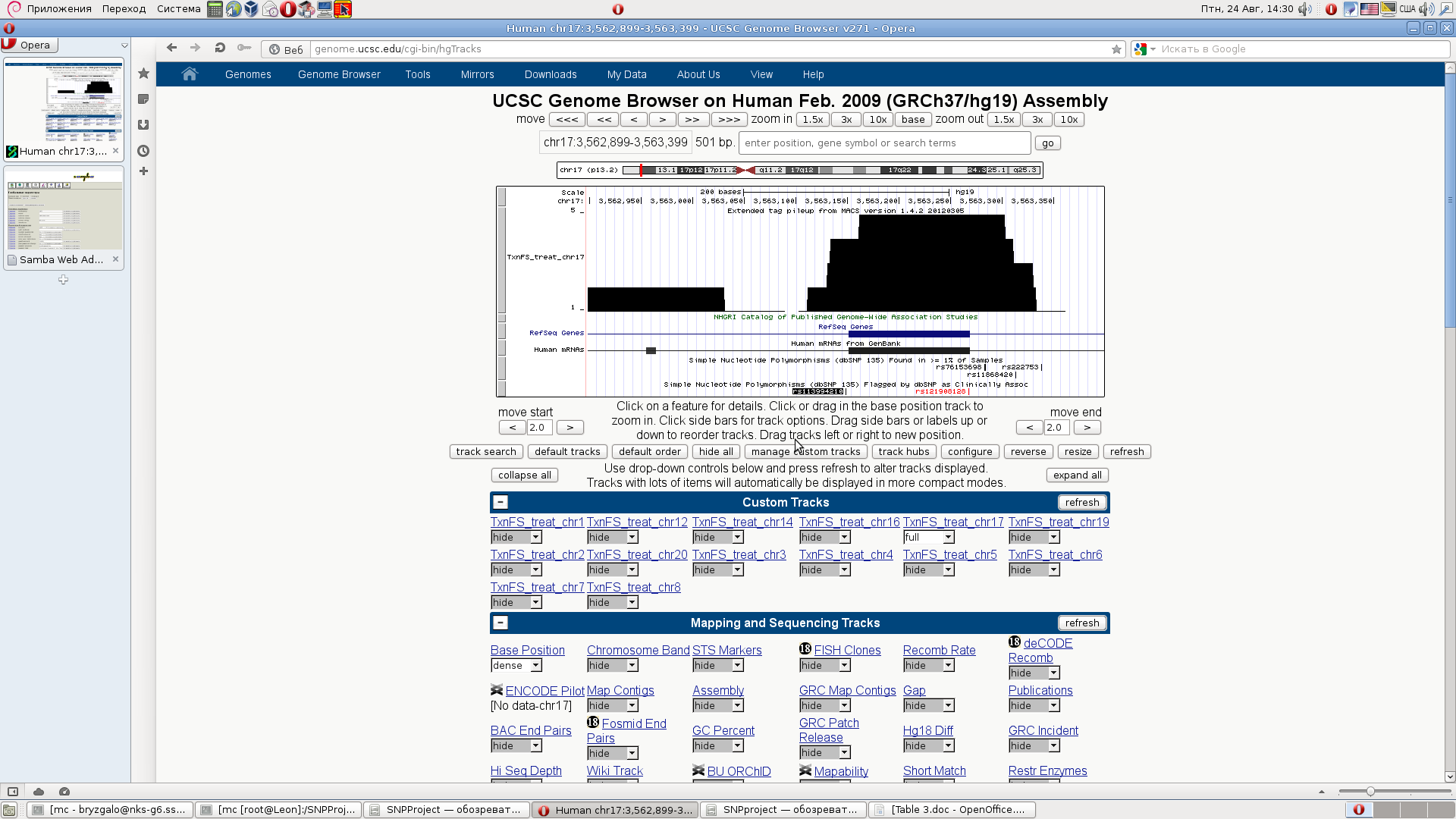 |
| rs11466315 | 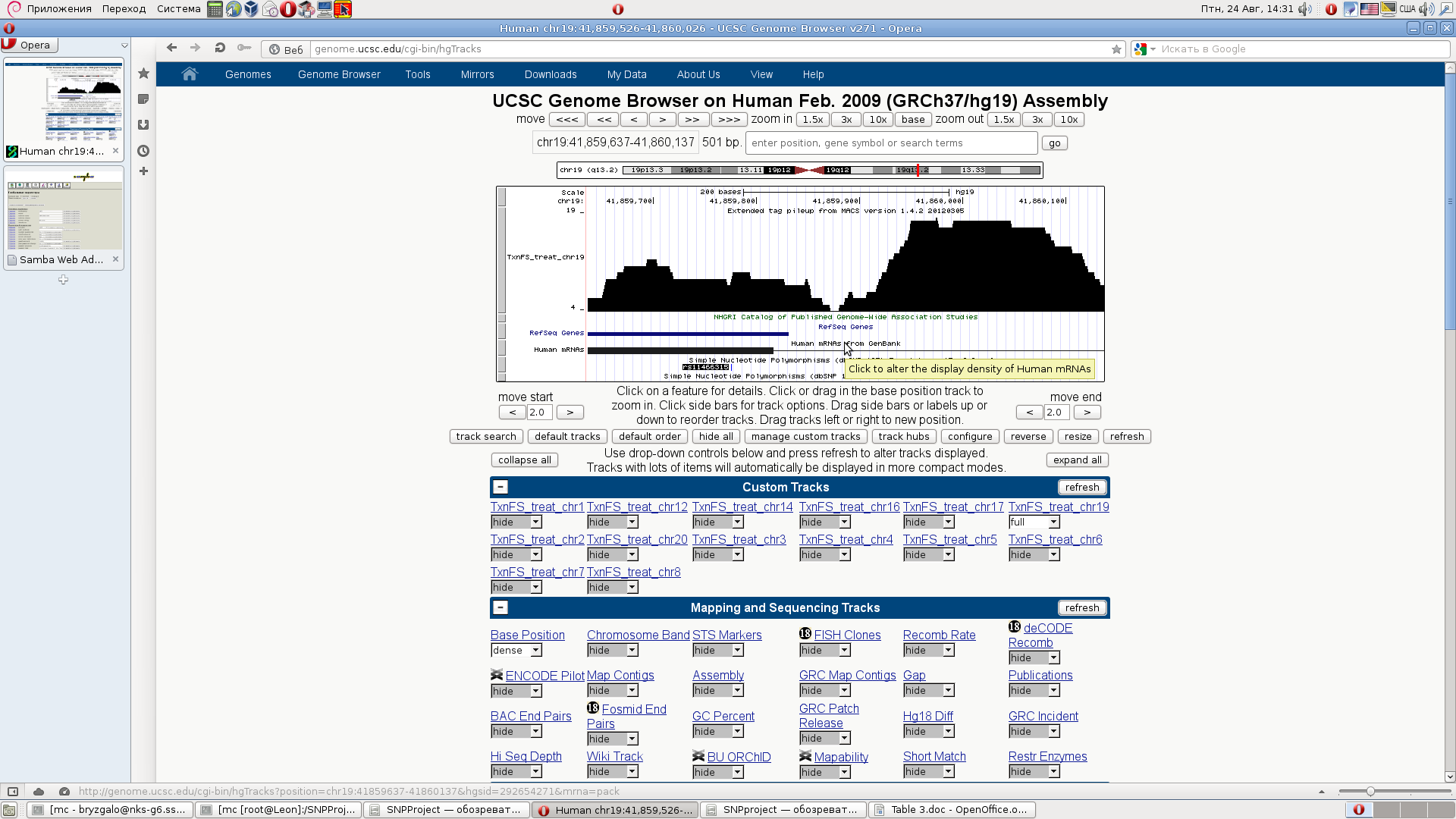 |
| rs12044852 | 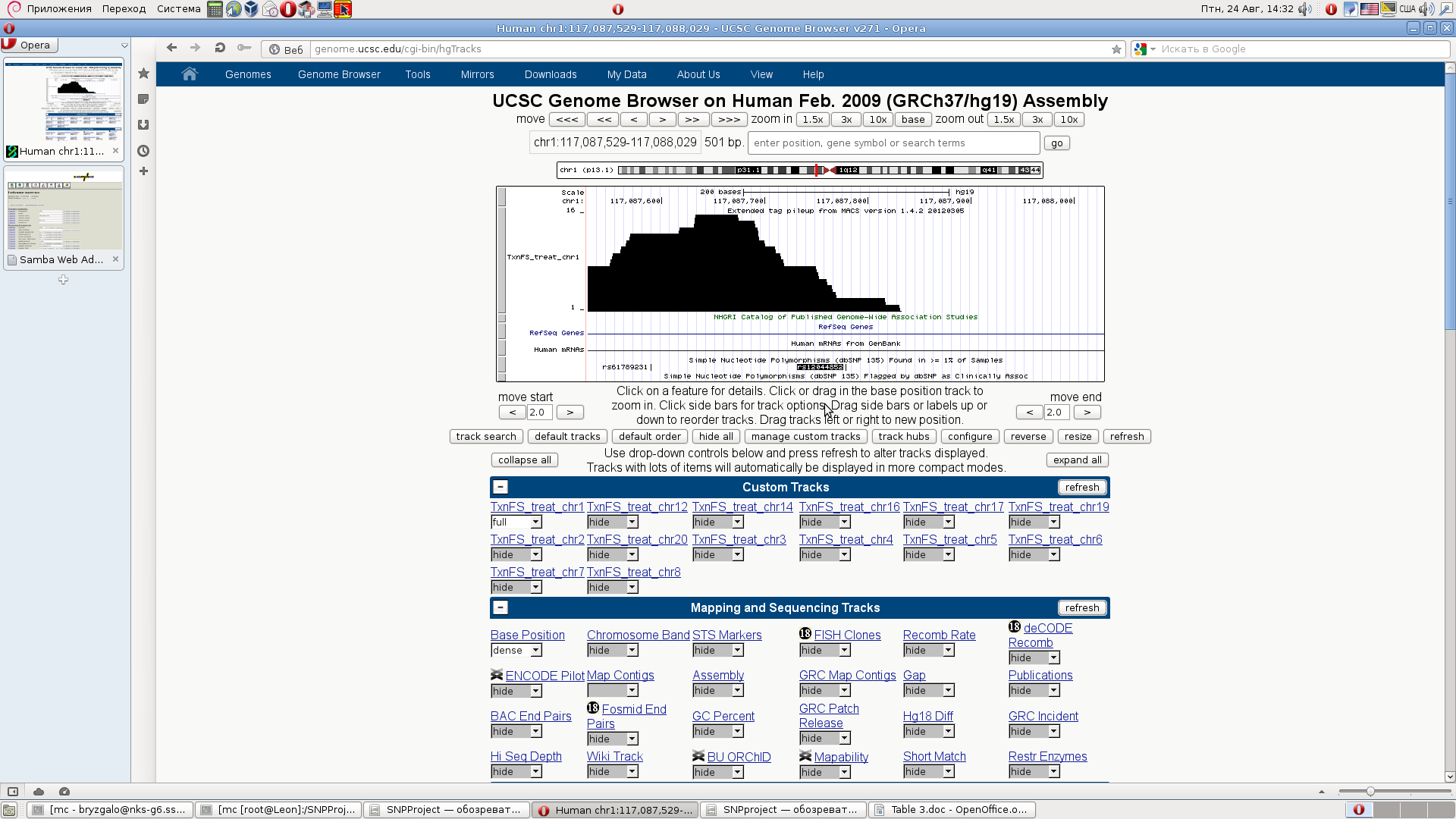 |
| rs12740374 | 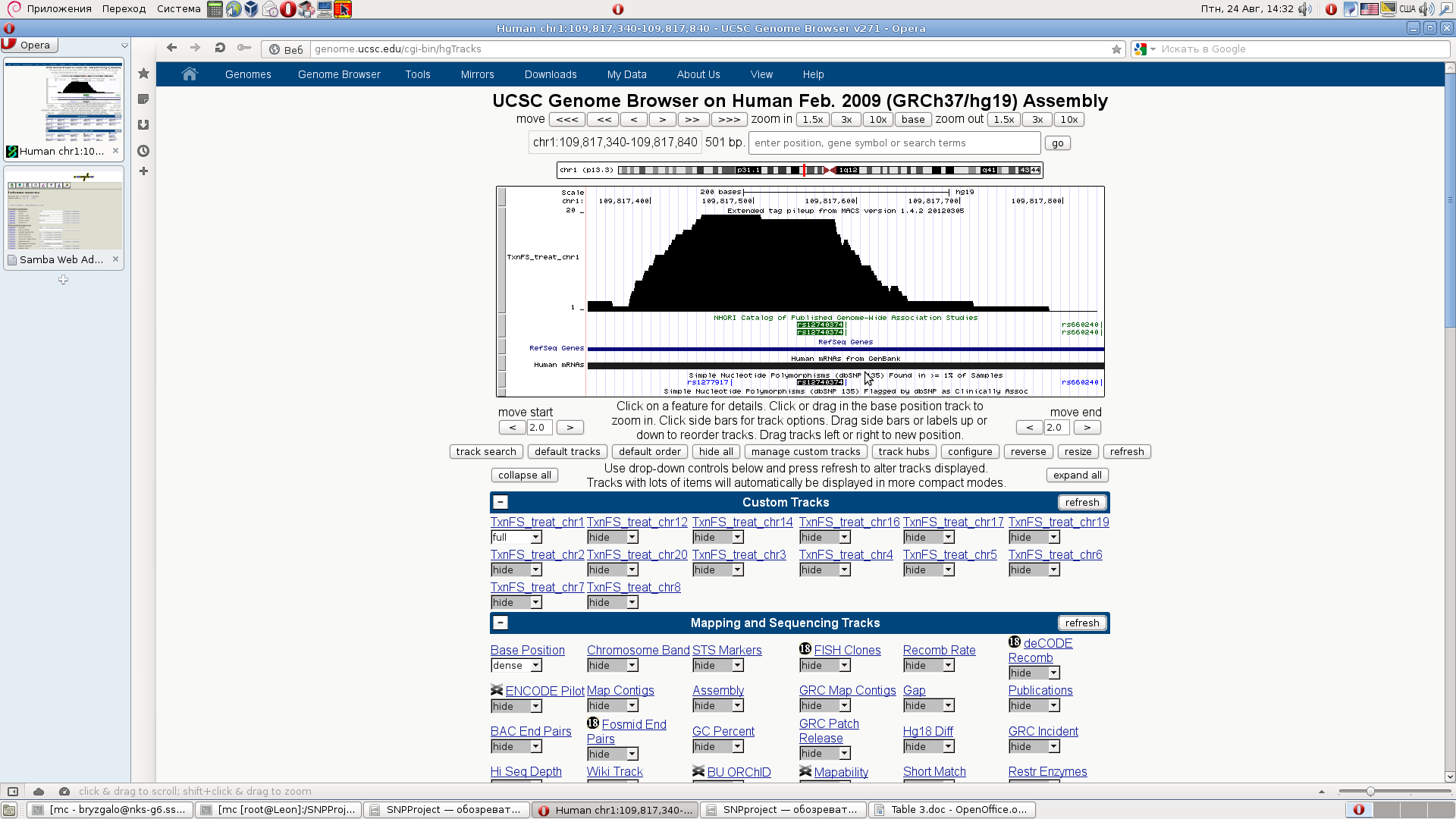 |
| rs12885713 | 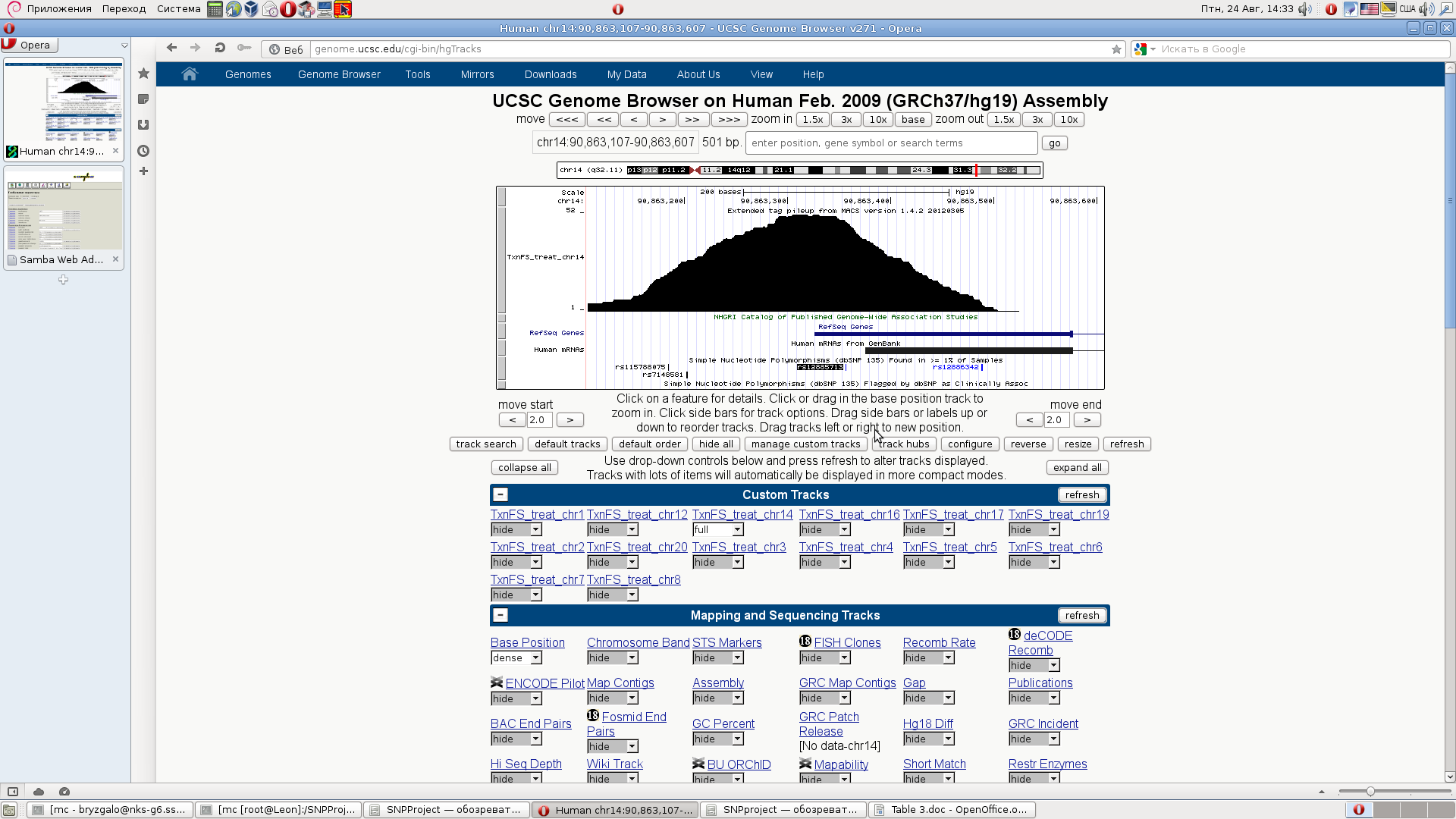 |
| rs1532624 | 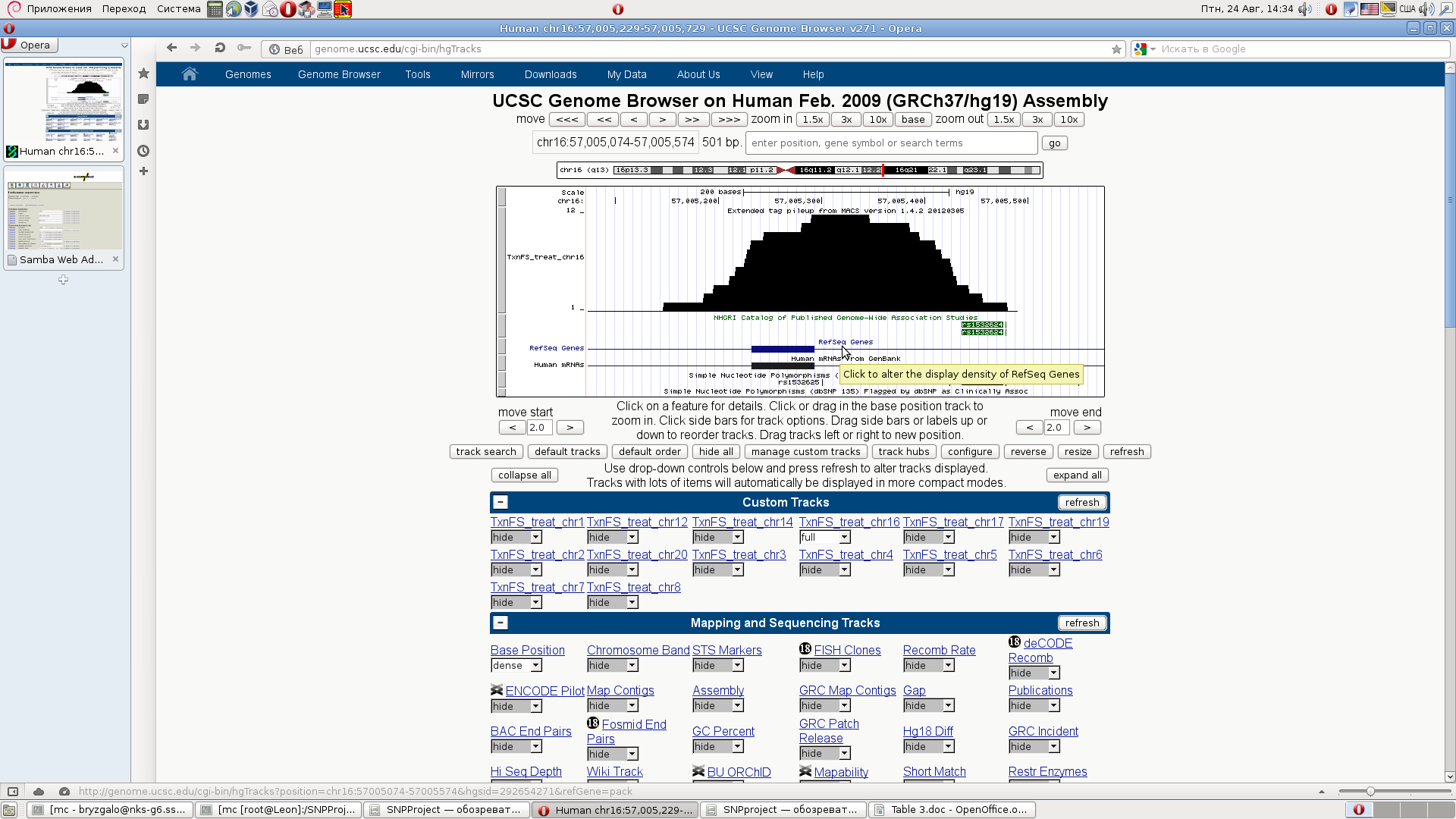 |
| rs17039192 | 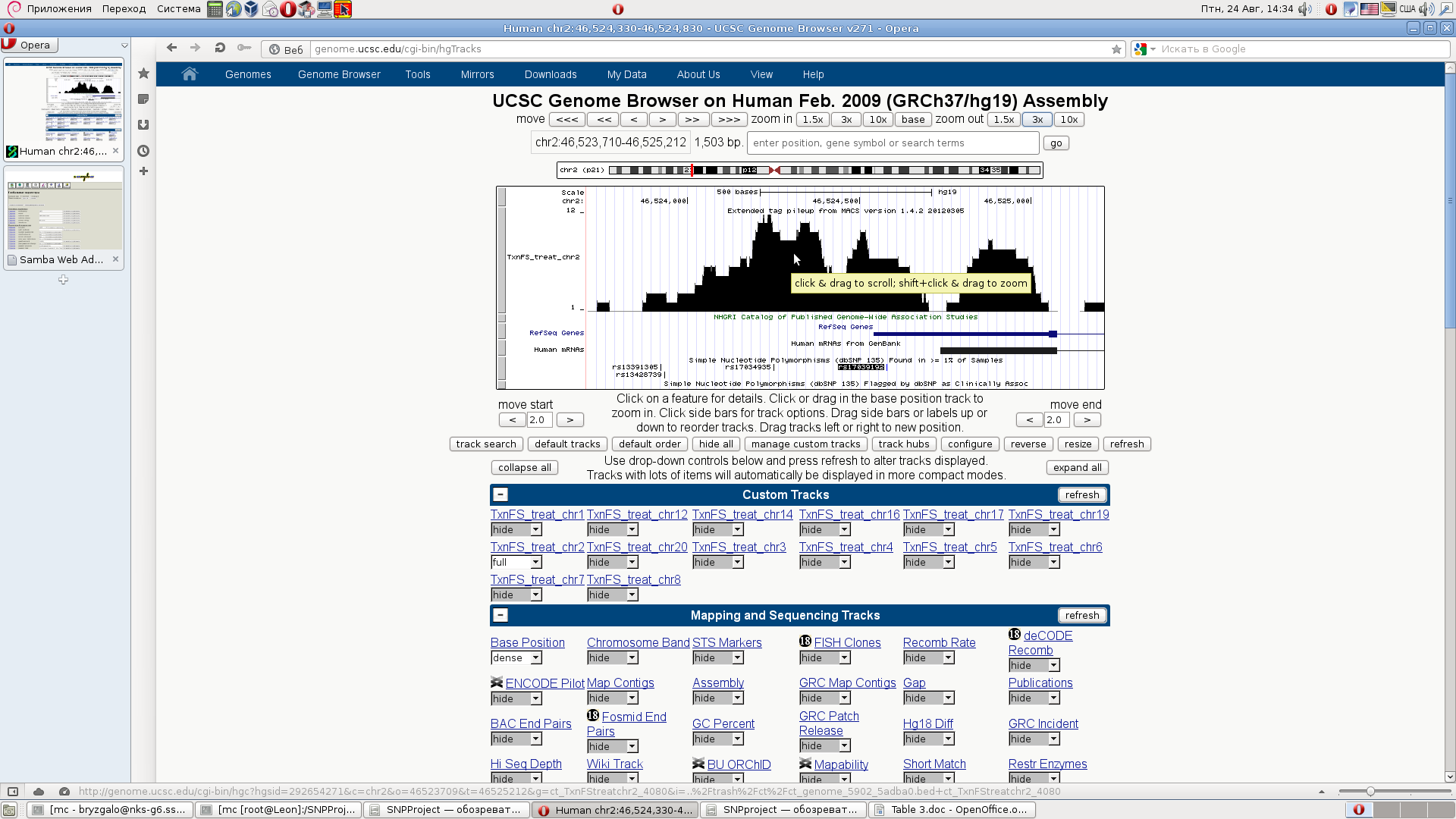 |
| rs1800734 | 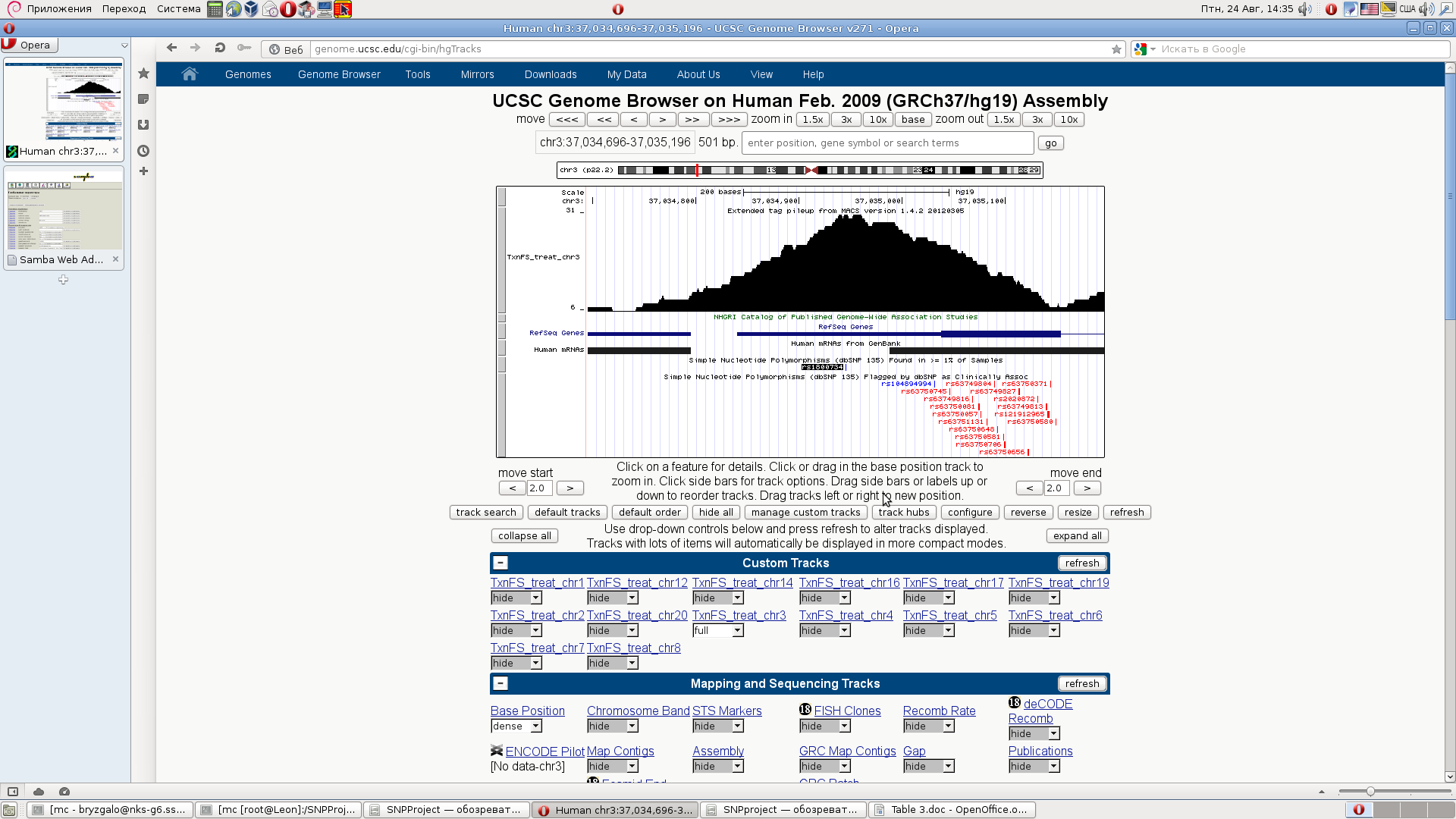 |
| rs2010963 | 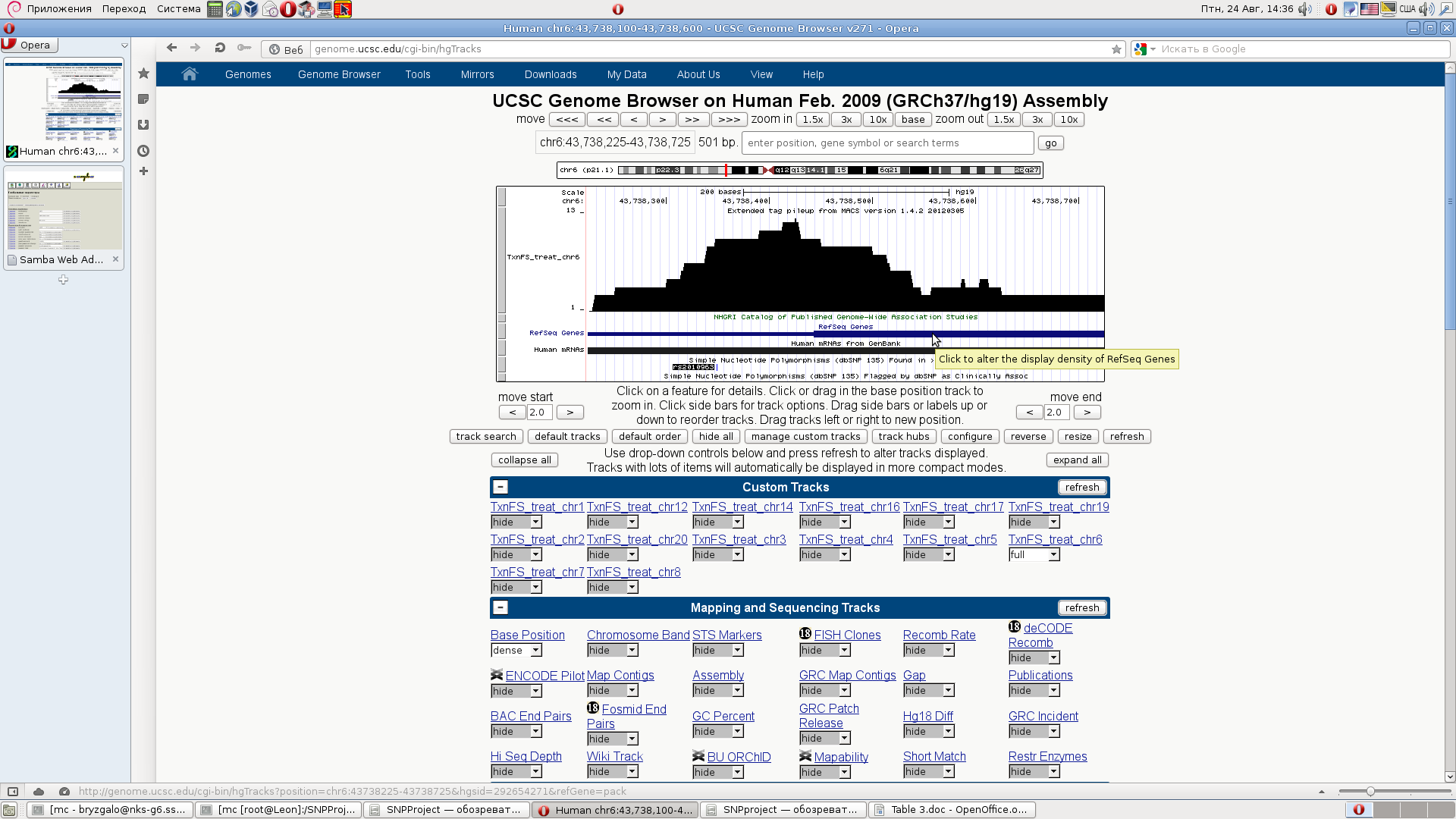 |
| rs2038137 | 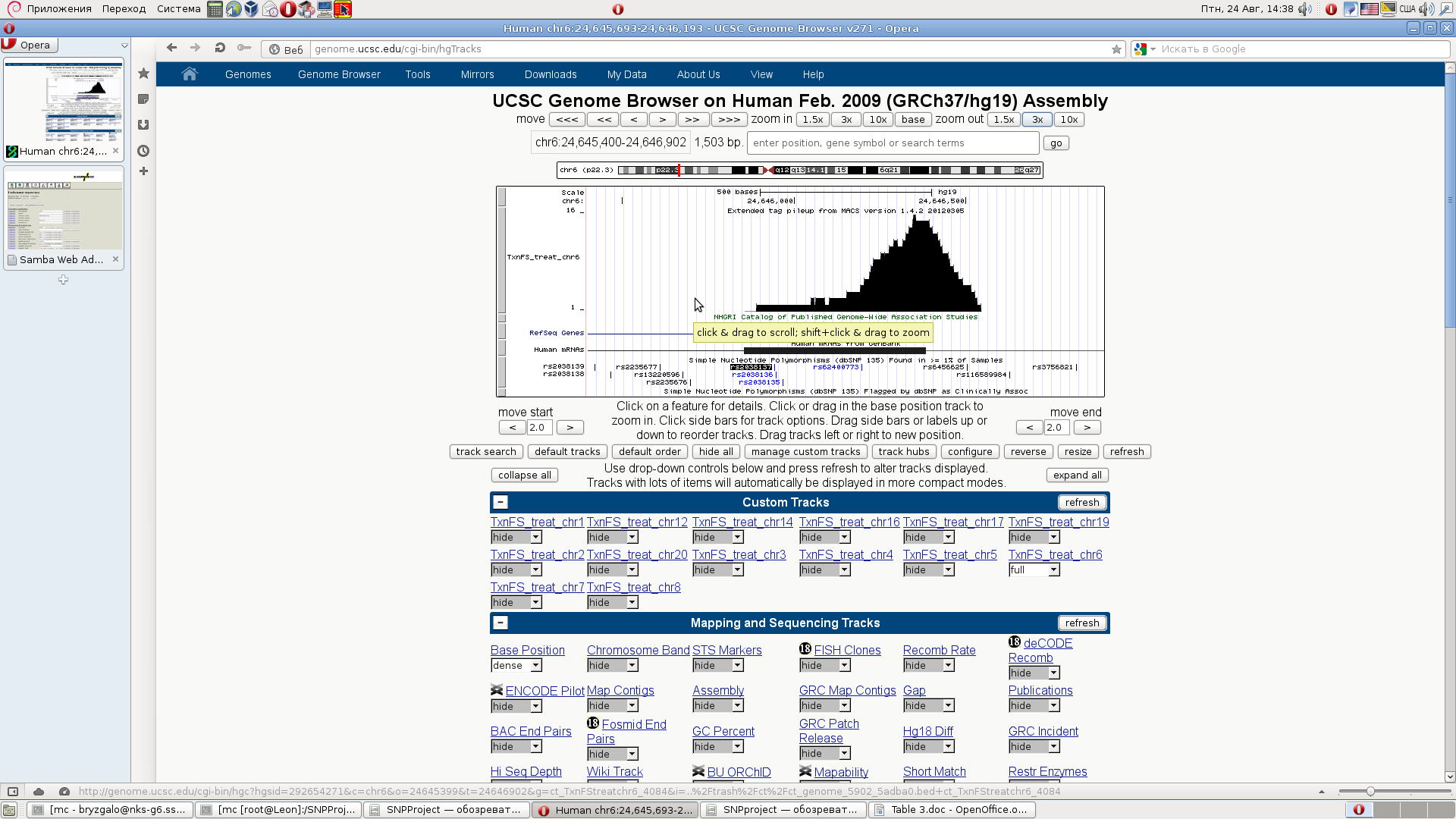 |
| rs2071002 | 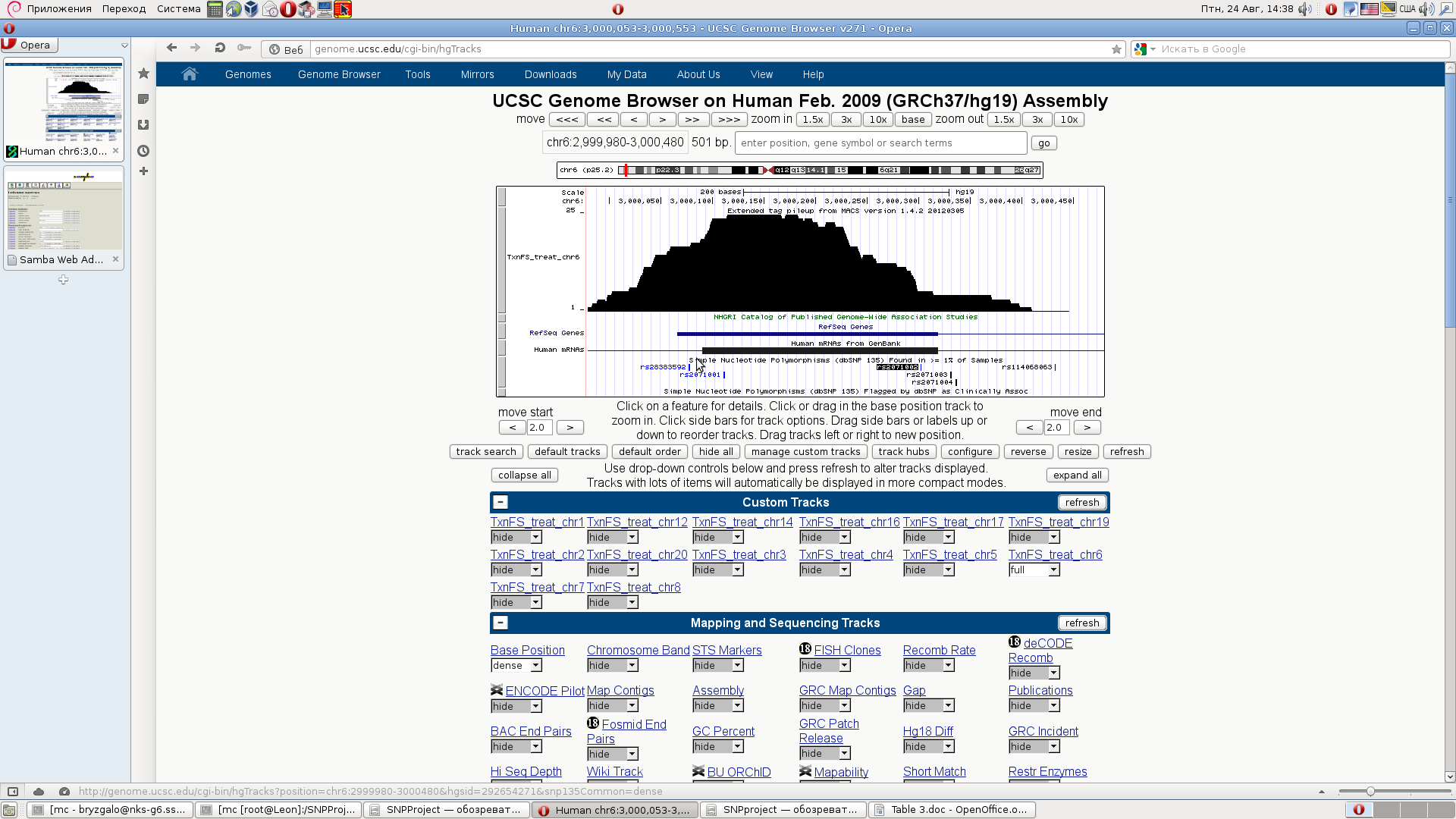 |
| rs2279744 | 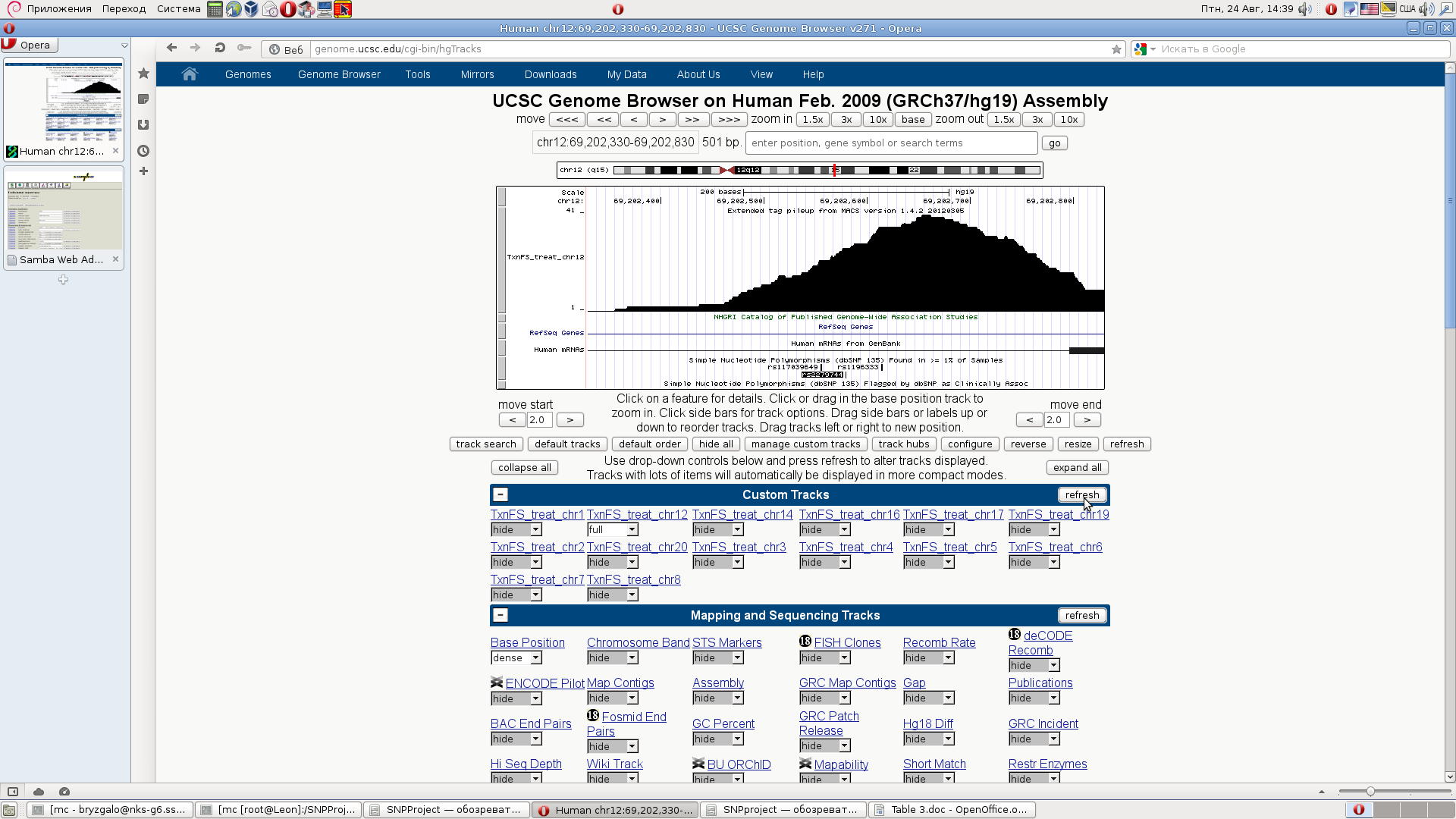 |
| rs2282978 | 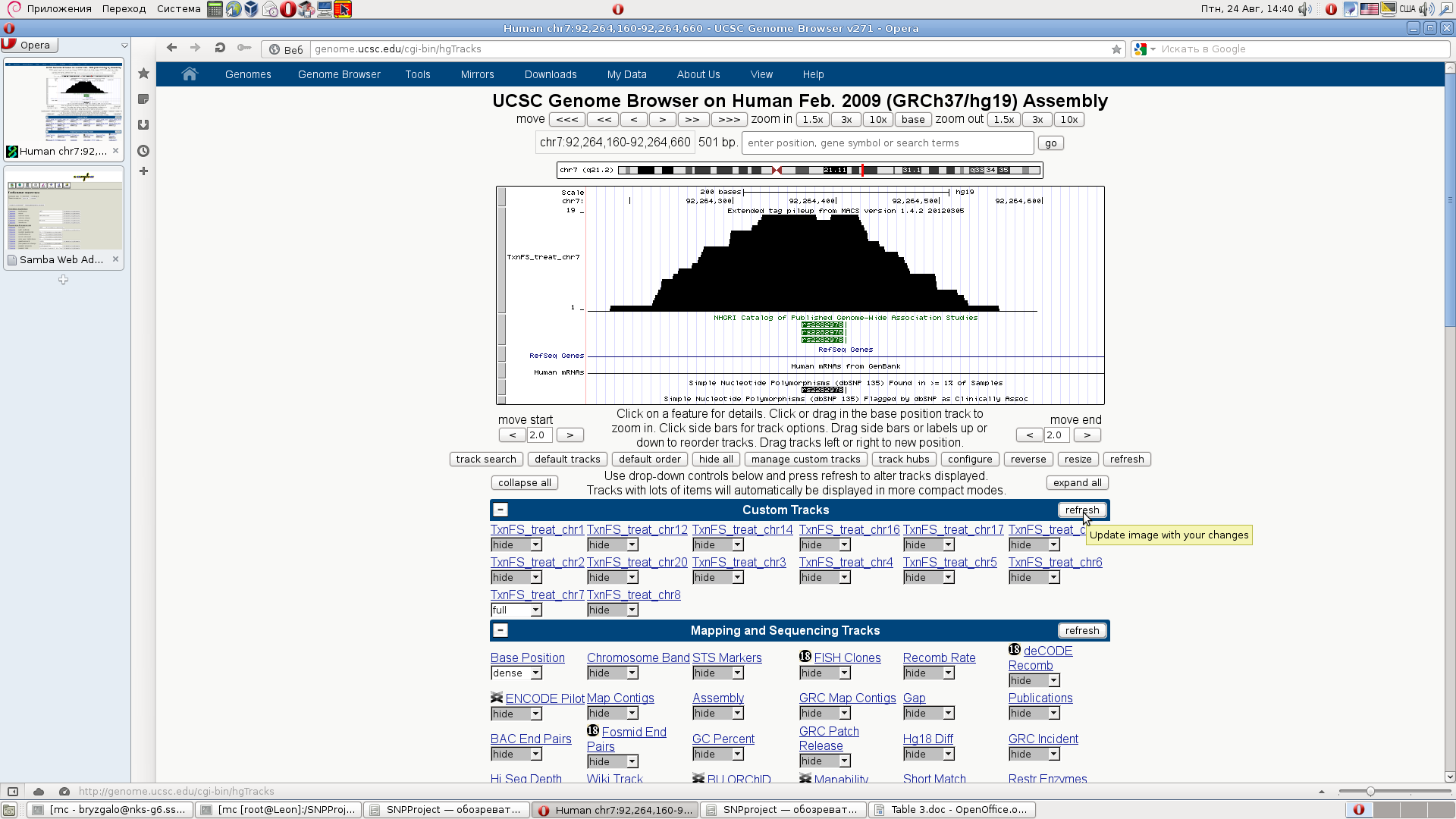 |
| rs2297339 | 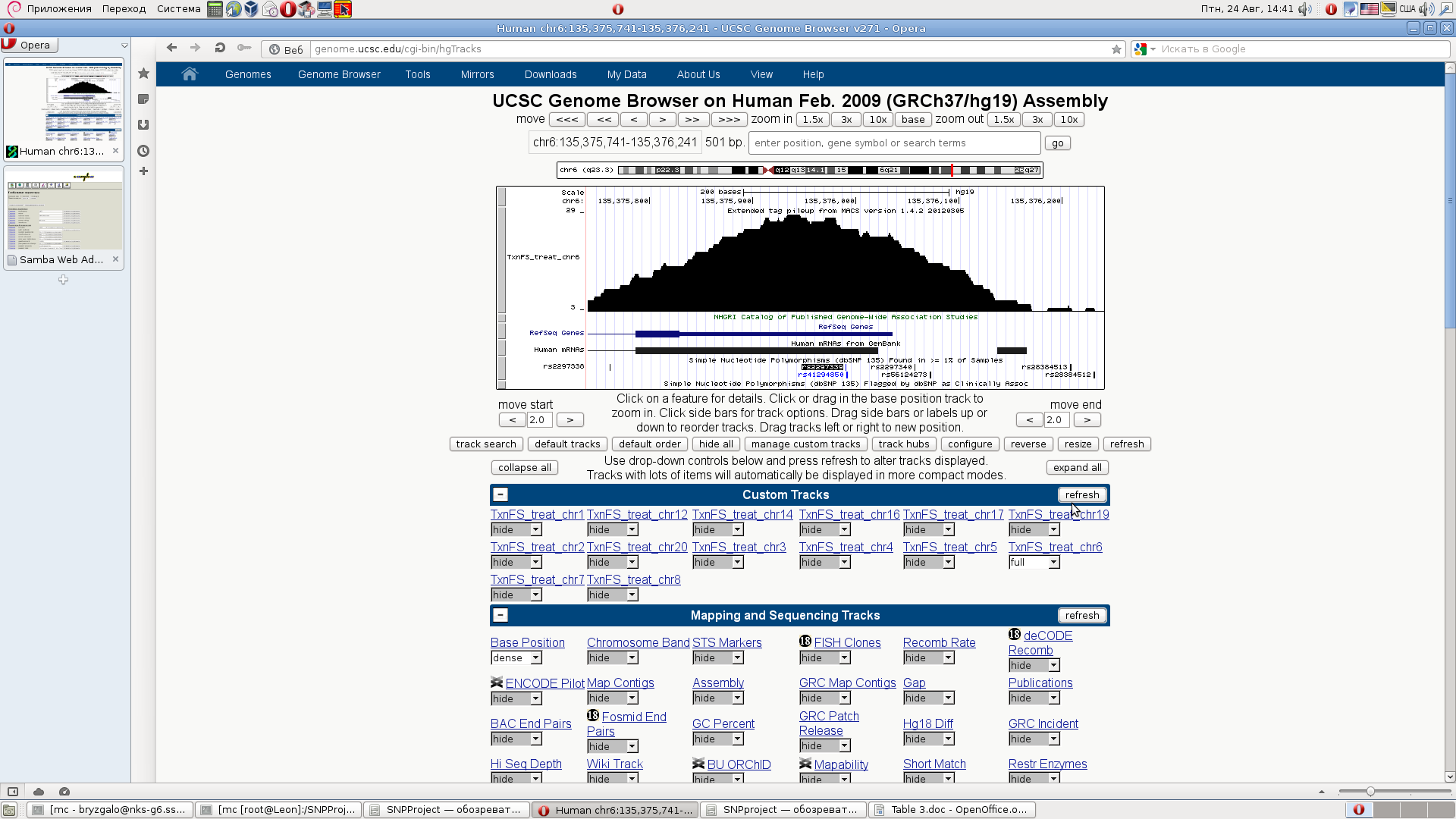 |
| rs3807306 | 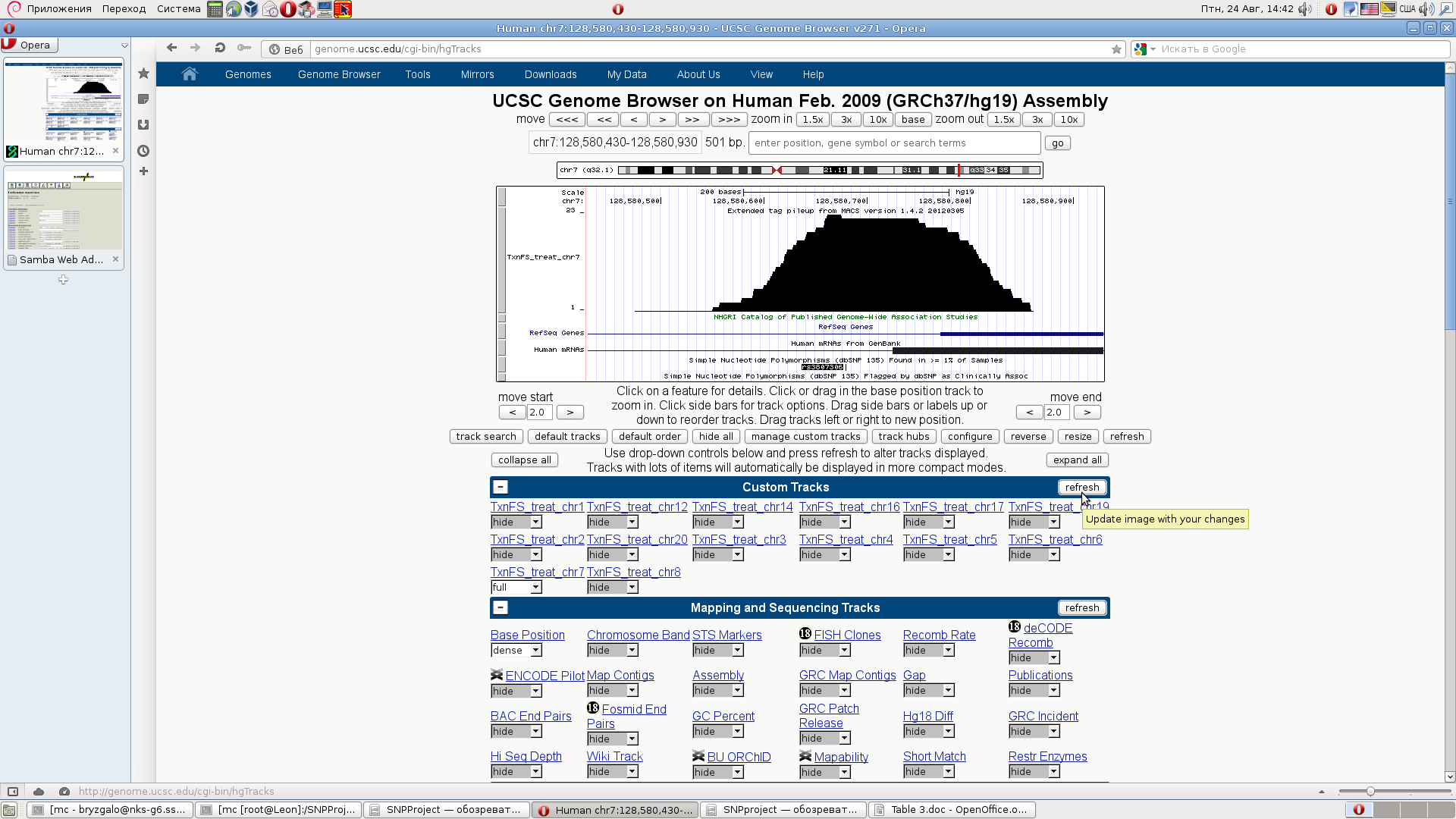 |
| rs4809324 | 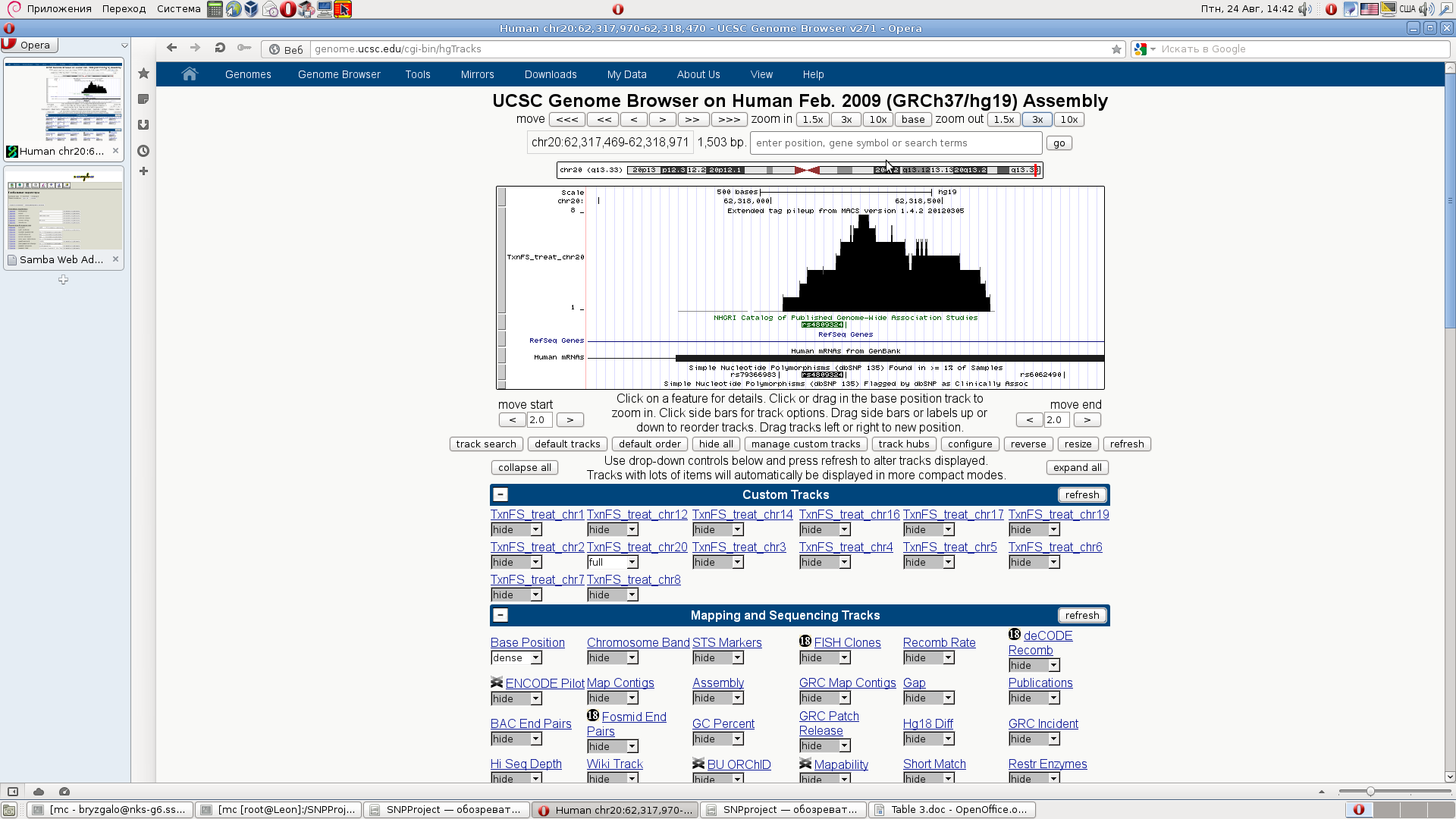 |
| rs55853698 | 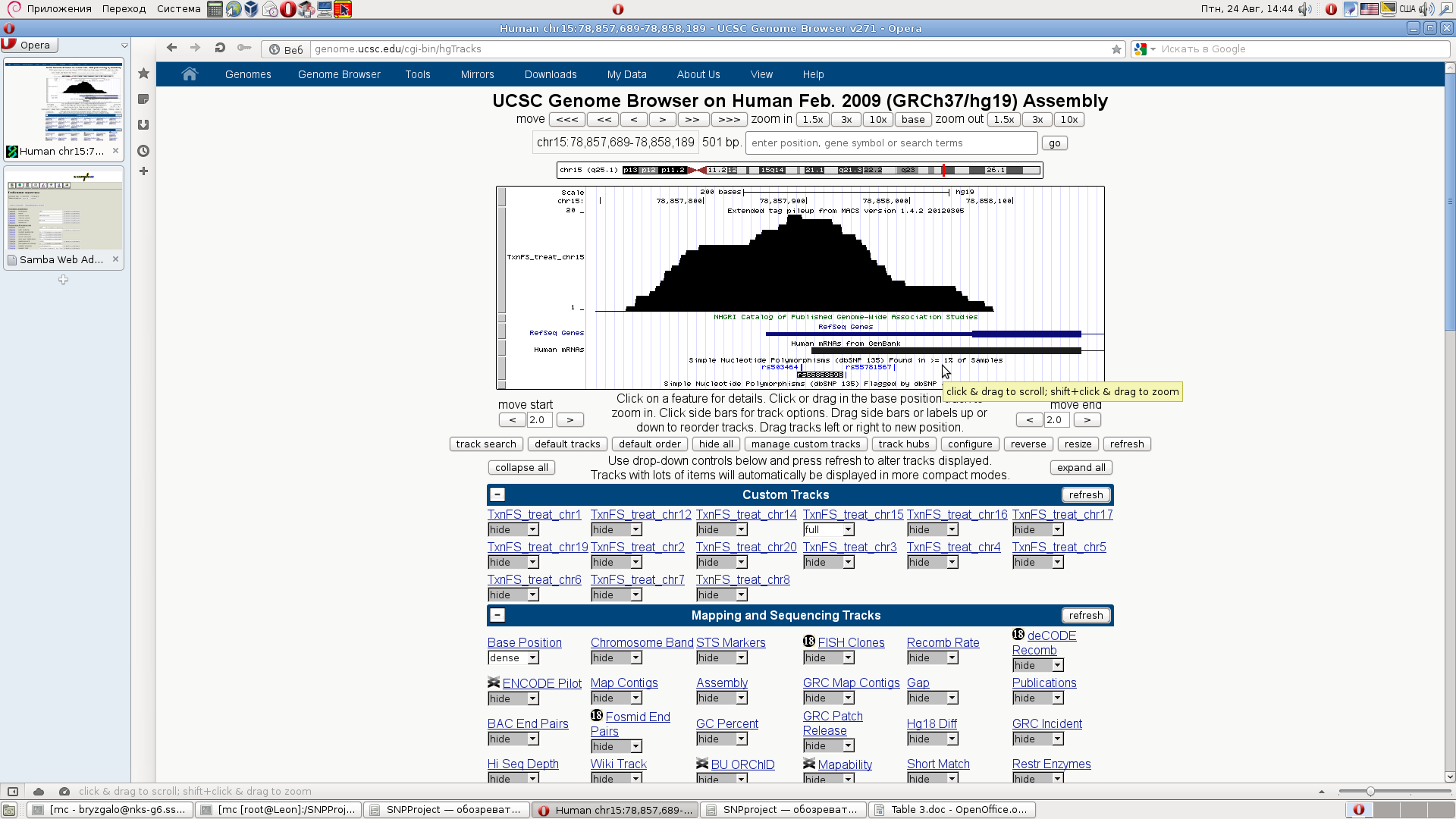 |
| rs6958571 | 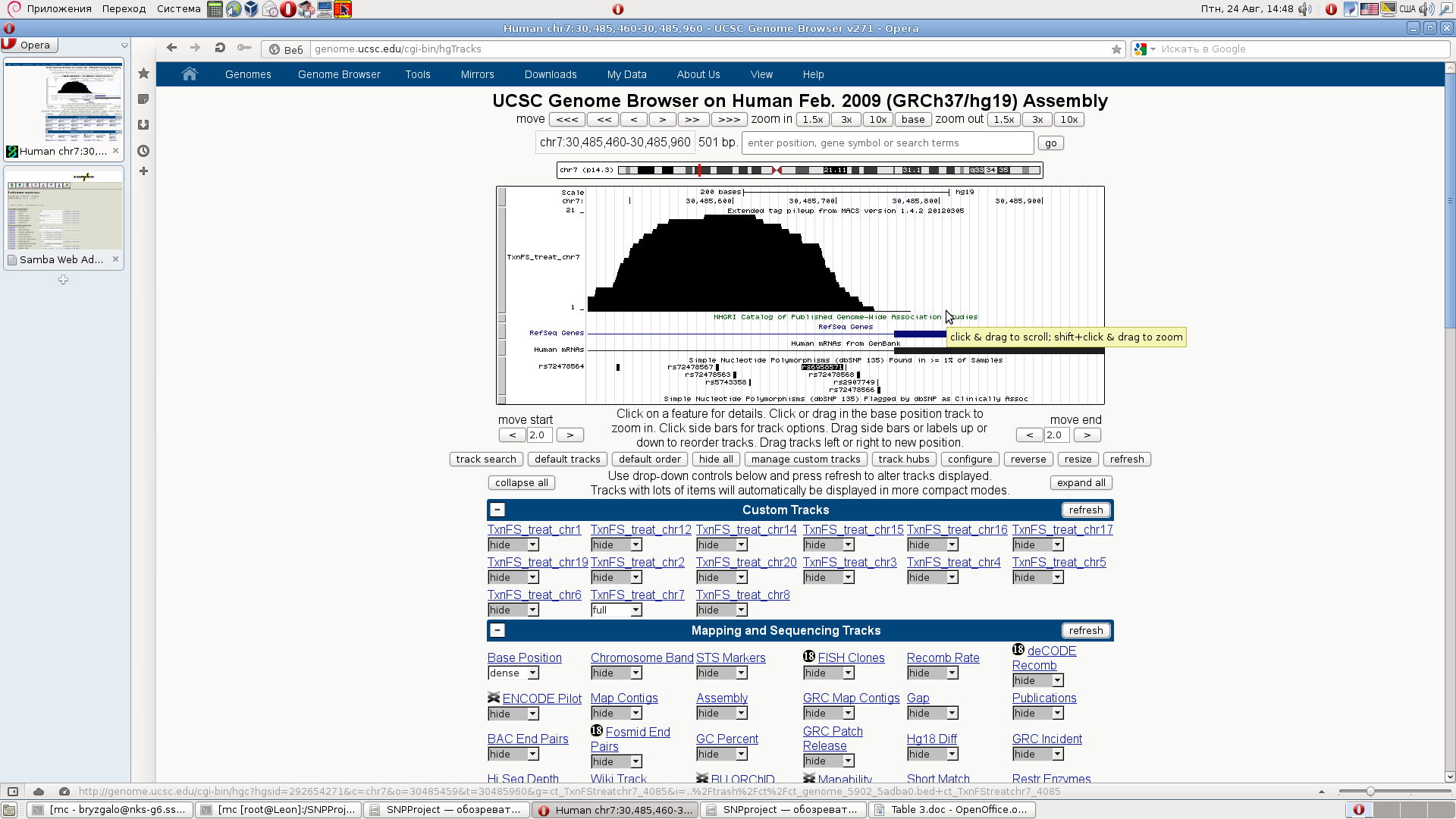 |
| rs737865 | 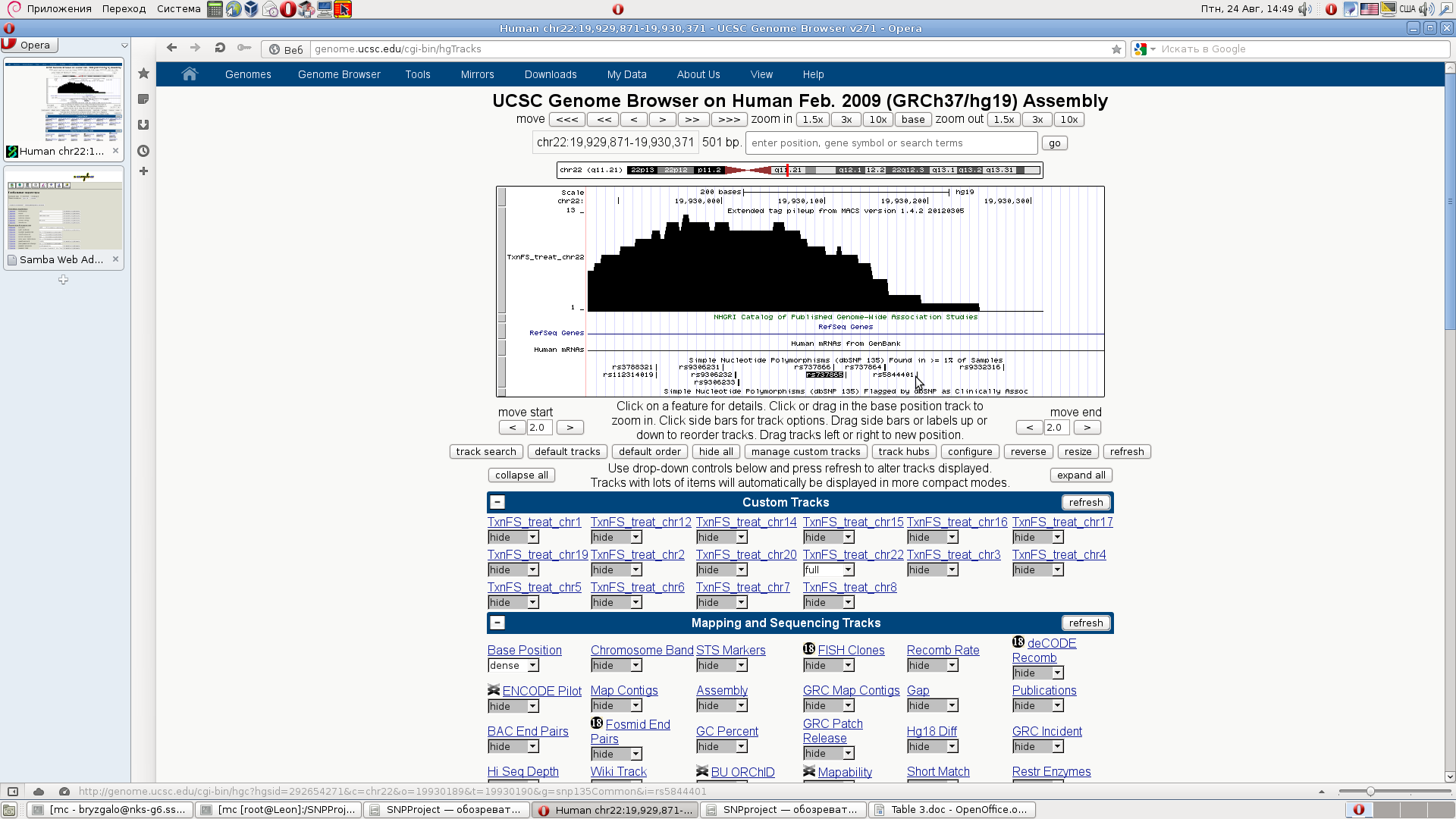 |
| rs74393987 | 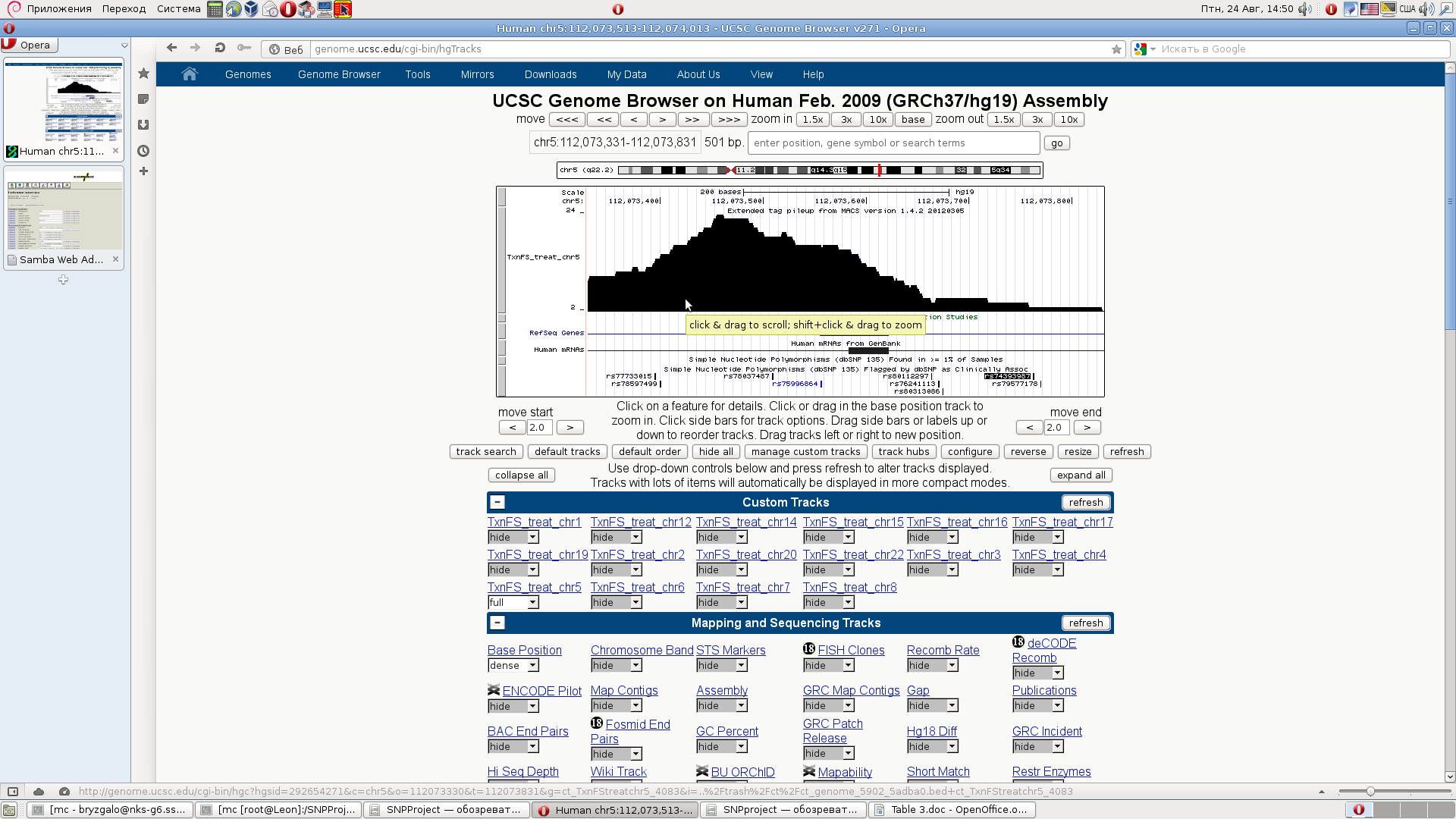 |
| rs75612255 | 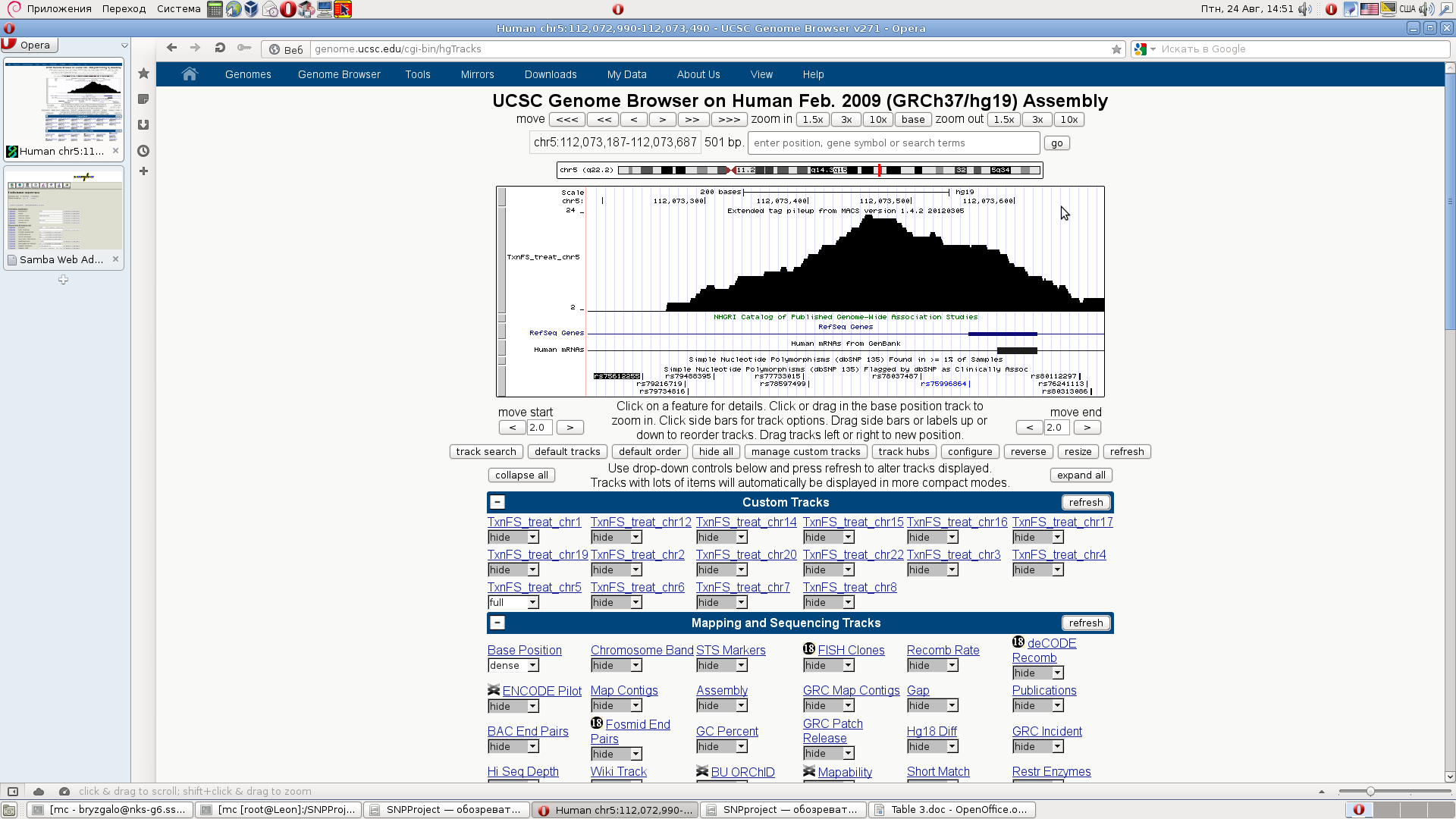 |
| rs75996864 | 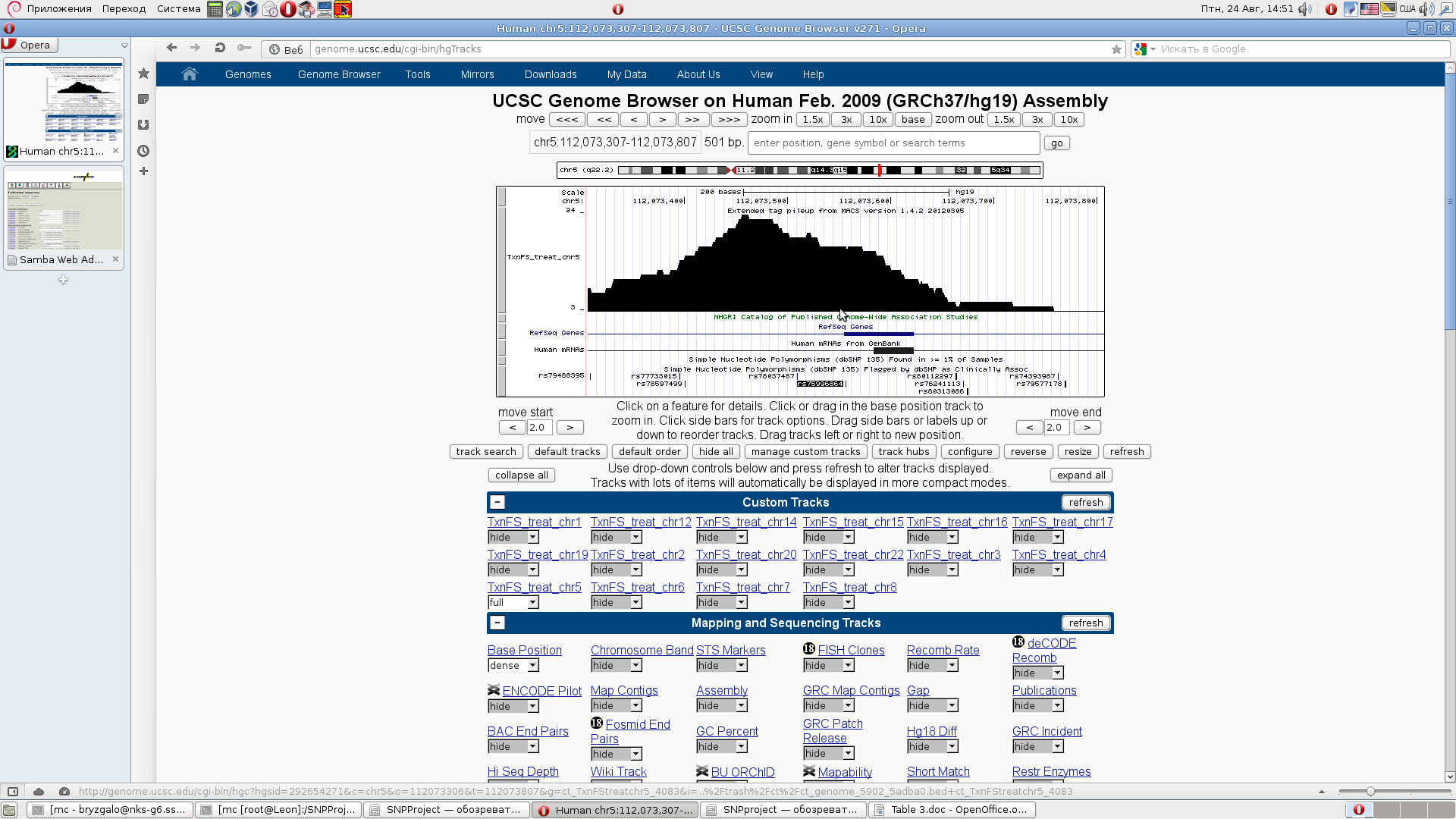 |
| rs7621113 | 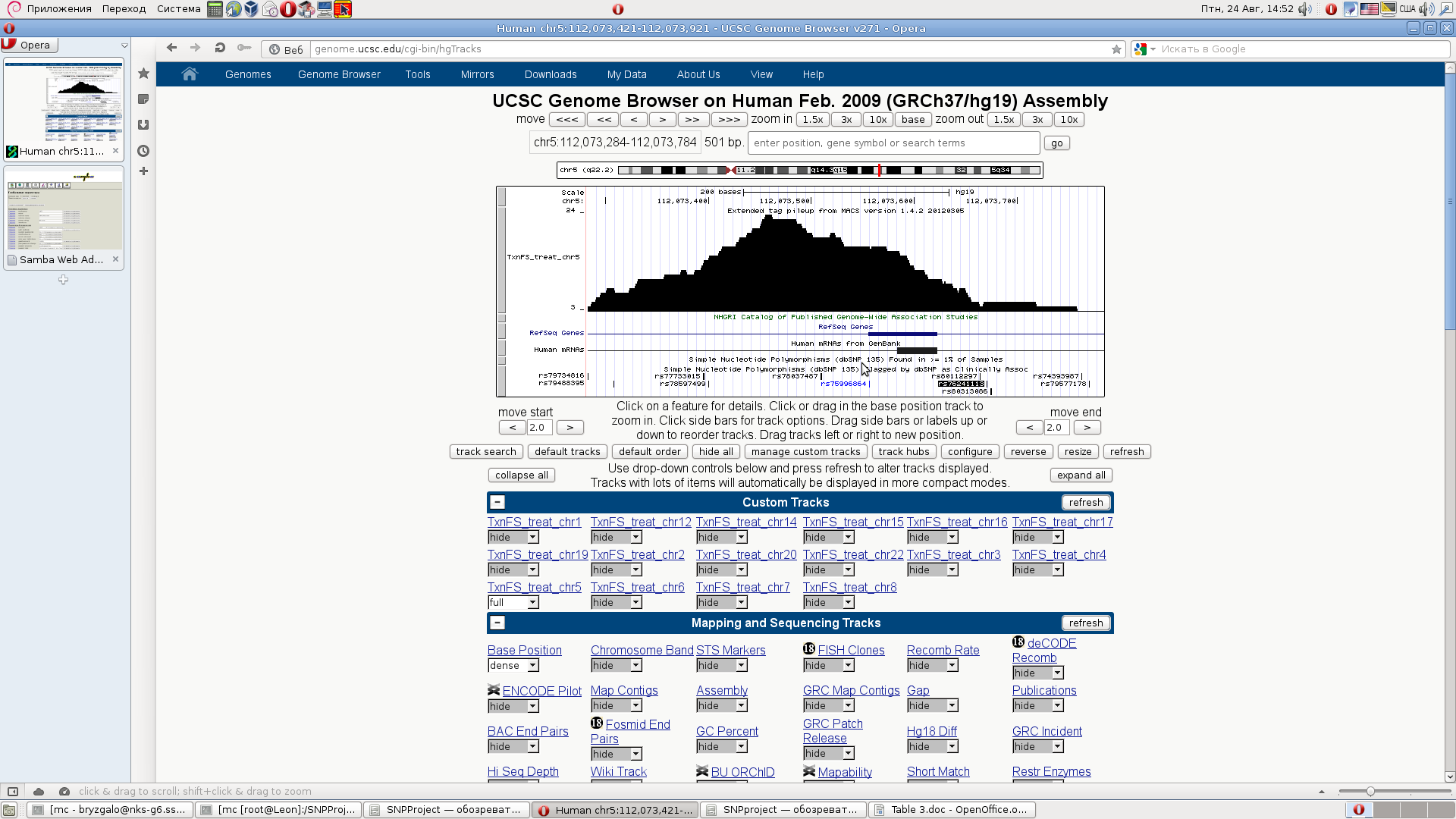 |
| rs77733015 | 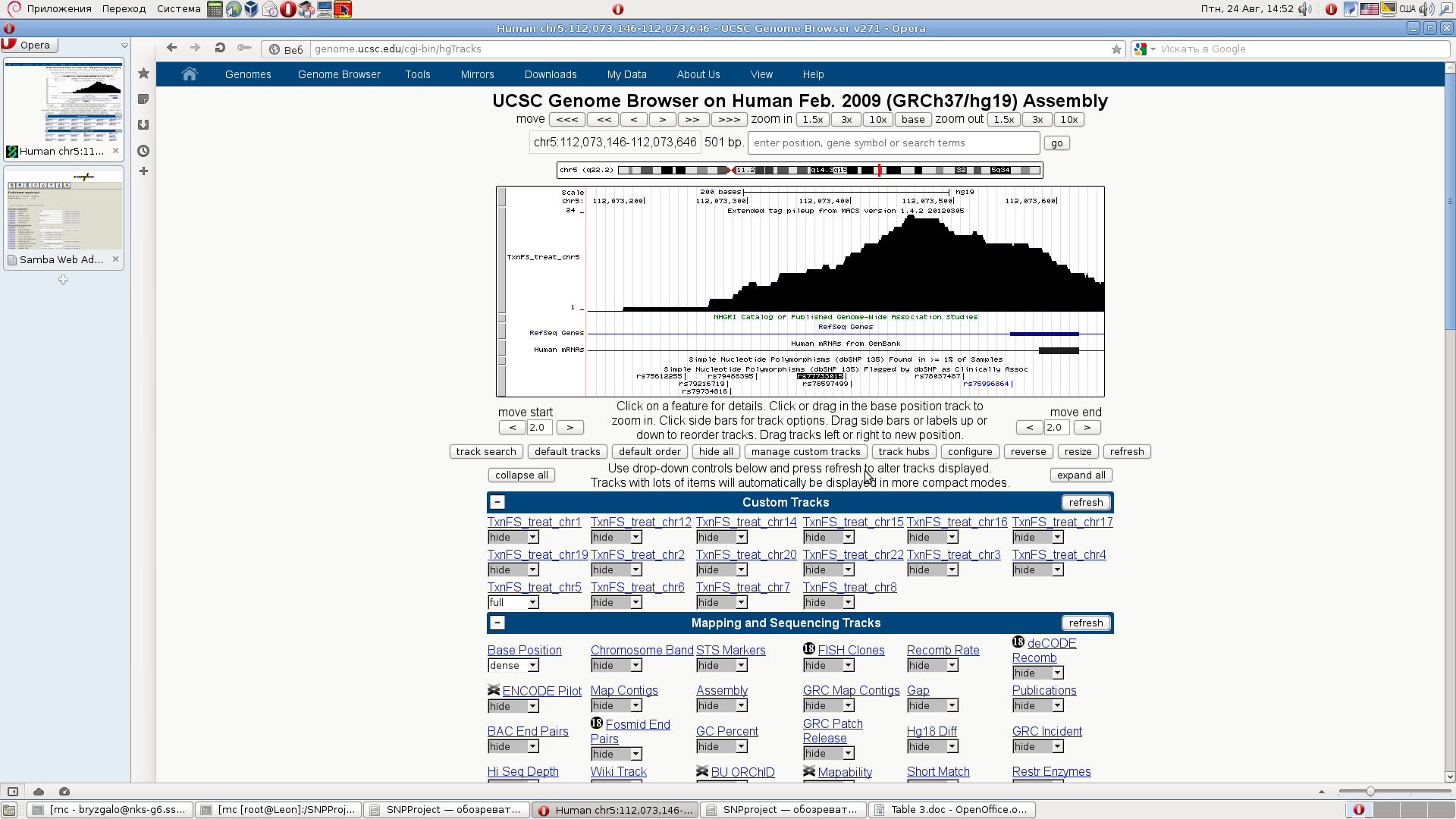 |
| rs78037487 | 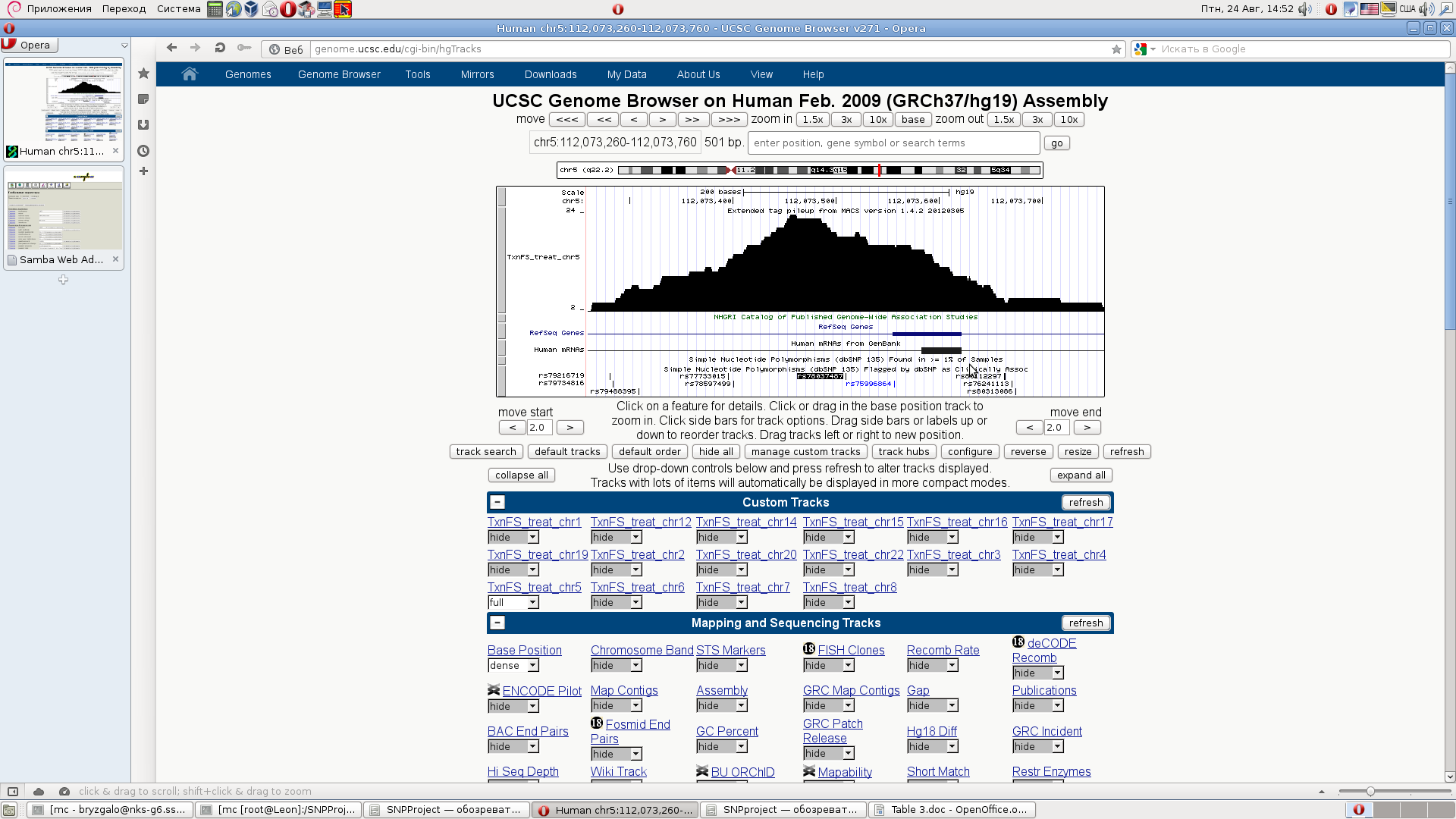 |
| rs78597499 | 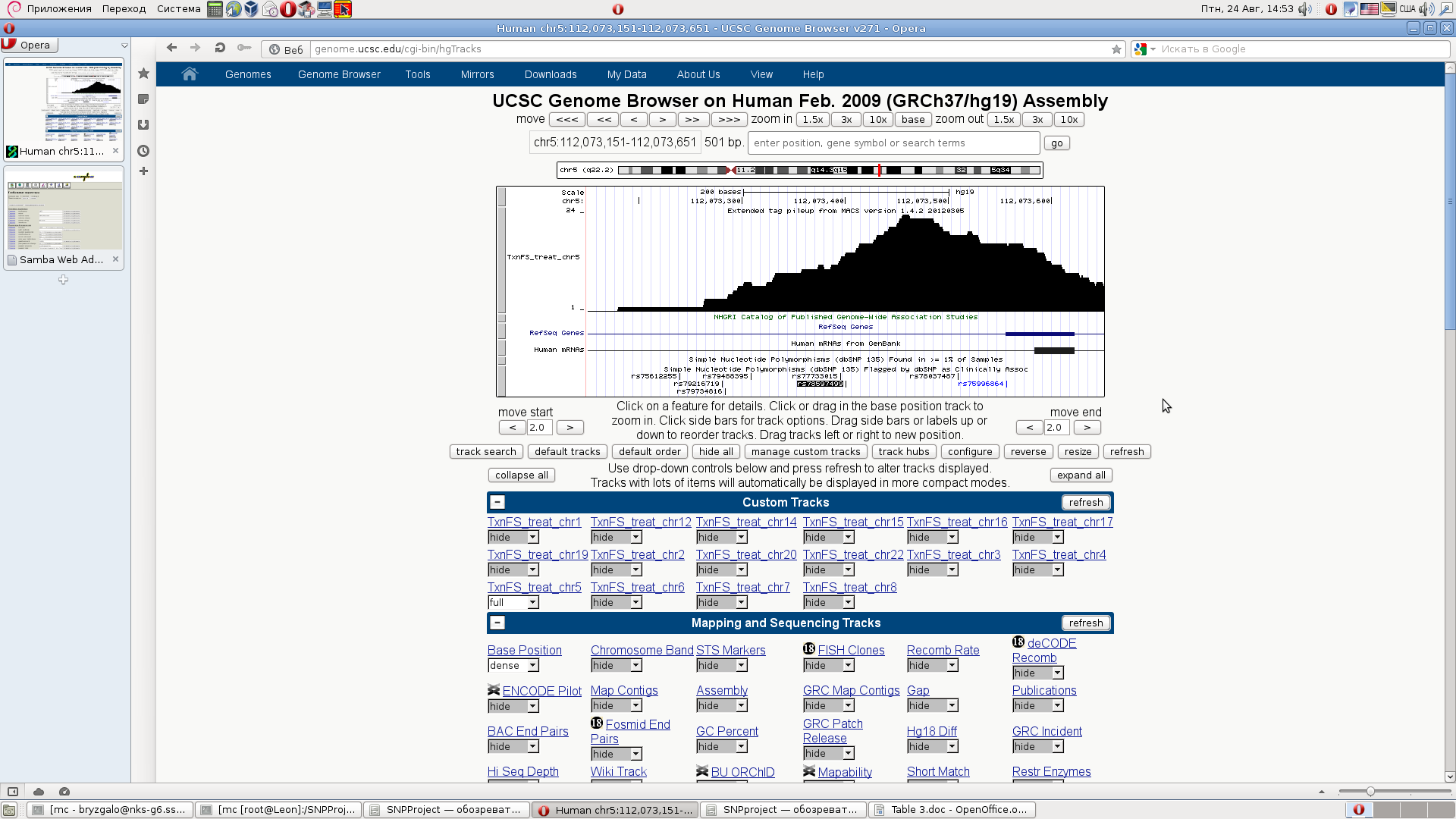 |
| rs79216719 | 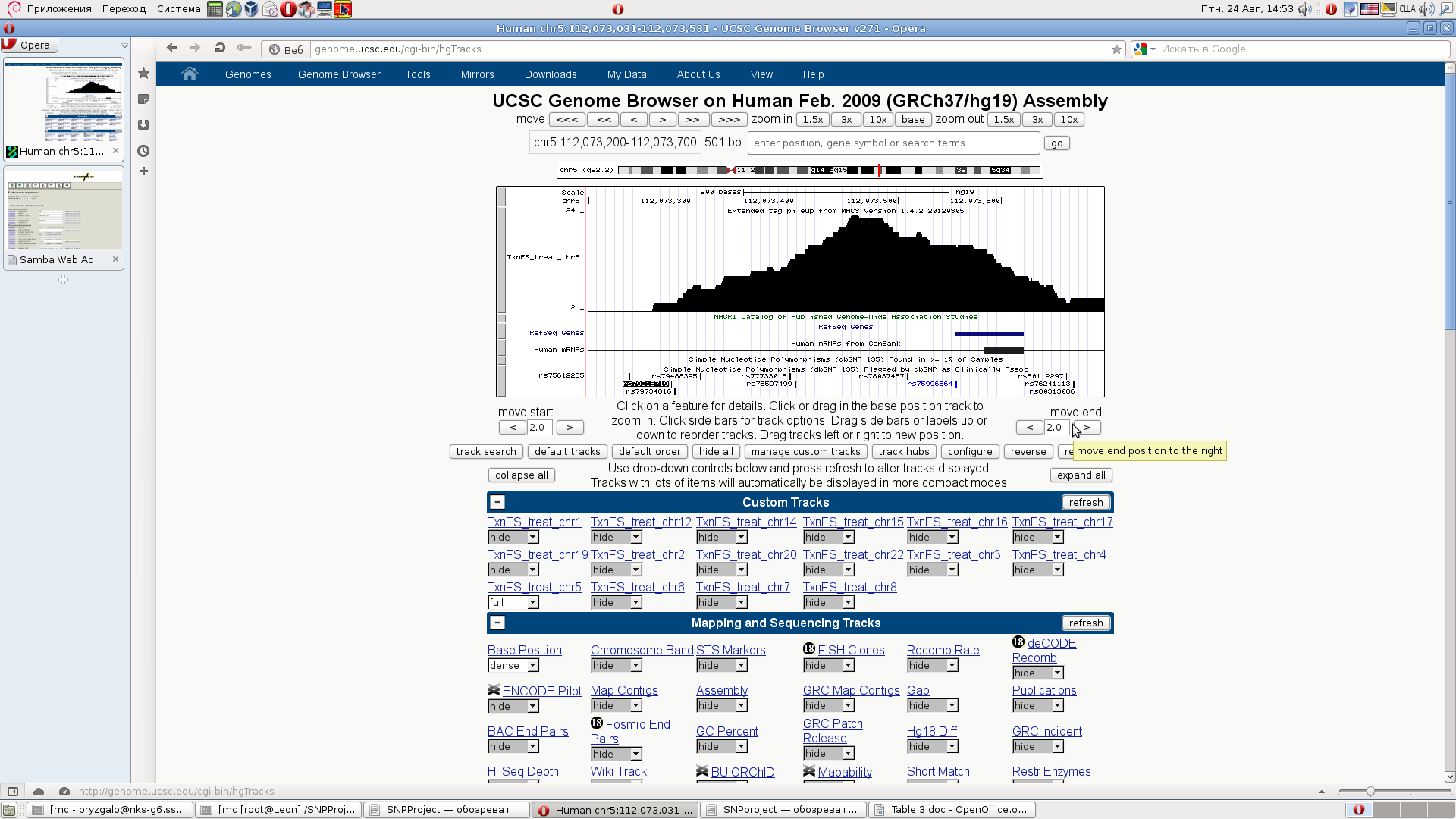 |
| rs79488395 | 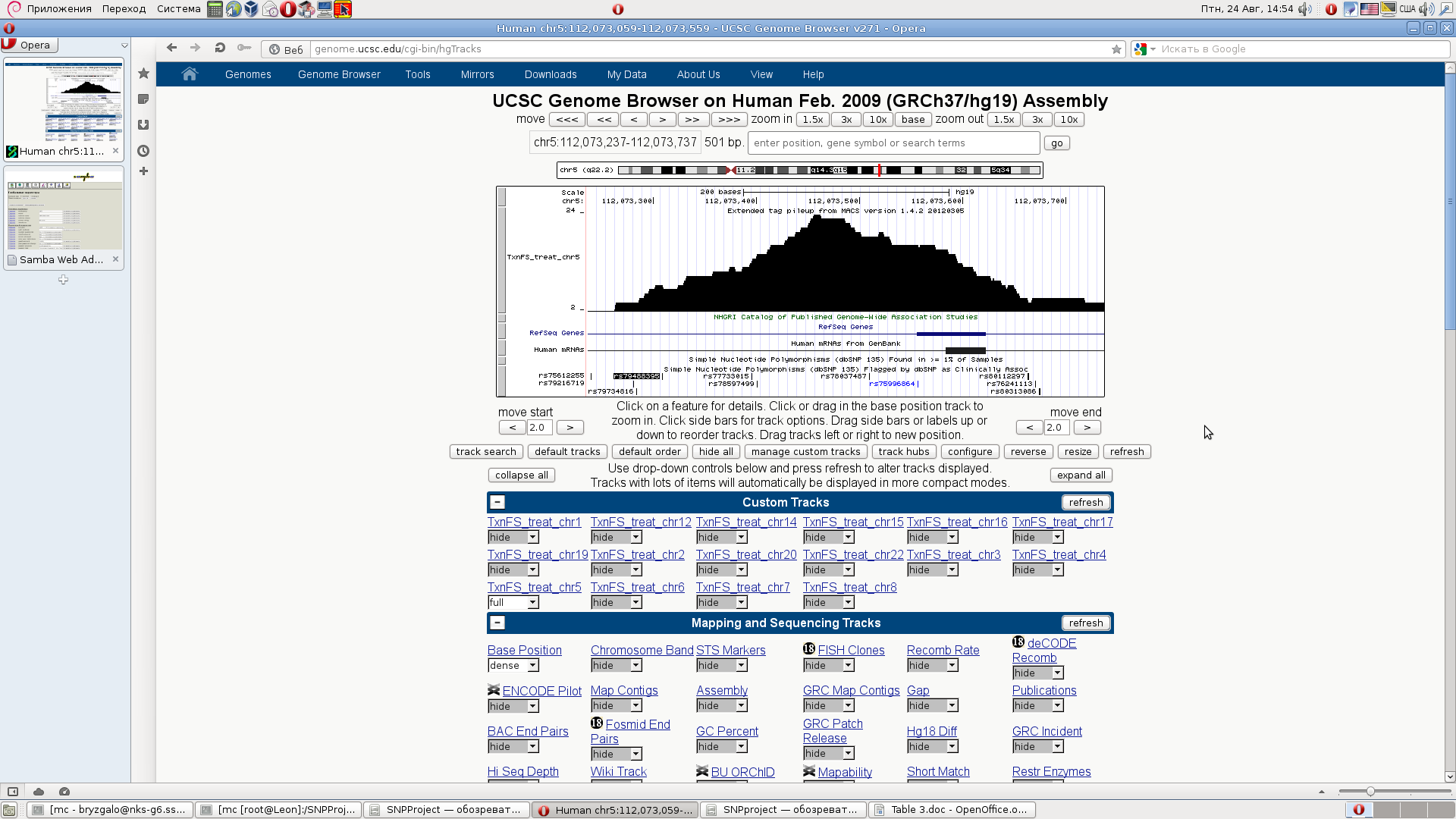 |
| rs79577178 | 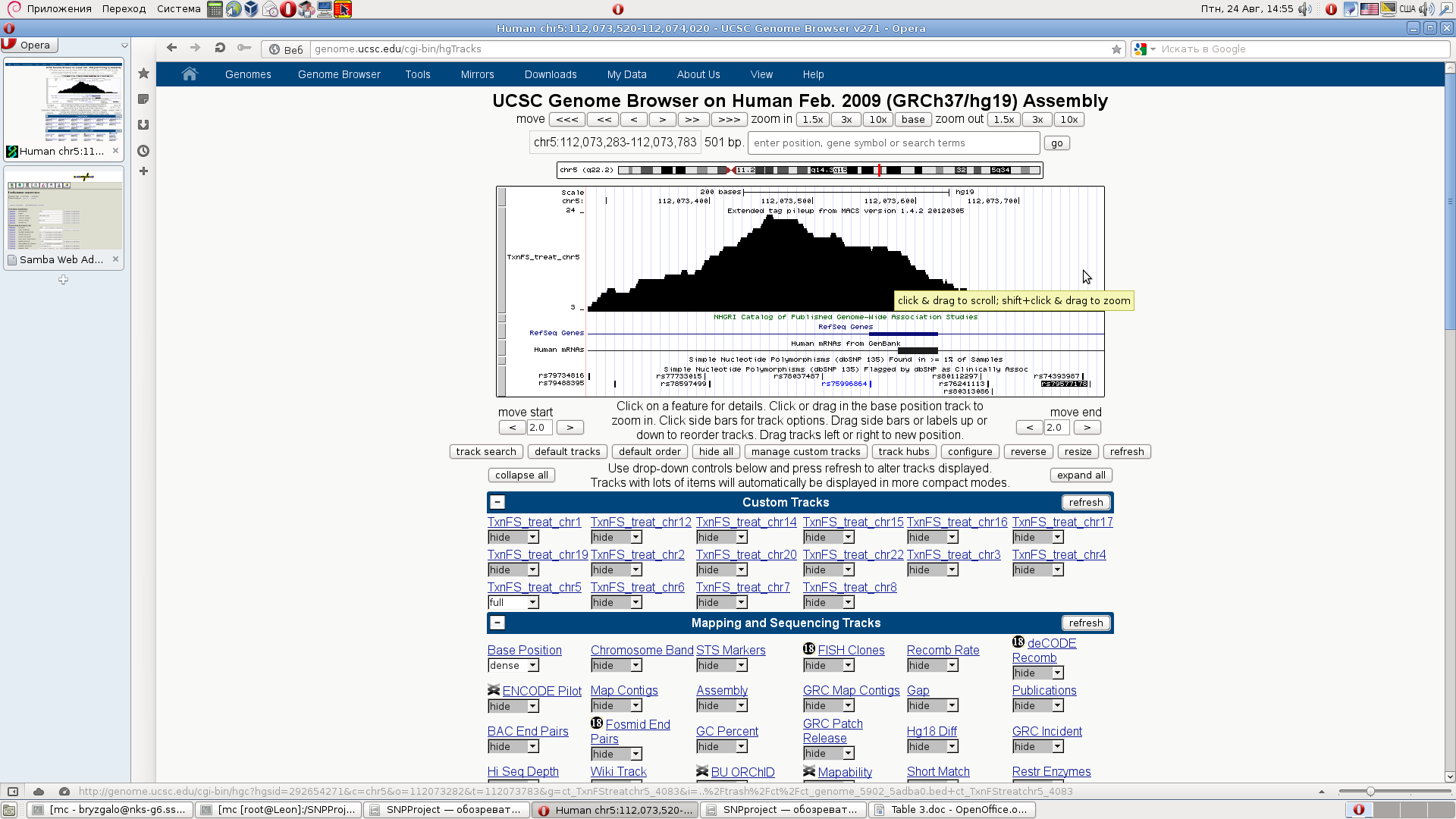 |
| rs7961894 | 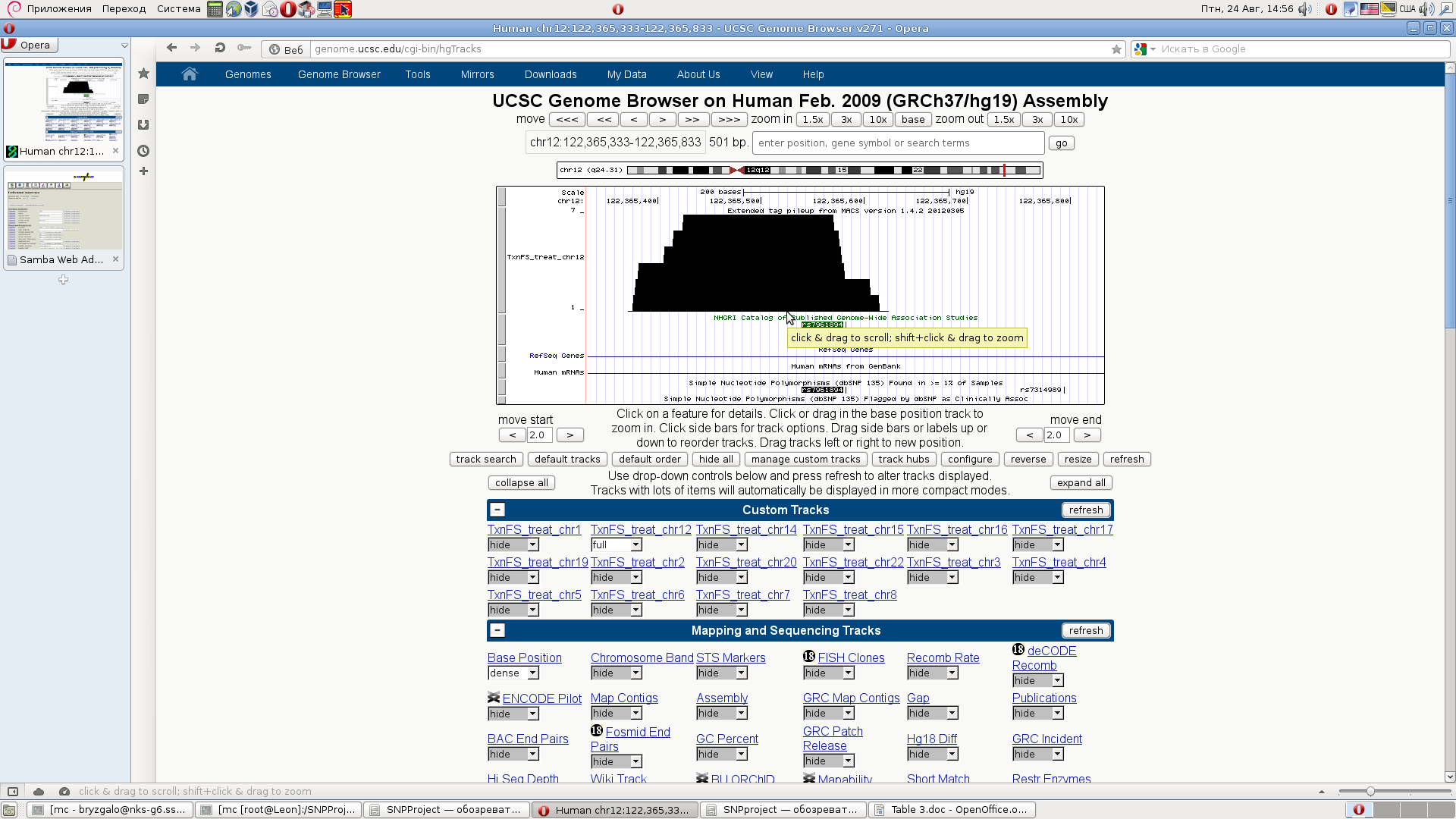 |
| rs79734816 | 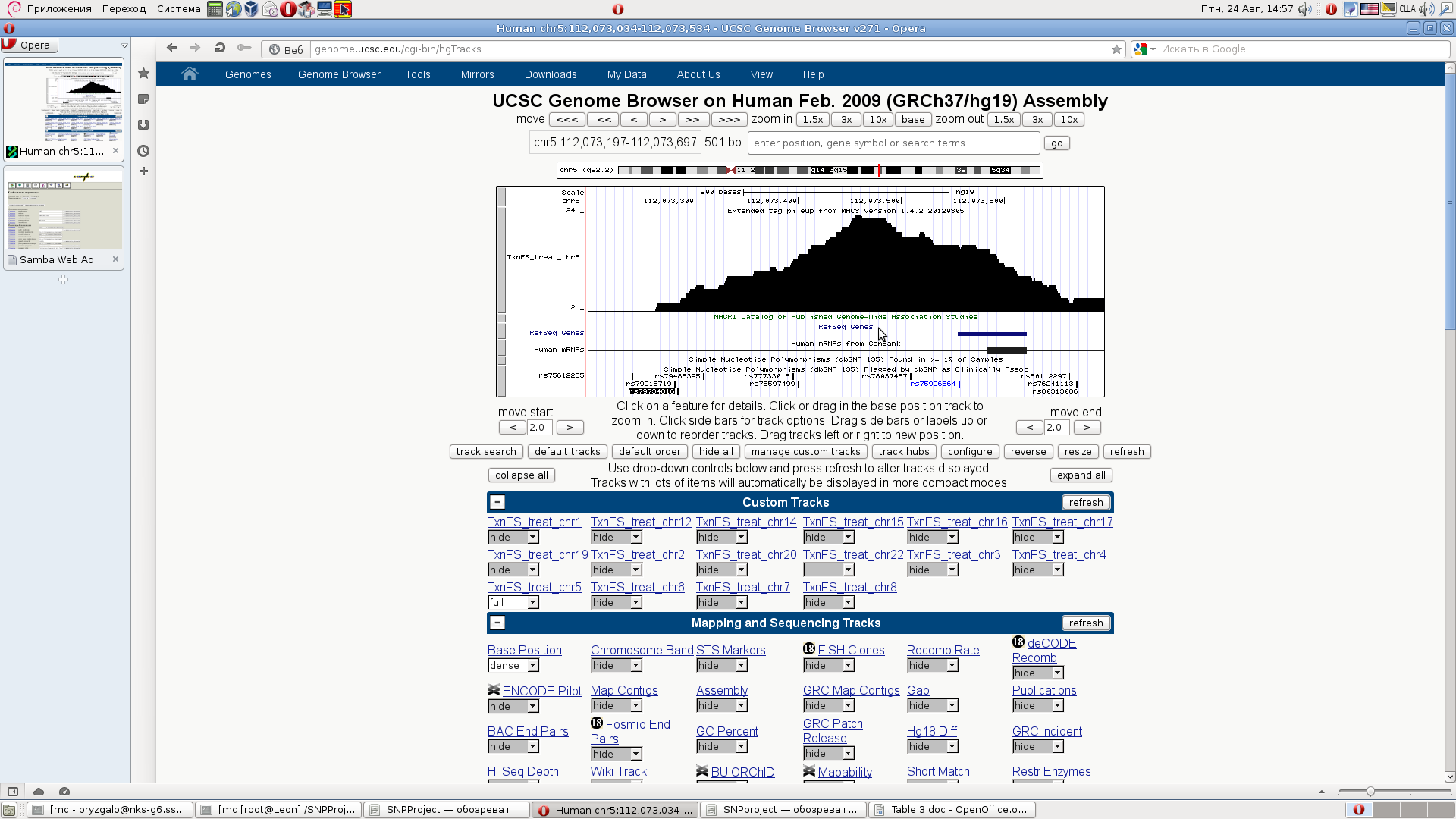 |
| rs80112297 | 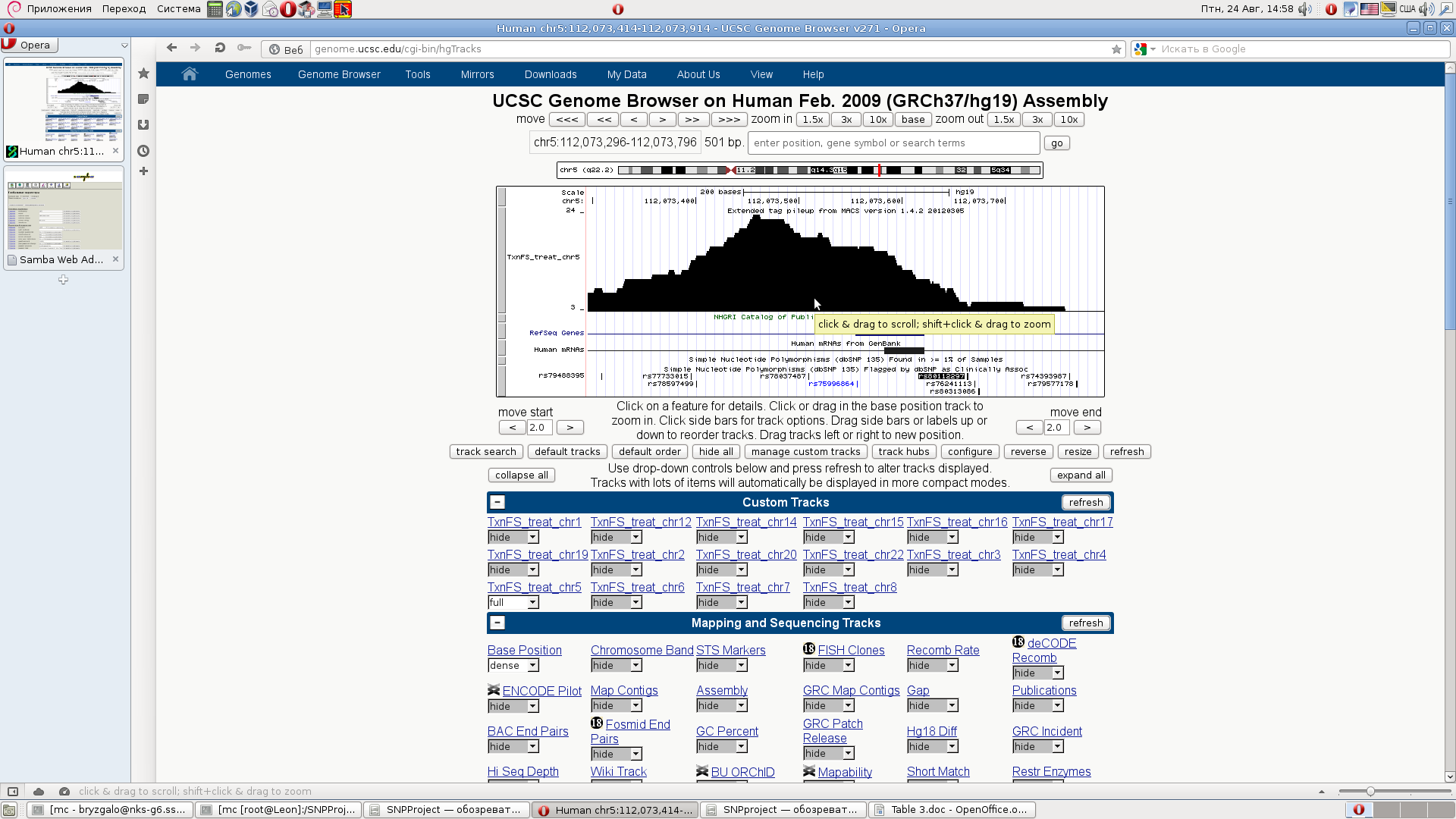 |
| rs80313086 | 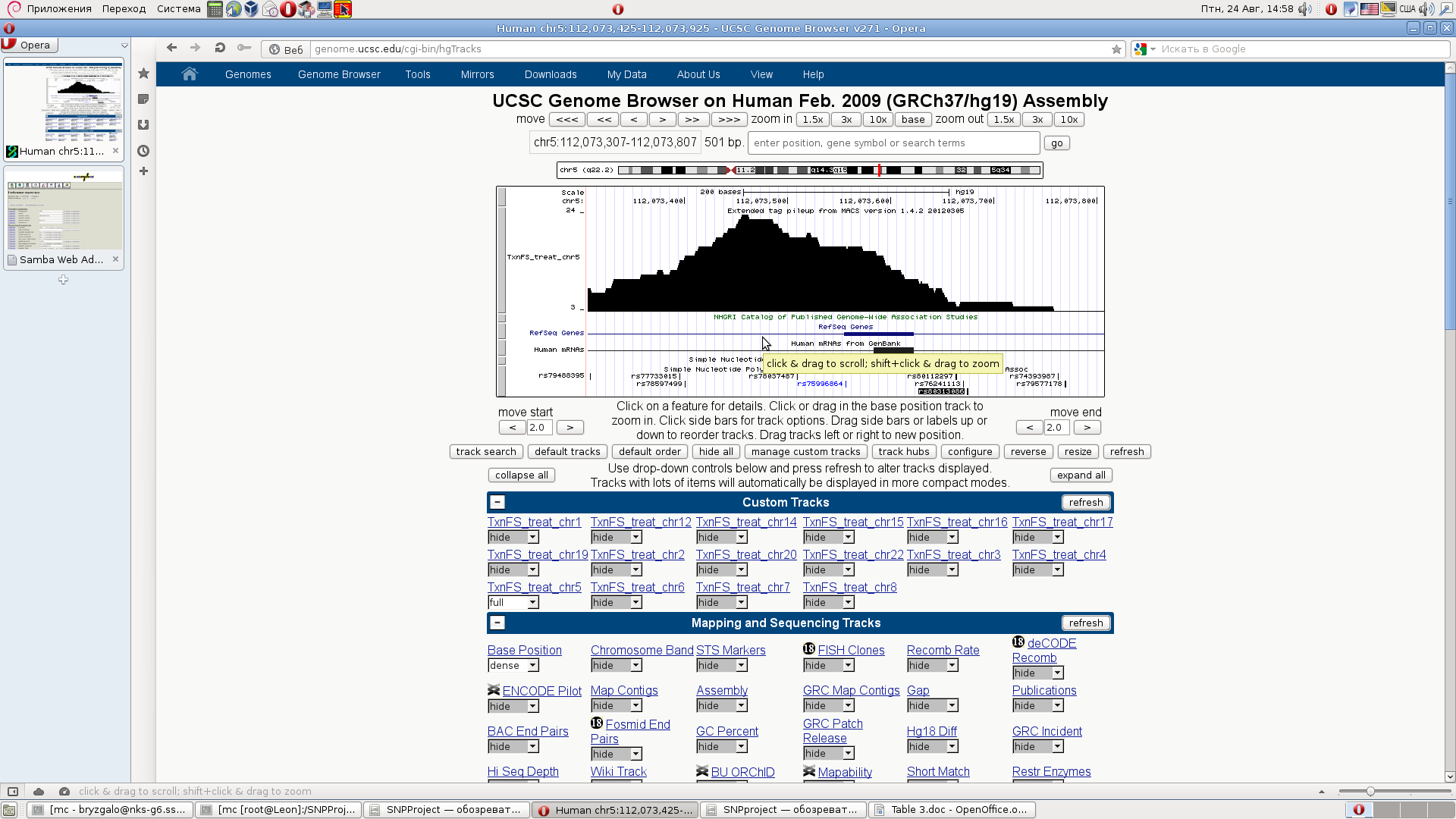 |
| rs9465871 | 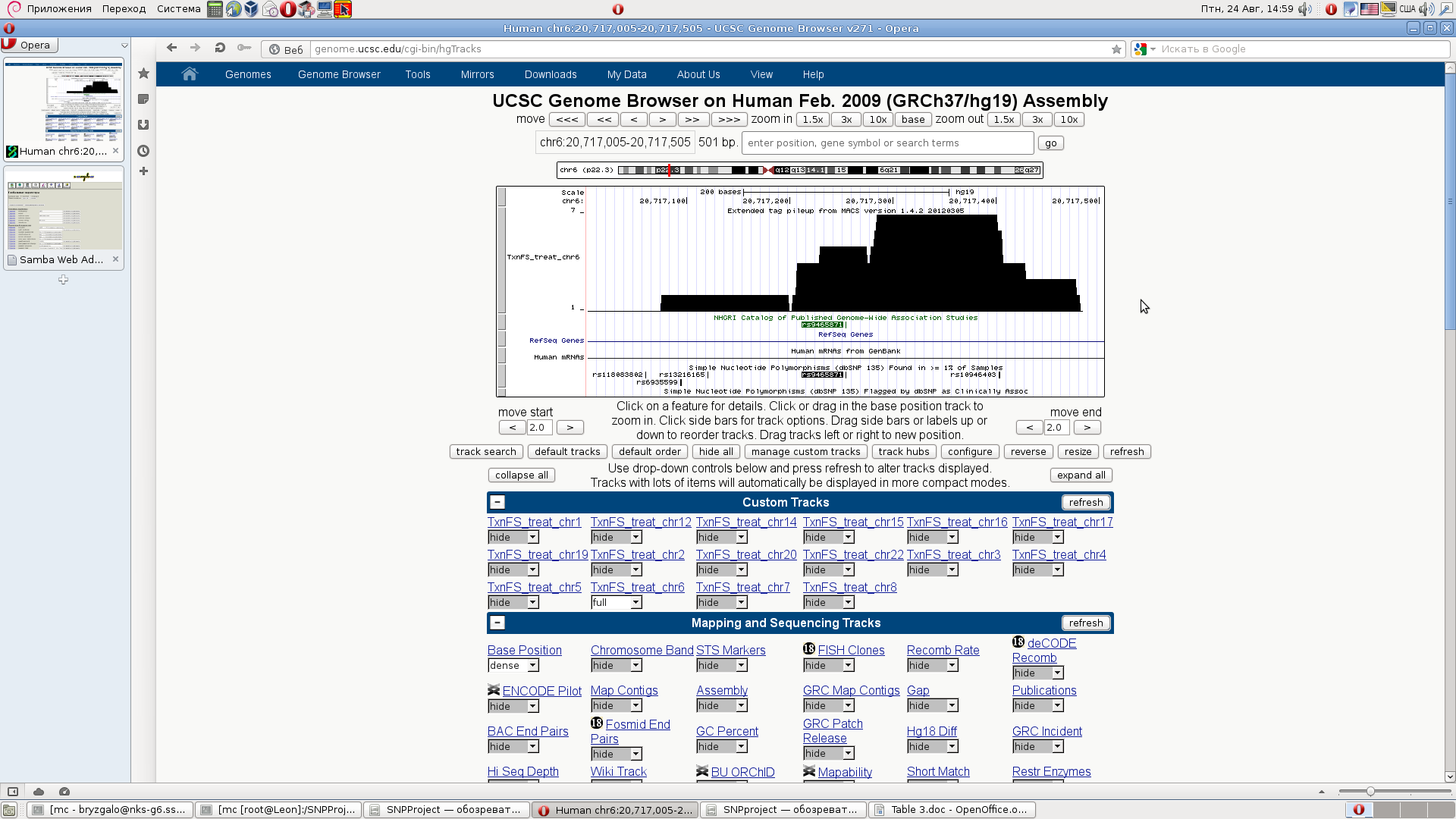 |
| rs4821544 | 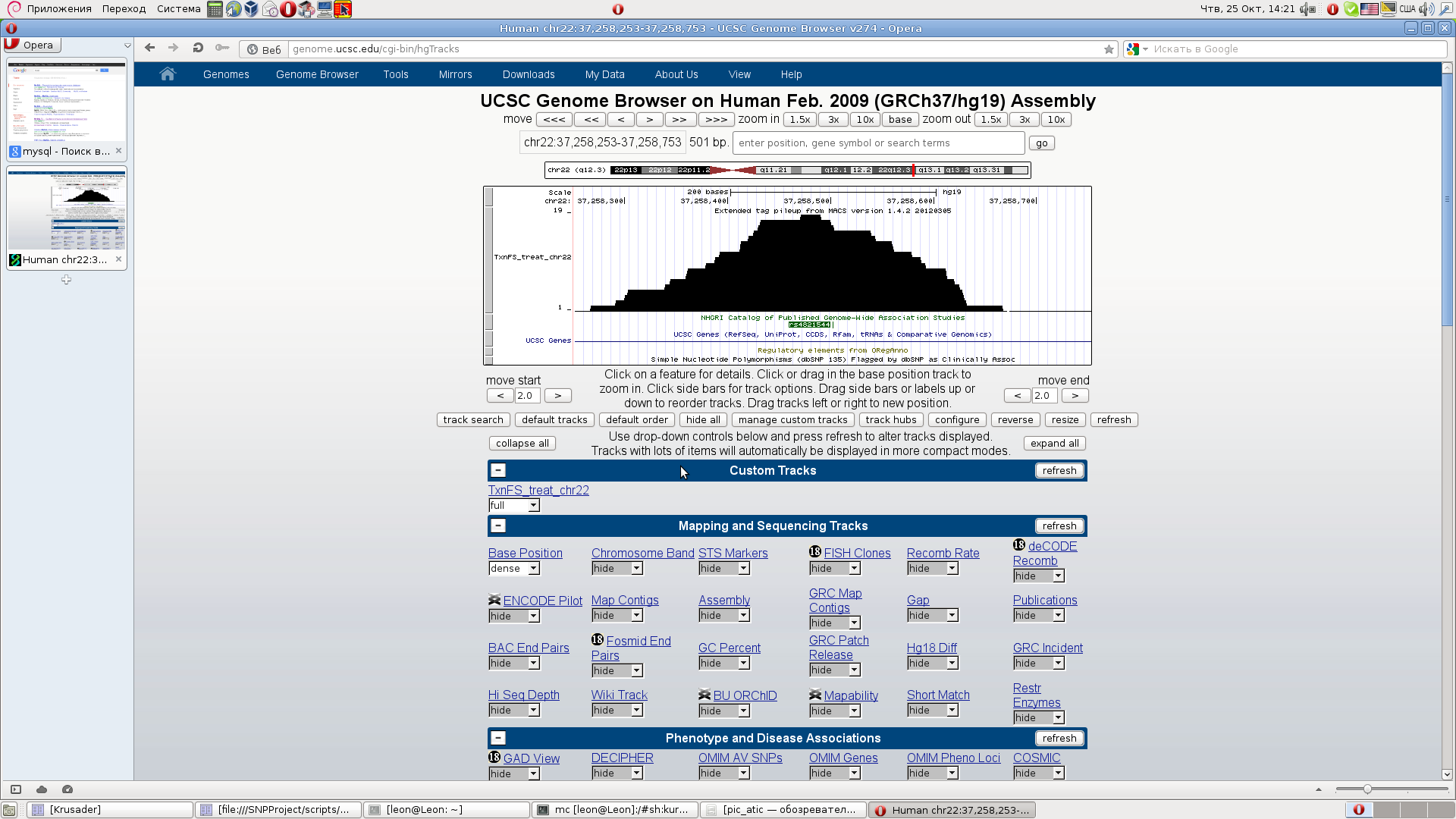 |
